# Designer phospholipid capping ligands for soft metal halide nanocrystals

**DOI:** 10.1038/s41586-023-06932-6

**Published:** 2023-12-18

**Authors:** Viktoriia Morad, Andriy Stelmakh, Mariia Svyrydenko, Leon G. Feld, Simon C. Boehme, Marcel Aebli, Joel Affolter, Christoph J. Kaul, Nadine J. Schrenker, Sara Bals, Yesim Sahin, Dmitry N. Dirin, Ihor Cherniukh, Gabriele Raino, Andrij Baumketner, Maksym V. Kovalenko

**Affiliations:** 1 Department of Chemistry and Applied Biosciences, Institute of Inorganic Chemistry, ETH Zürich, Zürich, Switzerland; 2 Empa – Swiss Federal Laboratories for Materials Science and Technology, Dübendorf, Switzerland; 3 Electron Microscopy for Materials Science (EMAT) and NANOlab Center of Excellence, University of Antwerp, Antwerp, Belgium; 4 Institute for Condensed Matter Physics, National Academy of Sciences of Ukraine, Lviv, Ukraine

**Keywords:** Materials chemistry, Surface chemistry, Inorganic chemistry, Nanoscale materials

## Abstract

The success of colloidal semiconductor nanocrystals (NCs) in science and optoelectronics is inextricable from their surfaces. The functionalization of lead halide perovskite NCs^1–5^ poses a formidable challenge because of their structural lability, unlike the well-established covalent ligand capping of conventional semiconductor NCs^6,7^. We posited that the vast and facile molecular engineering of phospholipids as zwitterionic surfactants can deliver highly customized surface chemistries for metal halide NCs. Molecular dynamics simulations implied that ligand–NC surface affinity is primarily governed by the structure of the zwitterionic head group, particularly by the geometric fitness of the anionic and cationic moieties into the surface lattice sites, as corroborated by the nuclear magnetic resonance and Fourier-transform infrared spectroscopy data. Lattice-matched primary-ammonium phospholipids enhance the structural and colloidal integrity of hybrid organic–inorganic lead halide perovskites (FAPbBr_3_ and MAPbBr_3_ (FA, formamidinium; MA, methylammonium)) and lead-free metal halide NCs. The molecular structure of the organic ligand tail governs the long-term colloidal stability and compatibility with solvents of diverse polarity, from hydrocarbons to acetone and alcohols. These NCs exhibit photoluminescence quantum yield of more than 96% in solution and solids and minimal photoluminescence intermittency at the single particle level with an average ON fraction as high as 94%, as well as bright and high-purity (about 95%) single-photon emission.

## Abstract (web summary)

Phospholipids enhance the structural and colloidal integrity of hybrid organic–inorganic lead halide perovskites and lead-free metal halide nanocrystals, which then exhibit enhanced robustness and optical properties.

## Main

**Fig. 1:**
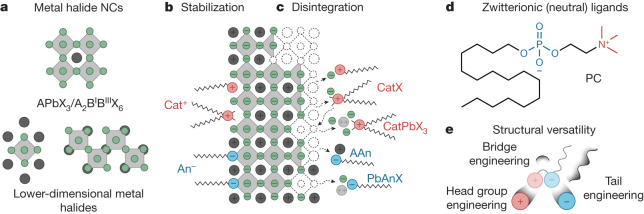
Surface chemistry of soft metal halide NCs. **a**, Examples of ionic metal halides. **b**,**c**, Atomistic depiction of surface stabilization (**b**) and disintegration (**c**) of APbX_3_ perovskite by common long-chain cationic (Cat^+^) and anionic (An^−^) ligands due to excess ligand quantity and low internal crystal energy. **d**, Zwitterionic molecules offer stronger (multidentate) binding and reduced reactivity owing to their neutral charge. **e**, Structural engineering of the head, bridge and tail groups unlocks their broad utility for stabilizing diverse metal halide NCs and dispersing them in various media.

Lead halide perovskite (LHP) nanocrystals (NCs), of the general formula APbX_3_ (A = Cs, MA, FA (FA, formamidinium; MA, methylammonium); X = Cl, Br, I), receive immense scientific and practical interest as narrow-band emitters for displays or as quantum light sources^8–13^, whereas other metal halides (Fig. 1a) are pursued as broadband emitters for solid-state lighting, scintillation and thermometry^14–16^. An imminent challenge impeding the progress in the chemistry and applications of many metal chloride, bromide and iodide NCs is that strongly binding ligands readily outcompete the relatively low internal lattice energy^17^. For instance, typical anionic and cationic surfactants that attach to halide NC surfaces in an ionic manner, displacing surface ions with the ligand head groups (Fig. 1b)^4,18–20^, also readily engage in adverse solubilization equilibria with the ions constituting the inorganic NC cores (Fig. 1c). This problem culminates with hybrid organic–inorganic perovskite compositions—MAPbX_3_ and FAPbX_3_ NCs. We proposed that zwitterionic, hence charge-neutral, capping molecules offer a general mitigation strategy for avoiding these adverse ionic metathesis reactions with NC cores, as motivated by our recent work on CsPbBr_3_ NCs with several commercial long-chain zwitterions (phosphocholines (PCs) (Fig. 1d), γ-amino acids and sulfobetaines)^21–23^. Herein, we sought to design and implement a library of phospholipid capping ligands (Fig. 1e) to broaden the compositional scope of metal halides available in the form of finely engineered NCs. Notably, only through this approach were we able to produce surface-robust, highly emissive MAPbX_3_ and FAPbX_3_ NCs. The zwitterionic group engineering was computationally guided by assessing ligand–surface binding with molecular dynamics (MD) simulations. Solid-state nuclear magnetic resonance (NMR) and Fourier-transform infrared (FTIR) spectroscopy then corroborated the atomistic models. Our design of chemically pure phospholipids with various head, bridge and tail groups leverages facile synthesis methods developed over the past few decades^24–30^. The utility of the entire library of ligands was assayed through post-synthetic anchoring to the NC surfaces. The vast molecular engineering of phospholipids endowed the compatibility of ligand-capped NCs with solvents of diverse polarity, ranging from hydrocarbons to alcohols, and enhanced luminescent properties in thin films and at the single particle level.

## Determining the binding mode of zwitterionic ligands

**Fig. 2:**
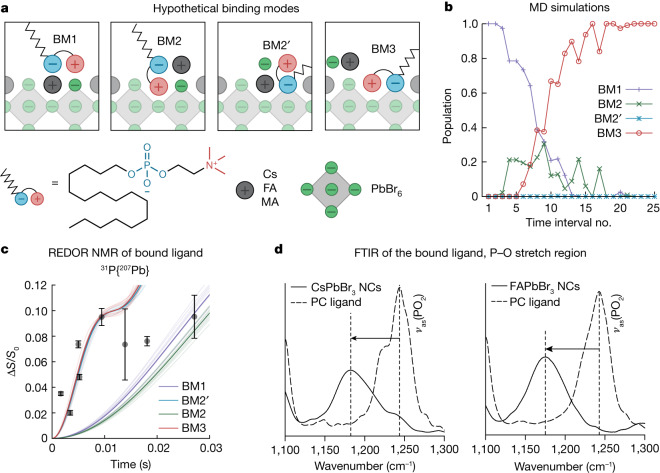
Binding of zwitterionic ligands to the FAPbBr_3_ NC surface. **a**, Schematics of different modes for binding of zwitterionic ligands, whose plausibility was assessed with replica-exchange MD simulations. **b**, Evolution of the BM populations, computed in a 50-ns-long replica-exchange MD simulation of a single PC ligand that was initially placed on the pristine FABr-rich (100) surface of FAPbBr_3_. BM3 prevails in the ensemble in the late stages of the simulation. **c**, Results of a REDOR NMR experiment for ^31^P–^207^Pb coupling supports the theoretical prediction of surface anchoring with the phosphate group. Theoretical REDOR curves were calculated using conformations obtained from the MD simulations. **d**, FTIR spectroscopy analysis of the (P–O) stretching region, wherein the P–O bond weakening on ligand binding (that is, P–O–Pb bridge formation) shifts the signal to lower frequencies. ν_as_, asymmetric stretching vibration.

**Fig. 3:**
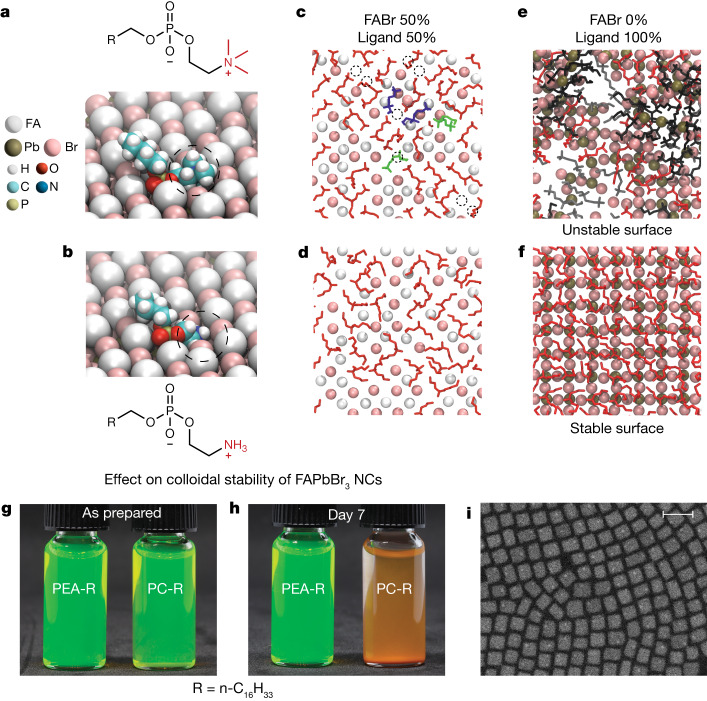
Ligand head-group engineering. **a**,**b**, Geometries of the PC (**a**) and PEA (**b**) head groups on the (100) surface of FAPbBr_3_, emphasizing the improved PEA fitness for the surface A-site. **c**–**f**, Snapshots from replica-exchange MD simulations of the FAPbBr_3_ surfaces in which 50% (**c**,**d**) or 100% (**e**,**f**) of FABr pairs were substituted with the ligands. Ligand molecules are colour-coded according to their BM—BM1 (blue), BM2 (green), BM2' (orange) and BM3 (red). Although stable surfaces are observed at 50% substitution for both ligands, PC (**c**) and PEA (**d**), a noticeable number of PC ligands and FA and Br ions desorb from a PC-capped surface, leaving behind vacancies in the top-most surface layer (black dashed circles in **c**). At 100% substitution, the PC-capped surface starts to rupture (**e**), whereas it remains stable in the case of the PEA ligand (**f**). **g**,**h**, Colloids of purified hexadecyl-PEA-capped and hexadecyl-PC-capped purified FAPbBr_3_ NCs (8.5 nm), as prepared (**g**) and after 7 days (**h**). **i**, Typical HAADF-STEM image of FAPbBr_3_ NCs capped with the alkyl-PEA ligand. Scale bar, 20 nm.

**Extended Data Fig. 1:**
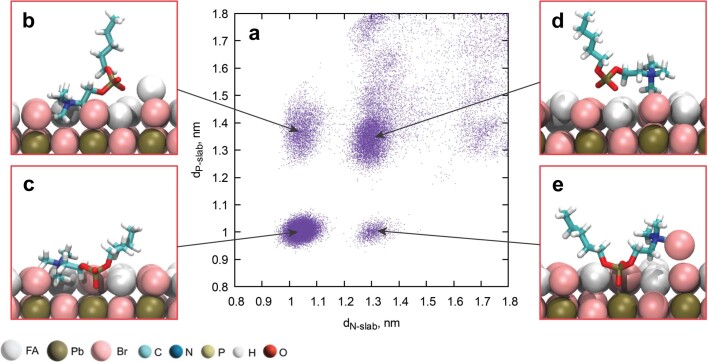
Classification of ligand binding modes. **a**, A typical map of configurations observed in replica-exchange MD simulations of the PC ligand on the (100) FAPbBr_3_ surface. d_N-slab_ and d_P-slab_ define distances from the ligand head groups (nitrogen and phosphorus atoms, correspondingly) to the middle atomic plane of the slab. Four well-defined clusters of configurations correspond to the different binding modes of the ligand (Fig. 2). The diffuse region at large ligand-slab separations corresponds to free ligands. **b–e**, Snapshots of the observed binding modes - BM2 (**b**), BM3 (**c**), BM1 (**d**) and BM2′ (**e**).

**Extended Data Fig. 2:**
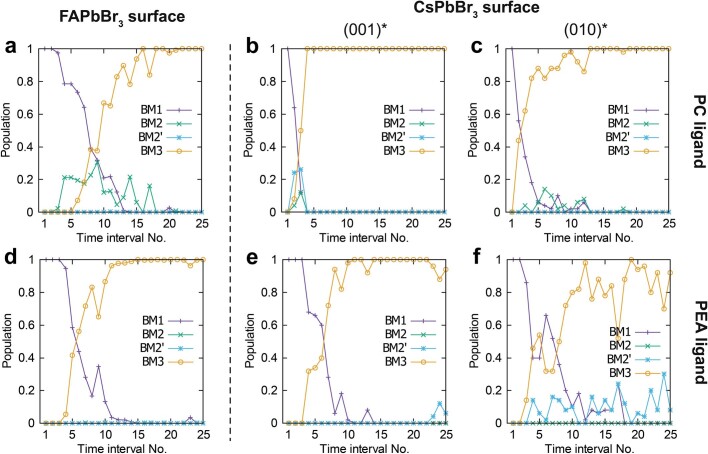
Generality of the prediction that zwitterionic ligands tend to displace both A and X ions from the perovskite surfaces. **a–f**, Evolution of binding mode populations in replica-exchange MD simulations of single PC (**a,b,c**) and PEA (**d,e,f**) ligand molecules on the FAPbBr_3_ surface (**a,d**), (001) CsPbBr_3_ surface (**b,e**), and (010) CsPbBr_3_ surface (**c,f**). Binding with a displacement of both A and X ions (BM3) is thermodynamically preferred in all studied systems. *Crystallographic orientations refer to the primitive unit cell of CsPbBr_3_ (see Supplementary Note 1 for more details).

**Extended Data Fig. 3:**
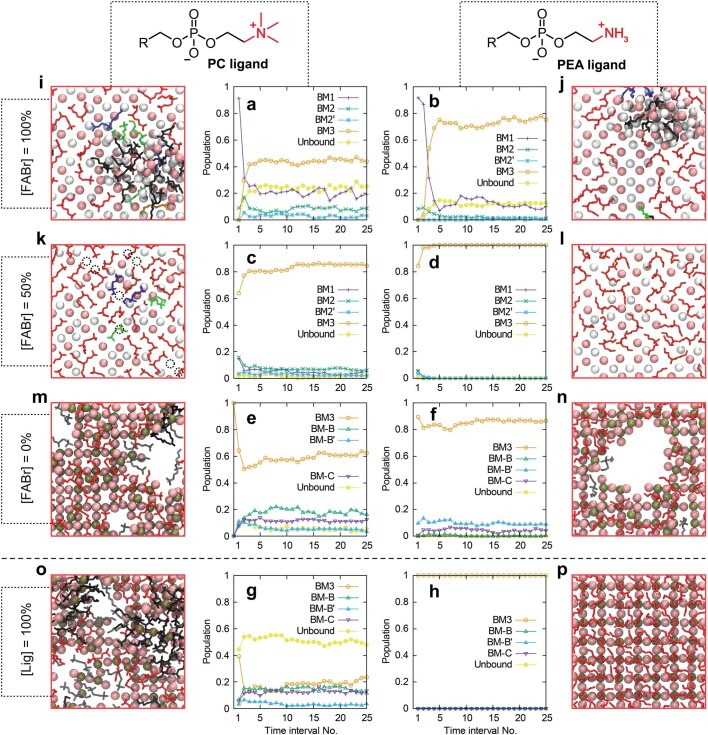
FAPbBr_3_ surfaces at various [FABr] and [Lig] concentrations. **a–f**, Evolution of binding mode populations in systems with [Lig] = 50% and with a varying amount of surface FABr – 100% (**a,b**), 50% (**c,d**), and 0% (**e,f**). **g,h**, Evolution of binding mode populations in systems with [Lig] = 100% and [FABr] = 0%. In all scenarios, BM3 was identified as a dominant binding mode. The population of BM3 is also systematically higher for PEA ligand compared to PC, indicating a better fit of the former to the FAPbBr_3_ surface. In addition, some new binding modes were discovered in systems where the rupture of the PbBr underlayer is observed — BM-B (ammonium in the PbBr layer), BM-B’ (phosphate in the PbBr layer), and BM-C (both ammonium and phosphate in the PbBr layer). However, these are marginal and are encountered only along the phase boundaries between the newly exposed FABr- and the original PbBr-terminated surfaces. **i–p**, Corresponding MD snapshots with ligands being color-coded according to their binding mode — BM1 (blue), BM2 (green), BM2’ (orange), BM3 (red), unbound (black), and other (gray).

**Extended Data Fig. 4:**
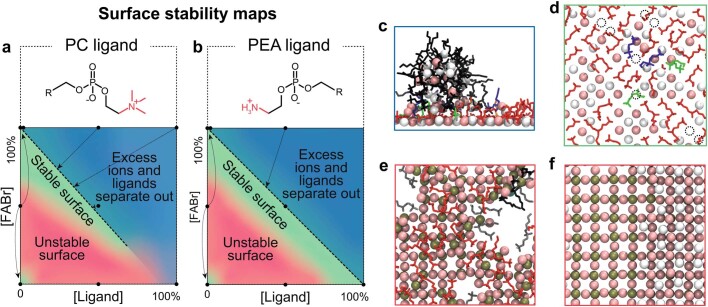
Stoichiometry-dependent stability of (100) FAPbBr_3_ surfaces. **a,b**, Surface stability maps as a function of ligand and FABr concentrations computed for PC (**a**), and PEA (**b**) ligands. **c–f**, MD snapshots that illustrate three different regions observed on the stability maps. At high ligand+FABr concentrations, excess ions and ligands separate from the surface (blue area on the map) (**c**). As a result, the system acquires equilibrium stoichiometry corresponding to the green region on the map (**d**). At low ligand and/or FABr concentrations the surface becomes unstable again (a red region on the map) — segregation into two surfaces is observed for incomplete FABr passivation (**f**), whereas partial coverage solely with the ligand causes rupture of the PbBr underlayer (**e**). Ligand molecules in the snapshots are color-coded according to their binding mode — BM1 (blue), BM2 (green), BM2′ (orange), BM3 (red), unbound (black), and other (gray).

Empirically, we have seen the efficacy of commercial long-chain zwitterions as ligands for CsPbX_3_ NCs^21–23,31^, mainly CsPbBr_3_. A champion ligand was natural lecithin (a physiological mixture of glycerophospholipids with quaternary and primary ammonium moieties), affording robust CsPbBr_3_ colloids down to small NC sizes of 3 nm. Further progress towards customizable phospholipid ligands for the entire family of LHP NCs and beyond requires a concerted computational and experimental effort to rationalize the binding of phospholipids at the molecular level. We first set out to determine whether both charged groups of the ligand participate in anchoring to the NC surface. We used a combination of classical MD simulations, FTIR spectroscopy and rotational-echo double-resonance (REDOR) NMR spectroscopy. The surface of the NCs was modelled with a slab of bulk cubic FAPbBr_3_, terminated with FABr-rich (100) crystallographic planes (Supplementary Note 1). One alkyl PC ligand was placed at 0.5 nm above the surface, and the system was solvated with toluene. During equilibration, the ligand quickly adsorbs onto the surface, assuming a conformation that is referred to as binding mode (BM) 1 (Fig. 2a). We anticipated that the ligand might also displace some of the native ions from the surface, similar to oleylammonium (OAm) binding to the surface of CsPbBr_3_ NCs^20^. Thus, three possibilities—displacement of FA (BM2), bromide (BM2′) or both FA and bromide (BM3)—are depicted in Fig. 2a. A replica-exchange^32,33^ MD simulation is then used to explore the basic BMs and to determine which of them has the lowest free energy at room temperature (Methods, Supplementary Notes 1 and 2 and Extended Data Fig. 1). The population of the starting BM1 diminishes as the simulation progresses, whereas BM3 dominates the ensemble in the late stages of the simulation (Fig. 2b). Similar results for CsPbBr_3_ surfaces indicate that A cation and the slight difference in the crystal structure do not play a substantial role (Extended Data Fig. 2). Furthermore, BM3 also prevails at other surface stoichiometries, with FABr and ligand concentrations of between 0 and 1 per binding site (Extended Data Fig. 3). Attachment of the PC ligand to FAPbBr_3_ and CsPbBr_3_ NCs through the phosphate group binding to the surface Pb atoms is attested with ^31^P–^207^Pb REDOR NMR (Fig. 2c; see Methods and Supplementary Figs. 7 and 8 for details) and by the FTIR spectroscopy (Fig. 2d, Supplementary Note 3 and Supplementary Figs. 9–12). Notably, MD simulations suggest that the relatively bulky trimethylammonium head group of the bound ligand is elevated by roughly 0.15 nm, compared with the surface FA cations (Fig. 3a and Supplementary Table 1). Therefore, we have investigated whether the surface can sustain an ever-increasing degree of FABr substitution for the PC ligand. The complete surface stability map as a function of ligand and FABr concentrations relative to the maximum possible surface coverage is presented in Extended Data Fig. 4. Although a stable surface is still observed at 50% FABr substitution, a small fraction of PC ligands, as well as FA and Br ions, do not participate in surface passivation (Fig. 3c and Extended Data Fig. 3c). Further increase of the [ligand]/[FABr] ratio leads to a complete rupture of the PbBr underlayer (Fig. 3e), suggesting that achieving greater than 50% surface coverage with the PC ligand is unlikely.

## Tailoring the head-group affinity

Rethinking the cationic moiety was a major leap in the project. Compared with the PC, an analogous zwitterionic head group with primary ammonium moiety (instead of fully methylated), namely phosphoethanolamine (PEA), allows for an excellent geometric fit on A-site (Fig. 3a,b) and theoretically allows up to 100% surface coverage (Fig. 3d,f). The absence of structural degradation or ligand desorption in simulations (Fig. 3d,f and Extended Data Figs. 3 and 4) hints that PEA is better suited for passivating FAPbBr_3_ NC under otherwise identical conditions. We also note that primary ammonium ligands, such as OAm, were the first kind of ligand used for producing monodisperse CsPbBr_3_ and FAPbBr_3_ LHP NCs^1,2^ and were shown to strongly bind to the surface A-site pockets (Fig. 1b)^20,34,35^. At the same time, such cationic–ligand binding proved highly labile owing to the acid–base equilibrium with the neutral primary amine, seen as a rapid loss of ligands and, consequently, a loss of the NC integrity on repetitive purification of NCs^34^.

**Extended Data Fig. 5:**
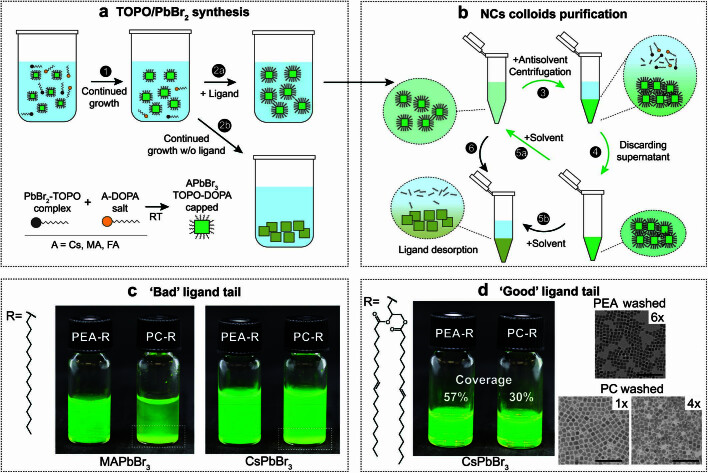
Synthesis of APbBr_3_ NCs and testing of ligands. **a**, In TOPO-DOPA synthesis^31^, PbBr_2_-trioctylphosphine (TOPO) complex reacts with diisooctylphosphinic acid (DOPA) salt of the corresponding A cation at room temperature, yielding monodisperse NCs (**1**). TOPO and DOPA are known as “bad ligands” and can be readily exchanged for zwitterion ligands (**2a**). If no capping ligands are added promptly after NCs are formed, the NCs rapidly lose their colloidal and structural integrity (**2b**). **b**, To purify phospholipid-capped NCs (see also Supplementary Note 6), a suited antisolvent is added, followed by centrifugation (**3**), whereas a supernatant containing unreacted precursors and free ligand molecules is discarded (**4**). If the ligand is’ good’, the resulting NCs pellet is redispersed in a suited solvent, yielding a stable colloid (5a). With a ‘bad’ ligand, NCs redisperse incompletely, do not redisperse (**5b**), or lose their colloidal integrity upon storage (**6**). Such a purification cycle (steps **3**, **4**, and **5a**) can be repeated several times. **c,d**, In this experiment, two head groups and two tails were compared. Combining a ‘bad’ head group and a ‘bad’ tail (**c**) leads to the worst colloidal stability, while combining a ‘bad’ head group and a ‘good’ tail might still yield long-term stable colloids (**d**). Similar to FAPbBr_3_, MAPbBr_3,_ and CsPbBr_3_ (**c**) NCs with PC head-group and a ‘bad’ hexadecyl tail precipitate after three rounds of purification, with visible deposit highlighted with a white box on the photo. CsPbBr_3_ NC colloids with both PEA or PC head-groups and a ‘good’ glycerodioleyl tail (**d**, commercially available) yield stable colloids; however, with different ligand coverage, in agreement with MD prediction. Furthermore, PC-based surface ligation is more labile, with both “good” and “bad” tails: NCs tend to increase their mean particle size (inset TEM images, scale bars 50 nm) and acquire more irregular NC morphology with storage or several purification cycles.

The computational design was put to test by synthesizing FAPbBr_3_, as well as CsPbBr_3_ and MAPbBr_3_, NCs, capped with both PC and PEA ligands. We used the trioctylphosphine oxide (TOPO)/PbBr_2_ room-temperature synthesis method to form these NCs^31^, followed by the post-synthetic displacement of weakly bound trialkylphosphine oxide and alkylphosphinic acid ligands with the ligand of choice (phospholipids), and subsequent isolation and purification of NCs (Methods and Extended Data Fig. 5a,b). PEA-ligand binding through the phosphate-group coordination to lead atoms along with the ammonium-group insertion on the surface A-site (BM3 in Fig. 2a) is confirmed by the FTIR and ^31^P–^207^Pb REDOR NMR spectroscopies (Supplementary Figs. 8–12). When comparing NCs capped with these ligands, initially stable hexadecyl-PC-capped FAPbBr_3_ NCs lose colloidal stability after several days, whereas monodisperse hexadecyl-PEA-capped NCs of the same size remain stable in colloids for months (Fig. 3g–i and Supplementary Table 13). Furthermore, MAPbBr_3_ and CsPbBr_3_ NCs exhibited an even stronger drop in colloidal stability with hexadecyl-PC ligand (compared with PEA-analogue; Extended Data Fig. 5c).

## Ligand tail engineering

The apparent colloidal stability is a combined effect of the binding group affinity to the NC surface and the structure of the ligand tail. For instance, single hydrocarbon tails (such as hexadecyl) tend to form crystalline domains and are inferior to more entropic tails comprising bent oleyl or branched hydrocarbons^36^ in instilling efficient steric repulsion. Thus, it was unsurprising to observe the efficacy of the ligand in which the PC-head group is paired with the dioleyl-glycerophosphate fragment and, likewise, the efficacy of natural lecithin comprising diverse long-chain fatty acid substituents^21,37^. Besides suited steric repulsion, the ligand shall yield long-term colloidal stability when the binding strength outcompetes the solvation-induced desorption of the ligand. For a given ‘good’ tail, transitioning from a PC to a PEA head group afforded a twofold increase in the estimated surface coverage (by NMR) and robust purification through multiple cycles of precipitation with a non-solvent, retaining uniform size and shape (Extended Data Fig. 5d).

**Fig. 4:**
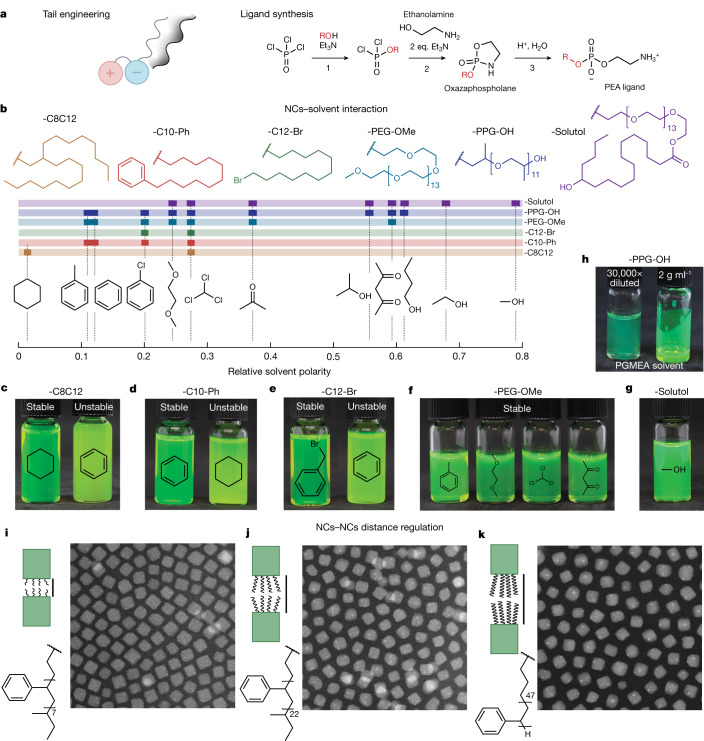
Examples of functional tail engineering. **a**, Synthesis scheme for PEA ligands tested in this work, with 21 ligand tails shown in Extended Data Fig. 6. **b**–**g**, Different tails enable matching of the solvent polarity (**b**) with highly specific dispersibility, shown for FAPbBr_3_ NCs (**c**–**e**) capped with aliphatic (**c**), aromatic (**d**), halogenated (**e**) tails and CsPbBr_3_ NCs (**f**,**g**) with polyether tails. **h**, PPG-PEA-capped CsPbBr_3_ NCs in PGMEA at a concentration of 2 g (CsPbBr_3_) per ml of dispersion. **i**–**k**, Engineering inter-NC distance in monolayers of CsPbBr_3_ NCs capped with PEA ligands with polystyrene tails by adjusting the ligand molecular weight: *M*_n_ = 900 Da (**h**), 1,200 Da (**i**) and 5,000 Da (**j**). Scale bars, 20 nm (**i**–**k**, main).

**Extended Data Fig. 6:**
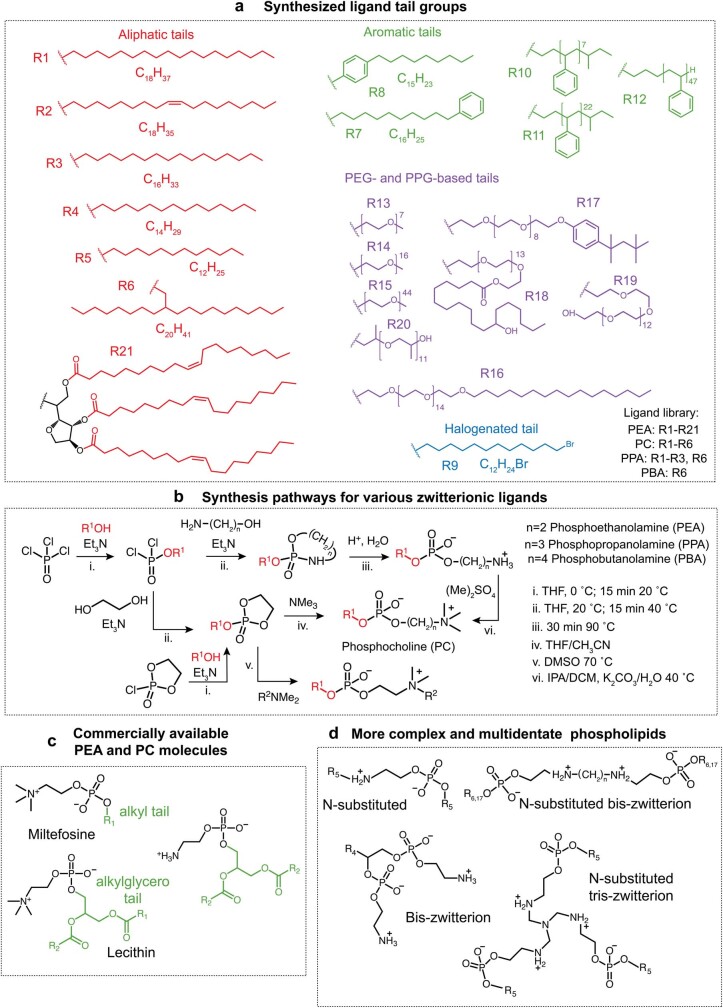
Survey of synthesized and commercial phospholipid ligands. **a**, Tail groups of the synthesized PEA ligand library. **b**, Synthetic pathways for PPA, PEA, PC and N-alkyl substituted PEA ligands. **b**, Commercially available PC and PEA ligands. **d**, Multi-zwitterionic ligands.

We thus set out to synthesize and test a library of PEA-based capping molecules, reasoning that anchoring tail groups (R) of aliphatic, aromatic, halogenated or polyether structures (Extended Data Fig. 6a) will render the resulting NCs dispersible in a broad range of common organic solvents. Synthesis of PC-terminated phospholipids with aliphatic chains has already been extensively studied with a multitude of optimized reactions^30^, owing to the prevalence of PC lipids in biological membranes^38^ and their broad use for engineering liposomes for medical applications (for example, drug or gene delivery)^24,39,40^ or as drugs on their own^41,42^. We first find that hexadecyl-PEA can be conveniently isolated by adopting the synthesis of hexadecyl-PC from hexadecanol by Eibl and Engel^29^, skipping the last methylation step. We then generalized this synthesis approach for various alcohols (ROH), converting them into preparative quantities of the respective R-PEA ligands beginning with POCl_3_ in three steps (Fig. 4a). The first two steps of gradual Cl exchange are one-pot reactions to form oxazaphospholane cycle, followed by acidic hydrolysis (Methods).

**Extended Data Fig. 7:**
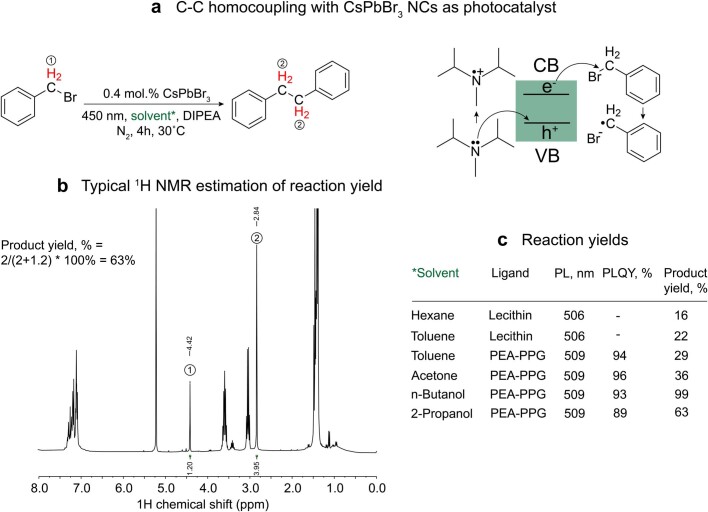
Photocatalysis with poly(propylene glycole) PEA capped CsPbBr_3_ NCs. **a**, Photocatalytic C–C coupling reaction through activation of C–Br bond in benzyl bromide, catalyzed by CsPbBr_3_ NCs. **b**, ^1^H NMR of the final reaction mixture showing peaks from the starting material (around 4.4 ppm) and product (around 2.2 ppm) and how the reaction yield was calculated from integrating the two peaks. **c**, A table summarizing product yields in different solvents and with NCs capped with either commercial lecithin or poly(propylene glycole) (PPG)-PEA.

Each of the tested ligand tails attached to a single PEA head group (21 in total; Extended Data Fig. 6a) imparted long-term colloidal stability to LHP NCs in specific solvents, echoing the ‘like dissolves the like’ principle (Fig. 4b). For each ligand–solvent pair, the purification methodology needs to be adjusted, particularly the selection of the anti-solvent (Supplementary Note 6 and Supplementary Tables 11 and 12). A branched aliphatic tail (-C8C12) is most compatible with aliphatic hydrocarbon solvents, whereas phenyl- or halide-terminated tails render LHP NCs preferentially dispersible in, respectively, aromatic or halogenated solvents (Fig. 4c–e and Supplementary Fig. 19). Dispersing LHP NCs in common polar solvents, such as acetone, alcohols or acetylacetone, without compromising their morphology had thus far remained a formidable challenge. The matter is resolved in this study using PEA-ligands with poly(ethylene) glycol (PEG) and poly(propylene) glycol (PPG) tails (-PEG-OMe, -Solutol (-PEG-OH) and -PPG-OH), all affording long-term colloidal stability and retaining monodispersity, cuboid shape and high emissivity (Fig. 4f–g and Supplementary Figs. 20–22). For instance, PPG–PEA, unlike apolar aliphatic ligands (that is, lecithin), renders FAPbBr_3_ and CsPbBr_3_ NCs highly dispersible (up to 67% by weight, inorganic basis) in propylene glycol methyl ether acetate (PGMEA; Fig. 4h and Supplementary Fig. 23), an environmentally benign solvent of broad use in optoelectronics, particularly for formulating quantum dot inks in manufacturing displays^43,44^. Another exciting avenue lies in the ability to fine-tune the inter-NC separation in NC solids, as routinely accomplished for self-assembled NC superlattices^10,45,46^. We thus have devised stable colloids with polystyrene–PEA ligands synthesized from commercial OH-terminated polystyrenes of adjustable molecular weight (0.93–21 kDa). Rigid polystyrene tails increase the inter-NC spacing to at least 5 nm in NC monolayers (Fig. 4i–k), compared with 1.2 nm with C8C12-PEA capping. Inexpensive LHP NCs are of growing interest also as photocatalysts in organic synthesis, owing to reduced carrier trapping and optical tunability^47^; yet the studies have been stalled by the lability of LHP NCs in common polar, green solvents—alcohols and ethers. PPG–PEA–ligand is an enticing ligand choice for feasibility studies, as it imparts robustness to CsPbBr_3_ NC colloids in diverse organic solvents. We conducted reductive C–C bond coupling with benzyl bromide as a substrate, previously reported with CsPbBr_3_ perovskite NCs^48^, but not with common Ir-based photocatalysts, to our knowledge. A drastic increase in the product yield (in %) was reached on transitioning from toluene (29%) to *n*-butanol (99%), for a reaction run time of 4 h at 0.4 mol% of catalyst (Extended Data Fig. 7). Lecithin-capped quantum dots, dispersible in hexane and toluene only, reached a product yield of just 16% and 22%, respectively.

## Light-emissivity of alkyl-PEA-capped APbBr_3_ NCs

**Fig. 5:**
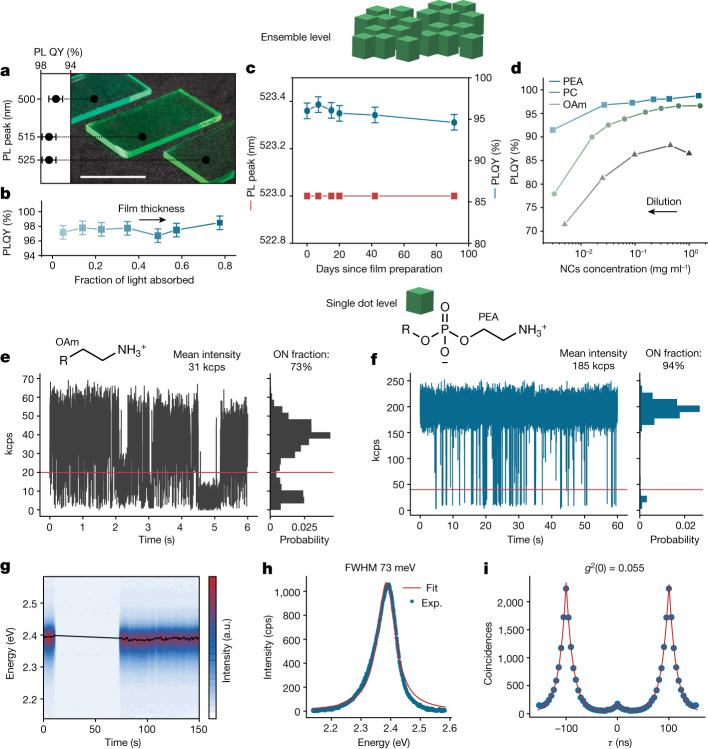
Light emission from C8C12-PEA-capped FAPbBr_3_ NCs on the ensemble and single dot levels. **a**–**d**, Highlighted optical properties of FAPbBr_3_ NCs in an ensemble. Films of FAPbBr_3_ NCs of various sizes demonstrate equally high PL QY and tunable green emission (**a**). High PL QY is retained in thick films (**b**) and is not influenced by film storage in ambient conditions for at least 90 days (**c**). PL QY of greater than 90% is preserved on approximately 1,000-fold dilution of C8C12-PEA-capped NCs with octane, whereas a pronounced drop is observed for C8C12-PC- and OAm-capped NCs (**d**), indicative of dilution-induced surface degradation. **e**–**i**, Optical properties of FAPbBr_3_ NCs at the single-particle level. Single OAm-capped NCs exhibit pronounced blinking (**e**), while single PEA-capped NCs exhibit high brightness and suppressed PL blinking (**f**), good PL stability (**g**), narrow PL linewidth (full-width at half-maximum (FWHM)) (**h**) and high single-photon purity (**i**). PL QY measurement error in (**d**) is ±1%. a.u., arbitrary unit. Scale bar, 10 mm.

TOPO/PbBr_2_ synthesis^31^ coupled with subsequent PEA-ligand capping affords highly robust colloids of FAPbBr_3_ and MAPbBr_3_ quantum dots (NC sizes down to just a few nanometres), and thus brings closer their much awaited exploration at ensemble and single-particle levels and comparison with thoroughly studied CsPbBr_3_ NCs. For instance, C8C12-PEA-capped 6 nm FAPbBr_3_ NCs exhibit unaltered optical properties after ten rounds of purification (Supplementary Fig. 25). MAPbBr_3_ NCs (10 nm) have similar stability (Supplementary Fig. 26). Compact, spin-coated films of C8C12-PEA-capped FAPbBr_3_ NCs exhibit room-temperature photoluminescence (PL) quantum yields (QYs) of 96–97% (5.5–12 nm size range, 500–525 nm PL peak range; Fig. 5a and Supplementary Figs. 29 and 30). The measured PL QY value is retained when altering the optical density (film thickness; Fig. 5b) by roughly an order of magnitude, attesting to the inherent near-unity PL quantum efficiency. Both PL QY and PL peak wavelengths of colloids and films sustain at least 3 months of storage under ambient conditions without encapsulation (Fig. 5c).

**Extended Data Fig. 8:**
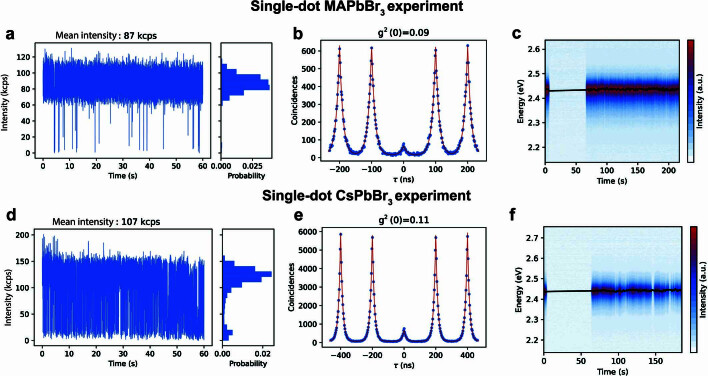
MAPbBr_3_ and CsPbBr_3_ single-dot emission at room temperature. These NCs were capped with C8C12-PEA-ligands and purified three times, followed by dilution (×10^5^) and spin-coating onto a glass substrate. **a,d,** Blinking traces of MAPbBr_3_ and CsPbBr_3_ single dots. **b,e,** Pronounced antibunching evidence high purity of single photon emission. **c,f**, Stable and narrow emission from a single MAPbBr_3_ NC (**c**) or CsPbBr_3_ NC (**f**).

Not only do purified C8C12-PEA-capped NCs exhibit higher PL QY, compared with PC and OAm ligands, but they also retain their emissivity on strong dilution of the solution (up to 1,000-fold in Fig. 5d). Far more diluted samples (×10^4^–10^5^) are required for preparing samples for single-particle spectroscopy in conventional micro-PL set-ups. Detrimental processes—ligand desorption, which caused NC aggregation, and chemical reactivity towards trace water and oxygen or even the polymer used as a matrix—are drastically aggravated on extreme dilution. Structurally labile CsPbBr_3_ NCs were reported to shrink in size, alter their morphology and surface composition and photobleach^49,50^. Fluorescence blinking—stochastic switching between bright ON and dim OFF states—are universally observed in almost all quantum emitters at room temperature. Blinking is commonly attributed to the trapping of charges on surface defects on photoexcitation, and along with PL intensity of single NCs, serves as a diagnostics of the surface electronic state. Single-dot PL data (Fig. 5e–i) evidence a profound role played by the capping ligand in achieving spectrally stable PL, suppressed blinking (94% ‘ON’ state) and high single-photon purity, with values of the second-order correlation function *g*^2^(*τ*) at zero delay time (*τ* = 0), *g*^2^(0), down to 0.055 from single C8C12-PEA-capped FAPbBr_3_ NCs. We note that achieving nearly blinking-free emission from CdSe-based NCs required epitaxial overgrowth with minimally strained lattice-matched wider-band-gap material^51–53^. For comparison, the average outcome from single OAm-capped FAPbBr_3_ NCs, which are minimally purified to reduce ligand desorption, is 73% of the time in the bright state and overall brightness of only 20–30% of the PEA-coated counterparts. These substantial differences in favour of PEA-ligated NCs, as well as the narrower dispersion of PL characteristics, are echoed by statistics over a total of 78 quantum dots (Supplementary Fig. 30). Analogous improvements have been realized also for CsPbBr_3_ and MAPbBr_3_ NCs (Extended Data Fig. 8). We note that single PEA-capped FAPbBr_3_ NCs also greatly outperform NCs capped with commercial lecithin (Supplementary Figs. 31–33). Notably, single PEA-capped quantum dots retain high brightness (about 3 × 10^5^ cps) and high ON fraction (about 90%) beyond 1 h of continuous operation (Supplementary Fig. 33b,c).

## Broader implications of phospholipid ligand capping

**Extended Data Fig. 9:**
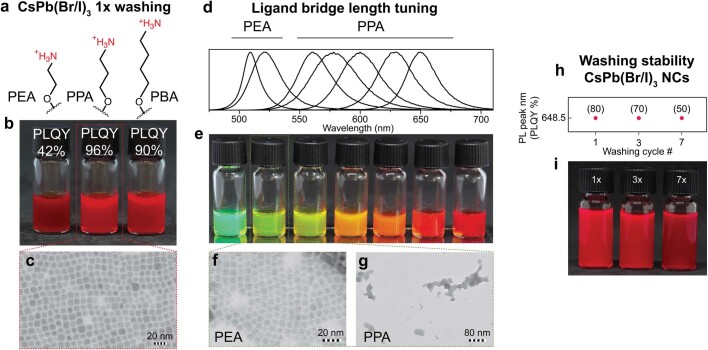
Head-group optimization by tuning the bridge length. **a–c**, Lead iodide perovskites have a larger distance between A and X surface sites than bromides. Positive-to-negative moieties distance in the zwitterion thus has a pronounced effect on the ligand binding. Mixed halide CsPb(Br/I)_3_ NCs (without anti-solvent purification, synthesis details in Supplementary Table 7) capped with phosphoalkylamine ligands featuring different distances between ammonium and phosphate functionalities (**a**): PEA, PPA, and PBA. After the first purification step with anti-solvent (**b**), PEA-capped NCs drop in PLQY from 95% to 42%, while longer-bridge PPA and PBA ligands retain high PLQY and NCs shape (**c**). **d–g**, In general, PEA ligands better suit Br-rich compositions, while PPA ligands make for a better choice for I-rich compositions. **h,i**, Mixed halide CsPb(Br/I)_3_ NCs capped with 3-ammoniopropyl (2-octyl-1-dodecyl) phosphate (*PPA-R6*) display remarkable spectral stability during purification cycles with antisolvent (ethyl acetate:acetonitrile).

**Extended Data Fig. 10:**
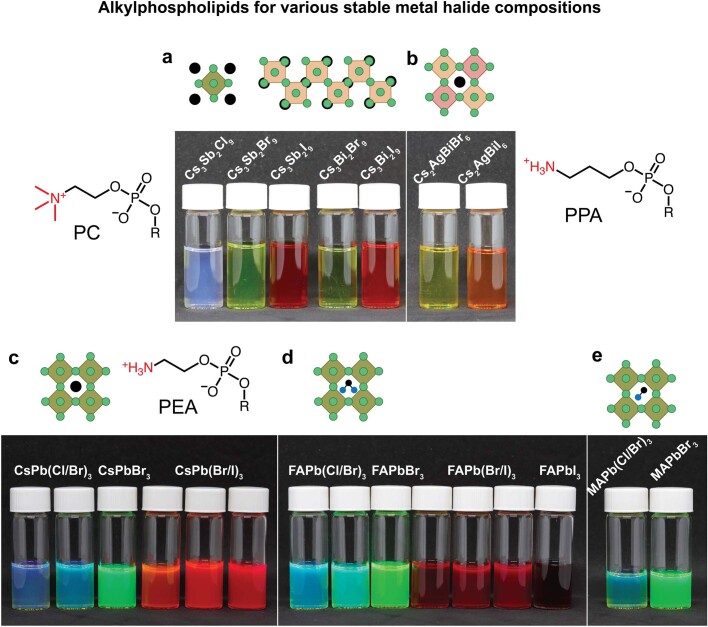
A compositional variety of metal halide NCs that can be stabilized with alkylphospholipid zwitterionic capping ligands. **a,b**, Lead-free metal halides: low-dimensional **(a**) or double perovskites (**b**). **c–e**, Stable lead halide perovskite NCs with all three cations: Cs (**c**), FA (**d**) and MA (**e**), as well as of varying halide composition, can be prepared using zwitterionic ligands with phosphate and primary ammonium head-groups and varying bridge length (PEA, PPA, PBA) (additionally see Supplementary Figs. 15–17).

Thus far, we have comprehensively presented the design of phospholipid ligation for one compositional family of LHP NCs (APbBr_3_; A = Cs, FA, MA) and a single binding head group (PEA), validated computationally and through solid-state NMR, and then synthetically paired with 21 structurally different tail groups. A far broader deployment of phospholipids as capping ligands is anticipated from the feasibility studies. For instance, adjusting the spacing between the ammonium and phosphate groups aids in matching the larger lattice constant, illustrated for iodide-rich LHP NCs (Extended Data Fig. 9), which are stable with phosphopropanolamine (PPA, C3-bridge) or phosphobutanolamine (PBA, C4-bridge), but not PEA ligand (C2-bridge). Furthermore, we used well-documented, facile and high-yield reaction schemes, exemplified in Extended Data Fig. 6b, to further extend the relevant structural space of phospholipid capping ligands. In addition to the molecules with a single head group (Extended Data Fig. 6c), molecules comprising several zwitterionic fragments (Extended Data Fig. 6d) were also validated as efficient surfactants (Supplementary Fig. 35). The broad scope of NC core materials is seen in stable colloids obtained across the entire family of LHP NCs and for the main kinds of lead-free materials—double perovskite NCs and low-dimensional Sb- and Bi-based metal halide NCs (Extended Data Fig. 10). Notably, new capping ligands can be applied not only through the post-synthetic ligand exchange, but also in the direct synthesis of NCs (Supplementary Note 7). Future studies might extend to phospholipid-stabilized colloids, oxides and fluorides, as well as two-dimensional inorganic materials such as MXenes or transition metal dichalcogenides.

## Methods

### Computational model

The surface of perovskite NCs was modelled using a crystal slab terminated with (100) crystallographic planes, in line with the high-resolution scanning transmission electron microscopy images of FAPbBr_3_ and CsPbBr_3_ NCs capped with alkylphospholipid ligands (Supplementary Fig. 5). Both ABr- and PbBr-rich lattice terminations were considered. One of the slab surfaces was then passivated by placing a certain number of ligands (1, 32 or 64) at a distance of 0.5 nm from the surface (measured to the head group). The ligand tail was truncated at five carbon atoms to reduce computational cost. If needed, some ion pairs were removed from the surface to yield the desired system stoichiometry defined by two quantities—ligand concentration $\left[{\rm{Lig}}\right]=\frac{{n}_{{\rm{lig}}}}{64}$, and ABr concentration $\left[{\rm{ABr}}\right]=\frac{{n}_{{\rm{ABr}}}}{64}$. Finally, the system was solvated with toluene. Further details of the slab model can be found in Supplementary Note 1. Interactions between ions comprising the slab were modelled by Coulomb and Lennard-Jones potentials, with parameters adopted from refs. ^54–56^. This classical perovskite model was tested in simulations of bulk CsPbBr_3_ and FAPbBr_3_ crystals with a fully anisotropic pressure coupling and yielded good agreement with experimental structural properties (Supplementary Fig. 6). The all-atom optimized potentials for liquid simulations (OPLS-AA) force field^57–59^ was used to model interactions between atoms in the organic part of the system. Ligand parameters were taken from the corresponding models of phospholipids^60,61^. Missing O–C–C–C and O–C–C–H dihedral parameters at the point of attachment of the tail to the head group were taken from the analogous dihedral parameters for ether/alcohol^62^.

### Replica-exchange MD simulations

The complete simulation boxes were equilibrated for 20 ps in the constant number, volume, temperature (NVT) ensemble with positions of all ions and ligand head groups tightly restrained and then for 1 + 10 ns in the constant number, pressure, temperature (NPT) ensemble with released restraints. In all simulations, positions of lead ions in the middle layer of the slab were restrained to the origin of the *z* axis and to their crystallographic positions in the *x* and *y* directions by applying weak harmonic restraining potential with a force constant of *k* = 1,000 kJ mol^−1^ nm^−2^, to prevent floating of the slab across the simulation box.

The final pre-equilibrated structures were used as starting points for replica-exchange MD simulations^33^ in the NVT ensemble. A total of 120 replicas were exponentially distributed between 300 and 2,200 K, allowing the efficient crossing of potential energy barriers. Exchanges between neighbouring replicas were attempted every 1 ps, and each replica was simulated for 50 ns. The average exchange rate was ensured to be above 10% for all neighbouring replica pairs. Setting all atomic masses to 12 atomic units allowed us to increase the simulation time step to 1 fs without affecting the configurational phase space of the system. Special restraining potentials were used to prevent the crystal’s high-temperature melting and limit diffusive movements of the ions and ligand molecules. These are discussed in more detail in Supplementary Note 2.

All reported simulations were performed using GROMACS software package^63^. Electrostatic interactions were computed using the smooth particle-mesh Ewald method^64^.

Binding mode populations were computed in two steps. First, distances from the ligands’ nitrogen and phosphorus atoms to the middle atomic plane of the slab, ${d}_{{\rm{N}}-{\rm{slab}}}$ and ${d}_{{\rm{P}}-{\rm{slab}}}$, were calculated over the whole replica-exchange MD run and plotted as a two-dimensional map. At most, four well-defined clusters were observed in the case of AX-terminated surfaces, which were assigned to four different BMs—BM1, BM2, BM2′ and BM3 (Extended Data Fig. 1). The broad tail that extends to long ligand-slab separations was attributed to unbound ligands. In the next step, ligands were classified according to their BM using the corresponding cut-offs on the N–slab and P–slab distances. Binding mode populations were computed as ensemble averages in 2 ns intervals and presented as time traces.

### PEA, PPA and PBA ligand synthesis

The synthesis route starting with alcoholysis of phosphorous oxychloride was adopted from ref. ^29^. Solution of alcohol substrate (0.025 mol; 1 equiv) dissolved in dry tetrahydrofuran (THF) (25 ml) along with triethylamine (0.275 mol; 1.1 equiv; 3.83 ml) is added dropwise with vigorous stirring to a solution of phosphorous oxychloride (0.03 mol; 1.2 equiv; 2.78 ml) in THF (2.5 ml) on an ice water bath. The reaction mixture is subsequently kept at 20 °C for 15 min to complete the reaction. Next, ethanolamine (0.03 mol; 1.2 equiv; 1.81 ml) and triethylamine (0.06 mol; 2.4 equiv) in THF (37.5 ml) are added dropwise under vigorous stirring to the reaction mixture kept in a room-temperature water bath. Subsequently, the mixture is heated to 40 °C for 15 min to complete the ring closure. Finally, the reaction mixture is filtered to remove precipitated triethylamine hydrochloride, and the filtrate solution is dried. An oily residue, that is, alkyl-2-oxo-1,2,3-oxazaphospholane, is dissolved in a mixture of acetic acid (5.7 ml) and distilled water (2.6 ml) at 70 °C. After 30 min, ring scission at the P–N bond is complete, and the product is separated by beating with acetone (125–150 ml). After cooling to 10 °C, alkylphosphoethanolamine is collected and dried overnight under a vacuum at 40–50 °C. For PPA or PBA ligands, 3-aminopropan-1-ol and 4-aminobutanol-1-ol were used instead of 2-aminoethan-1-ol. For PBA, the hydrolysis step was conducted at 90 °C.

### PC ligand synthesis

The synthesis procedure, beginning with the alcoholysis of 2-chloro-2-oxo-1,3,2-dioxaphospholane (COP), was adopted from ref. ^65^. A solution of COP (0.7 mol; 1equiv; 10 g) in dry THF (30 ml) was added dropwise to a mixture of alcohol substrate (0.7 mol; 1 equiv) and triethylamine (0.7 mol; 1 equiv) in dry THF (140 ml) at 0 °C under vigorous stirring. After the addition, stirring was continued for 1 h at room temperature. After filtering, the filtrate solution was concentrated by at least a factor of two or by evaporation and dry acetonitrile (150 ml) was added, and the reaction mixture was placed into a glass pressure bottle. At −20 °C, 2 M trimethylamine in THF (0.14 mol; 2 equiv; 70 ml) was added and the reaction was carried out at 70 °C for 12 h. After 12 h, the reaction mixture was cooled to −20 °C to precipitate the product. The product was then filtered off and dried under a vacuum overnight.

Further synthetic conditions and characterization of all obtained ligands can be found in Supplementary Figs. 13 and 14 and Supplementary Note 4.

### ‘Ligand-exchange’ synthesis of CsPbBr_3_, FAPbBr_3_ and MAPbBr_3_ NCs capped with zwitterionic ligands

NCs were synthesized according to the modified TOPO–diisooctylphosphinic acid (DOPA) procedure described elsewhere^31^. Pb precursor was prepared from PbBr_2_ (0.2 mmol, 73.4 mg) and TOPO (90%) (1.1 mmol, 429.6 mg) dissolved in *n*-octane (2.5 ml) at 120 °C on a hotplate in the air. Pb precursor is then diluted with various quantities of hexane to obtain NCs of different sizes. Cs precursor was obtained by reacting Cs_2_CO_3_ (100 mg) with DOPA (1 ml) in octane (2 ml) at 120 °C, followed by dilution with hexane (27 ml). FA precursor was prepared from formamidine acetate, 64 mg (0.61 mmol), DOPA (3 ml) and OA (2 ml), dissolved in *n*-octane (5 ml) at 120 °C in a 40 ml vial on a hotplate in the air. MA precursor was prepared by mixing MA in THF (2 M, 0.3 ml) along with DOPA (3 ml), OA (2 ml) and *n*-octane (5 ml). To synthesize NCs, A precursor is swiftly injected into the Pb precursor on stirring at room temperature and left for a defined amount of time to nucleate and grow NCs, followed by the addition of the zwitterionic ligands. Synthesis details, specific to NC size and composition, and further characterization can be found in Supplementary Note 5, Supplementary Tables 4–10 and Supplementary Figs. 15–17.

### Purification of zwitterion-capped NCs

When NCs remain dispersed in *n*-hexane after the ligand exchange, 2–3 equiv. of an anti-solvent (list of anti-solvents is given in Supplementary Table 11) are added to precipitate NCs, followed by centrifugation. The colourless supernatant is discarded, and the precipitate is redispersed in a desired solvent. When the ligand-capped NCs are incompatible with hexane (for example, polystyrene, polyethyglycol-based ligands), they precipitate after ligand exchange. NCs are further collected by centrifugation and redispersed in suitable solvent, completing the first washing cycle. Further, 3–4 equiv of *n*-hexane (or other suitable anti-solvent) are added to precipitate NCs again for the next washing cycle. The washing cycle can be repeated as many times as required. After three cycles, impurities of TOPO, DOPA and OA are absent, as evidenced by ^31^P NMR (Supplementary Fig. 25). Further details can be found in Supplementary Note 6 and Supplementary Figs. 25 and 26.

### Ligand coverage estimation by ^31^P NMR

To estimate the ligand coverage, stable and purified NC colloids in toluene capped with PEA or PC ligands were dissolved in dimethyl sulfoxide-d6 (DMSO-d6), destroying the NCs and freeing the bound ligands. A known amount of phosphor-containing standard (for example, tetrabutylphosphonium bromide) was added to the toluene-DMSO-d6 sample, and the ^31^P NMR one-dimensional spectrum was measured. Integration of the P signal was readily recalculated to the ligand concentration, knowing the average NC size. The NC concentration was estimated from the extinction coefficient^34^, and absorption was measured from the sample before destruction with DMSO.

### FTIR spectroscopy

Ligand BM in PEA- and PC-capped perovskite NCs was assessed with FTIR spectroscopy in conjunction with ab initio molecular dynamics simulations (details are in Supplementary Note 3). FTIR spectra of solid-state samples, that is, ligands and dry NC powders, were obtained in an N_2_-filled glovebox by means of a Thermo Fisher Nicolet iS5 FTIR spectrometer with a deuterated triglycine sulfate detector, a KBr beam splitter and an iD5 attenuated total reflectance unit comprising a diamond crystal.

### Solid-state NMR spectroscopy

^31^P–^207^Pb REDOR experiments were performed on a Bruker narrow-bore 16.4 T (600 MHz) and 9.4 T (400 MHz) NMR spectrometers equipped with a Bruker Avance III HD console. All experiments were performed on a 2.5 mm HXY probe configured in a ^1^H–^31^P–^207^Pb mode. A magic-angle spinning frequency of 20 kHz was used for all experiments. The π/2 pulse was optimized to 3 µs for ^1^H, to 6.1 µs for for ^31^P and to 6.5 µs for ^207^Pb. Chemical shifts were externally referenced to tetramethylsilane (^1^H), phosphoric acid (^31^P) and tetramethyl lead (^207^Pb). ^31^P spectra were acquired with a π-pulse excitation and ^1^H decoupling (SPINAL64) during acquisition. A recycle delay of 1 s was used. ^207^Pb spectra were acquired using a π/2–π Hahnecho sequence. The echo delay was set to one rotor cycle (50 µs). A recycle delay of 0.5 s was used.

^1^H–^31^P(^207^Pb) cross-polarization (cp) REDOR experiments were performed on a Bruker wide-bore 14.1 T NMR spectrometer equipped with a Bruker Avance III HD console. All experiments were performed on a 3.2 mm HXY DNP probe configured in a ^1^H–^31^P–^207^Pb mode. A magic-angle spinning frequency of 9 and 11 kHz was used for all experiments. The ^1^H π/2 pulse was optimized to 2.7 µs. The ^31^P π/2 pulse was optimized to 7 µs. A saturation pulse train with 16 π/2 pulses on ^1^H and ^31^P was applied. A ramp pulse (2 ms) was used for ^1^H–^31^P contact. ^1^H–^31^P cp spectra with varying recoupling times were acquired with (*S*) and without (*S*_0_) dephasing pulses on ^207^Pb. The duration of dipolar recoupling was incremented linearly. Dephasing was induced by ^207^Pb π pulses (15.2 µs). A recycle delay of 3 s was used. The recoupling curves were obtained by plotting (*S*_0_ − *S*)/*S*_0_ versus the recoupling time.

The recoupling curves $\widetilde{S}\left({N}_{{\rm{rot}}}\right)$ are determined as

$$\widetilde{S}\left({N}_{{\rm{rot}}}\right)=1-\frac{S\left({N}_{{\rm{rot}}}\right)}{{S}_{0}({N}_{{\rm{rot}}})}$$

where $S\left({N}_{{\rm{rot}}}\right)$ and ${S}_{0}\left({N}_{{\rm{rot}}}\right)$ are the integrals of the NMR spectra with and without dephasing pulses at varying recoupling times (expressed as number of rotations ${N}_{{\rm{rot}}}$). Using uncertainty propagation, the corresponding error ${\sigma }_{\widetilde{s}}({N}_{{\rm{rot}}})$ is

$$\begin{array}{l}{\sigma }_{\widetilde{s}}({N}_{{\rm{rot}}})=\sqrt{{\left(\frac{\partial }{\partial S({N}_{{\rm{rot}}})}\widetilde{S}({N}_{{\rm{rot}}})\right)}^{2}{\sigma }_{S}^{2}({N}_{{\rm{rot}}})+{\left(\frac{\partial }{\partial {S}_{0}({N}_{{\rm{rot}}})}\widetilde{S}({N}_{{\rm{rot}}})\right)}^{2}{\sigma }_{{S}_{0}}^{2}({N}_{{\rm{rot}}})}\\ \,=\,\sqrt{\frac{{\sigma }_{S}^{2}({N}_{{\rm{rot}}})}{{S}_{0}^{2}({N}_{{\rm{rot}}})}+\frac{{\sigma }_{{S}_{0}}^{2}({N}_{{\rm{rot}}}){S}^{2}({N}_{{\rm{rot}}})}{{S}_{0}^{4}({N}_{{\rm{rot}}})}}\end{array}$$

The errors ${\sigma }_{S}\left({N}_{{\rm{rot}}}\right)$ and ${\sigma }_{{S}_{0}}\left({N}_{{\rm{rot}}}\right)$ are determined from the noise level of the spectrum and are identical as number of scans and noise levels are identical.

### Computation of theoretical REDOR curves

Theoretical REDOR curves (BM1–BM3) were generated by our Python implementation of an approach reported elsewhere^20^. The source code is available at https://gitlab.ethz.ch/kovalenkolab/redor. This approach simulates REDOR curves for a multi-spin system (more than two spins), including heteronuclear coupling, while assuming that homonuclear coupling can be neglected. This assumption holds for the initial slope of the REDOR curve (short dephasing times)^66^. The geometries for the theoretical curves were either based on the crystal structure of FAPbBr_3_ or chosen from the replica-exchange MD simulation of a single PC ligand on the FABr-terminated perovskite surface. Ten randomly selected structures were used for each BM in the latter case. Because BM2′ was not observed in this simulation, a separate 10 ns plain MD simulation with the ligand initially placed in BM2′ was performed. We note that BM2′ was found to be metastable across the entire simulation. The theoretical REDOR curve on Fig. 2c was scaled by coefficient 0.3 to fit the initial data slope to account for dynamics and inefficient recoupling due to the potential broadening of ^207^Pb signal of Pb atoms bound to phosphate.

### Electron microscopy

Transmission electron microscopy images were collected using a Hitachi HT7700 microscope operated at 100 kV. High-angle annular dark field scanning transmission electron microscopy (HAADF-STEM) images were obtained using an FEI Titan Themis aberration-corrected microscope operated at 300 kV and with a probe-corrected cubed Thermo Fisher Scientific Themis Z Microscope operating at 300 kV with a probe semi-convergence angle of 20 rad. Images were processed using Image J.

### Optical spectroscopy

Room-temperature PL spectra of purified quantum dots were recorded with a Fluorolog iHR 320 Horiba Jobin Yvon with an excitation at 350 nm. Absorption spectra were recorded with a Jasco V670 spectrometer. PL QY was measured using the Quantaurus-QY spectrometer C11347-11 from Hamamatsu Photonics; for samples with absorbance higher than 0.4, a self-absorption correction procedure was used as implemented in the software (U6039-05 for Quantaurus QY). Single-dot spectroscopy and related sample preparation were conducted under a nitrogen atmosphere. First, the NC solutions were diluted by a factor of 30,000–60,000 with dry and filtered *n*-octane (99+% extra dry, Acros Organics). A sparse NC film was obtained by spin-coating 100 µl of the diluted solution at 150 reps for 60 s onto a clean cover glass (thickness 170 ± 5 μm; diameter 25 mm; Thorlabs). Micro-PL measurements were carried out with a home-build set-up under irradiation with a pulsed 405 nm laser (10 MHz, pulses less than 50 ps, greater than 100 W cm^−2^, PicoQuant). The laser is focused (1/e^2^ = 1 μm) by an oil immersion objective (numerical aperture = 1.3) onto the sample, and the same objective collects the emitted light. The collected light is passed through a dichroic mirror to filter out the residual light from the excitation laser and sent either to a Hanbury Brown–Twiss experiment or to a monochromator and electron-multiplying charge-coupled device camera (1 s binning, Princeton Instruments). The Hanbury Brown–Twiss experiment consists of a 50/50 beam splitter, two avalanche photodiodes (temporal resolution = 250 ps, Excelitas) and photon-counting electronics (PicoQuant), enabling the acquisition of time-tagged time-resolved (TTTR) fluorescence data. Single-dot measurements were carried out in the weak excitation regime at a fluence of 0.8–1.3 μJ cm^−2^ (less than one exciton per pulse). The spectra of the NCs were obtained by averaging the first five frames of the spectra series; they were then fitted with a Lorentzian peak to find the peak centre and full-width at half-maximum. The second-order correlation function (*g*^2^(*τ*)) was calculated from the TTTR data with the pycorrelate package using the algorithm by Laurence et al.^67^ and fitted by a biexponential function (shared lifetimes, no constant offset) to predict the *g*^2^(0). The blinking traces were obtained by binning the TTTR data into 1 ms bins, and the fraction of time spent in the ON state was determined by thresholding after visual inspection of intensity histograms and traces (Supplementary Fig. 30).

### Photocatalysis with CsPbBr_3_ NCs

Benzyl bromide (69.5 µl) and a photocatalyst (CsPbBr_3_ NCs, 1.35 mg) were combined with the solvent of choice (1 ml) and N,N-diisopropylethylamine (306 µl) in a 4 ml vial that is then sealed with Parafilm. The vial is placed in the temperature-controlled photoredox device PhotoRedOx TC from HepatoChem (HCK1006-01-025) equipped with a 450 nm blue light-emitting diode (30 W, 250 V) for 4 h at 30 °C. After this time, the reaction mixture was transferred to a round-bottom flask with the help of dichloromethane and all solvent was evaporated. The dry leftover was dissolved in 0.5–0.6 ml of CDCl_3_ for NMR (300 Hz) to analyse the product yield.

## Online content

Any methods, additional references, Nature Portfolio reporting summaries, source data, extended data, supplementary information, acknowledgements, peer review information; details of author contributions and competing interests; and statements of data and code availability are available at 10.1038/s41586-023-06932-6.

### Supplementary information

Supplementary Information Supplementary Notes 1–7, Figs. 1–36, Tables 1–13 and references—see Contents page for details.

## Data Availability

The data that support the findings of this study are available on Zenodo public depository and from the corresponding author on reasonable request.
