# Supplementary material for: Designer phospholipid capping ligands for soft metal halide nanocrystals
Source: Nature. 2023 Dec 18;626(7999):542–8. doi: 10.1038/s41586-023-06932-6 (PMC10866715; doi:10.1038/s41586-023-06932-6)
Supplement: Supplementary file 1 — Supplementary Notes 1–7, Figs. 1–36, Tables 1–13 and references—see Contents page for details. [file 41586_2023_6932_MOESM1_ESM.pdf]

nature portfolio

https://doi.org/10.1038/s41586-023-06932-6

Supplementary information

Designer phospholipid capping ligands for soft metal halide nanocrystals

In the format provided by the authors and unedited

Supplementary Information for

Designer phospholipid capping ligands for soft metal halide nanocrystals

Viktoriia Morad[1,2], Andriy Stelmakh[1,2], Mariia Svyrydenko[1,2], Leon G. Feld[1,2], Simon C. Boehme,[1,2] Marcel Aebli[1,2], Joel Affolter[1], Christoph J. Kaul[1], Nadine J. Schrenker[3], Sara Bals[3], Yesim Sahin,[1,2] Dmitry N. Dirin,[1,2] Ihor Cherniukh[1,2], Gabriele Raino[1,2], Andrij Baumketner[4] & Maksym V. Kovalenko[1,2]*

[1]Department of Chemistry and Applied Biosciences, Institute of Inorganic Chemistry, ETH Zürich,

Zürich, Switzerland

[2]Empa – Swiss Federal Laboratories for Materials Science and Technology, Dübendorf, Switzerland

[3]Electron Microscopy for Materials Science (EMAT) and NANOlab Center of Excellence, University of Antwerp, Antwerp, Belgium

[4]Institute for Condensed Matter Physics, National Academy of Sciences of Ukraine, Lviv, Ukraine

[*]Corresponding author, email: mvkovalenko@ethz.ch

Table of contents

Supplementary Note 1. Slab model of perovskite surfaces ... 4

Supplementary Fig. 1 | Preparation of perovskite surfaces with different stoichiometry. ... 4

Supplementary Note 2. Details of the replica-exchange MD simulations ... 5

Supplementary Fig. 2 | Contribution of flat-bottomed restraints to the potential energy of the system with the surface composition [FABr] = 50%, [PC] = 50% at 300K ... 6

Supplementary Fig. 3. | A set of Br-Br-Br angles ... 6

Supplementary Fig. 4 | Restraints that were used in replica-exchange MD simulations ... 7

Supplementary Fig. 5 | NCs surface termination. ... 7

Supplementary Fig. 6 | Validation of the perovskite model. ... 8

Supplementary Table 1. Geometry of BM3 for PC and PEA ligands on the $FAPbBr_3$ surface. ... 8

Supplementary Fig. 7 | Details of the REDOR experiment ... 9

Supplementary Fig. 8 | $^{31}P^{207}Pb$ REDOR NMR results for NCs capped with oleyl PC and PEA ligands at different magnetic fields. ... 9

Supplementary Note 3. Assessing ligand binding modes on NC surfaces via FTIR spectroscopy and ab-initio molecular dynamics simulations at the DFT level ... 10

Supplementary Fig. 9 | Infrared spectroscopy analysis of the bound and free ligand vibrations ... 13

Supplementary Fig. 10 | Head-group-resolved phonon spectra from ab-initio molecular dynamics (AIMD) simulation of ligand-capped $FAPbBr_3$ NCs. ... 14

Supplementary Fig. 11 | Infrared spectroscopy on PC and PC-capped perovskite NCs can be used to analyze the bound and free ligand vibrations, N-C and C-H regions ... 15

Supplementary Fig. 12 | FTIR spectra of PEA and PEA-d3, aiding in assignment of vibrations related to NH_3^+ headgroup ... 15

Supplementary Table 2. Summary of the relevant FTIR peaks for the PEA ligand. ... 15

Supplementary Table 3. Summary of the relevant FTIR peaks for the PC ligand. ... 16

Materials ... 16

Supplementary Note 4. Ligand synthesis details ... 17

Supplementary Fig. 13 | Selected ^{1}H and ^{31}P NMR spectra of the intermediates and final products in the ligand synthesis. ... 17

Supplementary Fig. 14 | Synthesis of polyzwitterions ... 23

Supplementary Note 5. Nanocrystals synthesis details ... 24

Supplementary Table 4. $FAPbBr_3$ NCs synthesis details ... 24

Supplementary Table 5. $MAPbBr_3$ NCs synthesis details ... 24

Supplementary Table 6. $CsPbBr_3$ NCs synthesis details ... 24

Mixed halide perovskite NCs ... 24

Supplementary Fig. 15 | Optical photographs of phospholipid-capped $CsPbX_3$ NCs (a) and $MAPbX_3$ NCs (b). ... 25

Supplementary Table 7. $CsPbX_3$ NCs synthesis details ... 25

Supplementary Table 8. $FAPbX_3$ NCs synthesis details ... 26

Supplementary Table 9. $MAPbX_3$ NCs synthesis details 27
Lead-free metal halides..... 27
Supplementary Table 10. Sb- and Bi-halide NC synthesis 27
Supplementary Fig. 16 | Ligand exchange experiment for double perovskite Cs_2AgBiX_6 NCs. 28
Supplementary Fig. 17 | Cs-Bi-X and Cs-Sb-X NCs stabilized by PEA ligands. 28
Supplementary Note 6. Purification of NCs 29
Supplementary Fig. 18 | A typical purification procedure following TOPO-DOPA synthesis. 29
Supplementary Fig. 19 | Br-C12-PEA-capped $FAPbBr_3$ NCs..... 30
Supplementary Fig. 20 | Additional details for the PEG-PEA-capped $CsPbBr_3$ NCs. 31
Supplementary Table 12. NCs capped with functionalized PEG tails and their compatibility with various solvents 32
Supplementary Fig. 21 | PEG-PEA-capped $FAPbBr_3$ NCs. 32
Supplementary Fig. 22 | PPG-PEA-capped NCs. 33
Supplementary Fig. 23 | Demonstration of poly(propylene glycol) PEA-capped perovskite NCs dispersibility in propylene glycol methyl ether acetate (PGMEA), a common solvent in semiconductor industry 34
Supplementary Fig. 24 | Interparticle distance tuning by ligands 34
Supplementary Fig. 25 | Stability of PEA-capped $FAPbBr_3$ NCs after multiple purification cycles. 35
Supplementary Fig. 26 | Stability of $MAPbBr_3$ NCs 35
Supplementary Fig. 27 | Humidity test performed on $FAPbBr_3$ NCs films capped with PEA-C8C12 and commercial lecithin ligand..... 36
Supplementary Fig. 28 | Size-tunability of PEA-capped $FAPbBr_3$ NCs. 36
Supplementary Fig. 29 | Optical properties of $FAPbBr_3$ NCs. 37
Supplementary Fig. 30 | Statistics of ON fraction across 78 NCs. 37
Supplementary Fig. 31 | Blinking of single $FAPbBr_3$ NCs capped with C8C12-PEA and lecithin, encapsulated in SEBS and measured under ambient conditions. 38
Supplementary Fig. 32 | Dynamical PL blueshift of single $FAPbBr_3$ NCs capped with C8C12-PEA and lecithin ligands, encapsulated in SEBS and measured under ambient conditions and similar excitation density. 38
Supplementary Fig. 33 | Spectral stability of a single PEA-capped $FAPbBr_3$ NC in SEBS film measured under nitrogen atmosphere 39
Supplementary Fig. 34 | Stability of C16-PC and C16-PEA-capped NCs. 39
Supplementary Fig. 35 | $CsPbBr_3$ and $FAPbBr_3$ NCs capped with a bis-zwitterion. 40
Supplementary Note 7. Direct synthesis of $FAPbBr_3$ NCs in the presence of a zwitterionic ligand.... 40
Supplementary Fig. 36 | 'Direct' hot-injection synthesis of perovskite NCs 42
Supplementary Table 13. Long-term colloidal stability in different solvents..... 42
References..... 43

Supplementary Note 1. Slab model of perovskite surfaces

The surface of perovskite NCs was modelled using a crystal slab with a size of 8 × 8 × 3.5 primitive unit cells. The primitive unit cell was chosen as a basis for crystallographic description to allow direct comparison between different perovskite structures. In the case of room-temperature orthorhombic modification of $CsPbBr_3$[1], it is characterized by two vectors of equal length $a_p = b_p$ with the angle $\gamma = 89.666°$ between them, and another orthogonal vector c_p. Therefore, two nonequivalent (001) and (010) surfaces were considered for $CsPbBr_3$ that correspond to the same (100) surface of cubic $FAPbBr_3$. The simulation box was chosen according to the symmetry of the perovskite crystal, *e.g.,* rectangular box was used for $FAPbBr_3$ and monoclinic box – for $CsPbBr_3$. The simulation box was always kept fixed in XY plane according to the experimental lattice parameters, whereas box length along Z axis was either fixed or allowed to change, depending on the simulated thermodynamic ensemble. A typical value of the box length after equilibration was about 6.5 nm.

Different stoichiometries of the perovskite surfaces were prepared and studied with replica-exchange MD simulations (**Supplementary Fig. 1**). Symmetric PbBr-terminated slab was always used for [ABr] = 0, and ABr-terminated slab for [ABr] = 0.5 and 1.

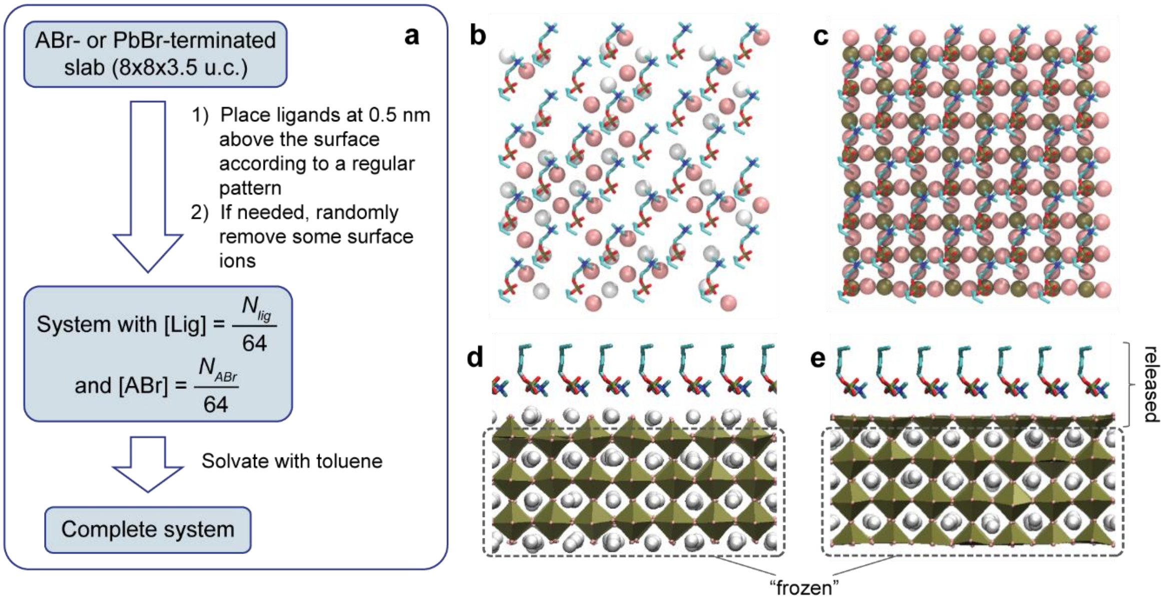

Supplementary Fig. 1 | Preparation of perovskite surfaces with different stoichiometry. a, Steps used to construct input structures. **b,** Example of the surface with [Lig] = 50% and [ABr] = 50% (top view). Note that the initial positions of ligands and vacancies in the surface ABr layer do not match. **c,** Example of the surface with [Lig] = 50% and [ABr] = 0% (top view). In both cases only two surface layers are shown. **d,e,**

Side views of the corresponding slabs. Regions which are kept “frozen” in replica-exchange MD simulations are marked with dashed rectangles.

Supplementary Note 2. Details of the replica-exchange MD simulations

Positions of all ions except the top-most surface layer were restrained with a flat-bottomed harmonic potential (k = 5000 kJ/(mol·nm^2)) to a spherical volume with a radius corresponding to 1/4 of the unit cell size to avoid melting of the entire crystal and to prevent diffusive movement of the ions at high temperatures. The corresponding region of the slab is referred to as “frozen” in **Supplementary Fig. 1**. We note that, despite the name, these restraints have negligible effect on lattice vibrations and distortions at 300 K (**Supplementary Fig. 2**). Movements of the ligands and the surface ions were limited in Z direction by adding repulsive walls at 3 nm above and 0.3 nm below the surface to prevent their migration to the opposite side of the slab.

Our computational setup was found to have an artifact in the case of the orthorhombic $CsPbBr_3$ slab, which is observed as a spontaneous change of its crystallographic orientation. The artifact is caused by relatively small free energy differences between alternative crystallographic orientations of the slab, whereas the corresponding barriers can be easily overcome in replica-exchange MD simulations. To favor the desired orientation, additional harmonic restraints (k = 150 kJ/(mol·rad)) were applied to a set of three orthogonal Br-Br-Br angles (plus three neighboring angles that are parallel to them) formed by distant to the ligand corner-sharing $PbBr_6$ octahedra in the middle layer of the slab (**Supplementary Fig. 3**).

To further limit the configurational space that has to be sampled, a different set of restraints was used for systems containing a single ligand molecule. Firstly, only the minimum number of surface ions were allowed to exchange with the ligand, whereas all the other ions were restrained with the aforementioned flat-bottomed potentials. Second, movements of these ions and the ligand molecule were limited in XY plane in order to keep them close to the place of interest. Details of the restraining scheme are summarized in **Supplementary Fig. 4**. We note that this restraining scheme, while not eliminating any of the physically relevant binding modes completely, introduces some bias into their populations, which needs to be removed by reweighting. Qualitatively, reweighting factors follow the trend BM1 < BM2 = BM2’ < BM3, which means that the actual populations of the BM3 (see **Fig. 2a** and **Extended Data Fig. 2**) should be even closer to unity.

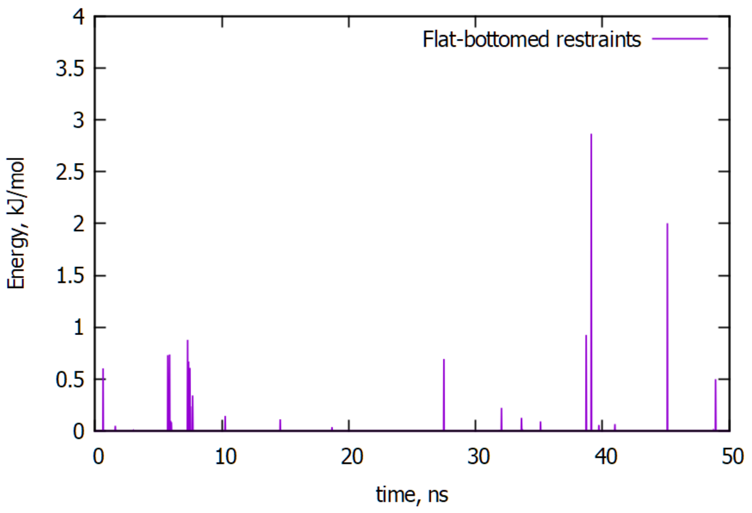

Supplementary Fig. 2 | Contribution of flat-bottomed restraints to the potential energy of the system with the surface composition [FABr] = 50%, [PC] = 50% at 300K. Most of the time restraints are not active.

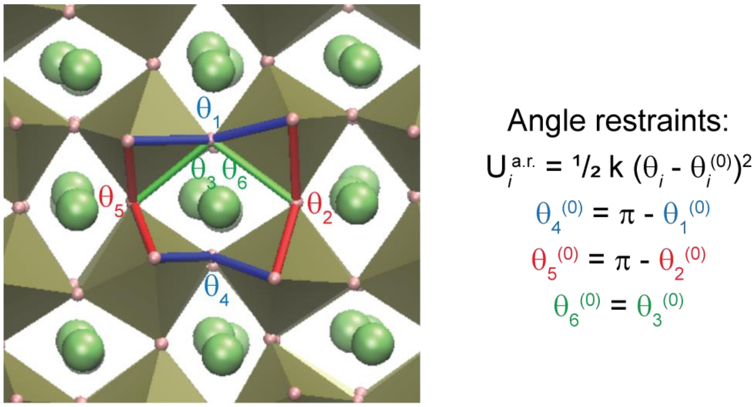

Supplementary Fig. 3. | A set of Br-Br-Br angles, which are restrained to their equilibrium values, to prevent spontaneous change of crystallographic orientation of the $CsPbBr_3$ slab in replica-exchange simulations.

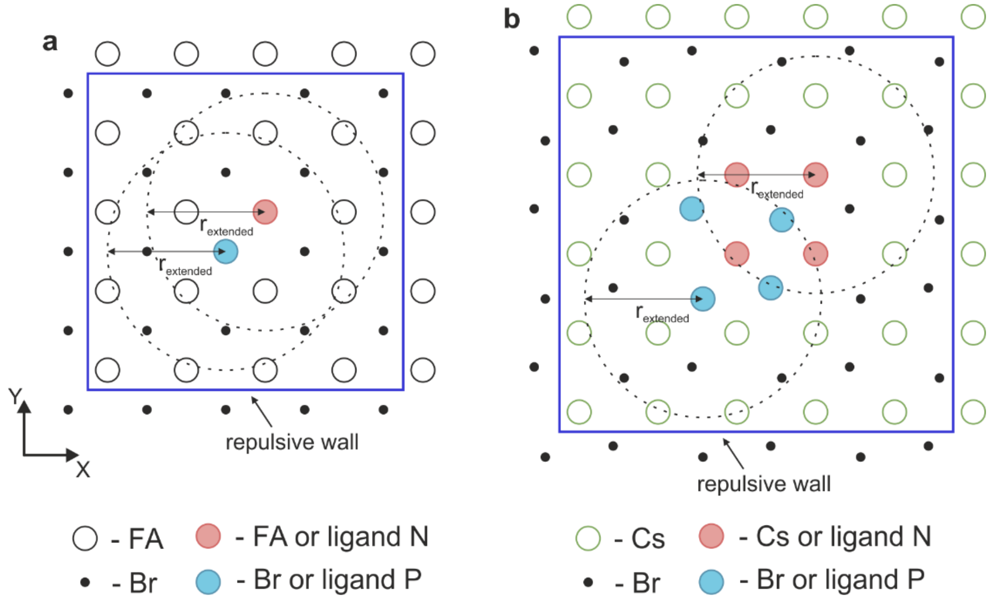

Supplementary Fig. 4 | Restraints that were used in replica-exchange MD simulations of single ligand molecules at the $FAPbBr_3$ (**a**) and $CsPbBr_3$ (**b**) surfaces. Blue and red filled circles denote ions which were allowed to exchange with the ligands. Movements of ions and ligand head-groups (N and P atoms) were limited in XY plane to a square, which is denoted by the blue line. The size of this square was chosen in such way that it accommodates ligand head-group in its fully extended conformation ($r_{extended}$), independent of its attachment point at the surface.

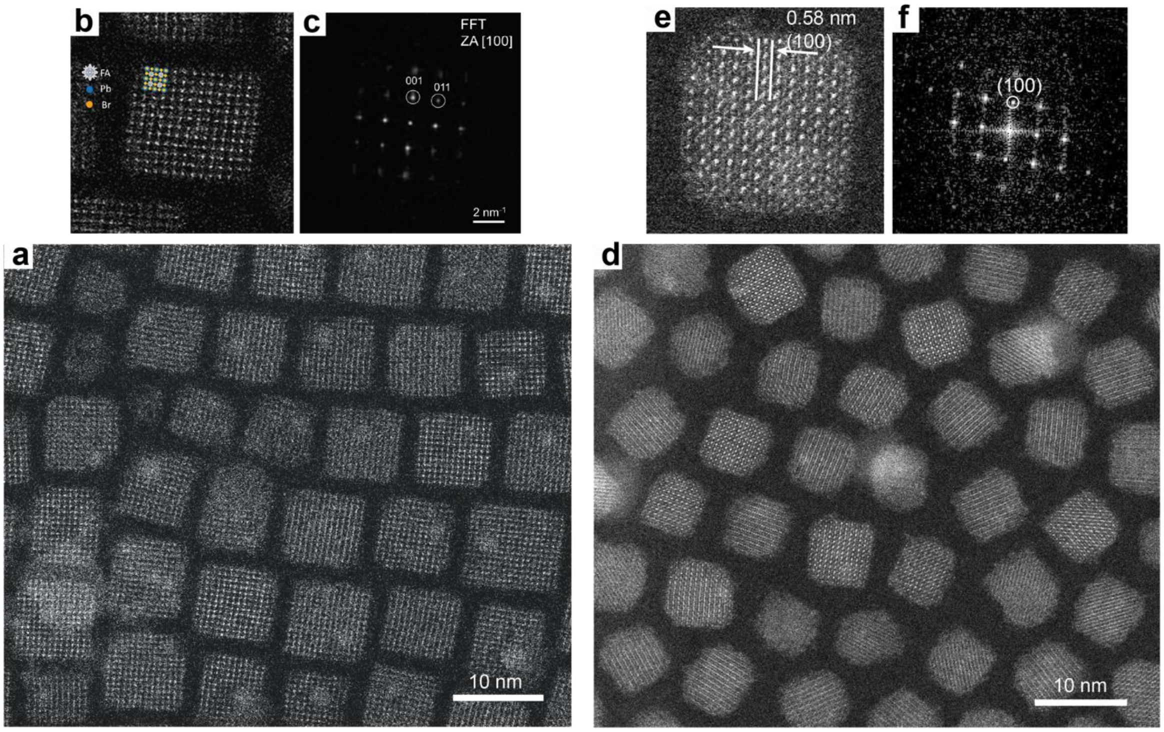

Supplementary Fig. 5 | NCs surface termination. a,d, High resolution HAADF-STEM images of $FAPbBr_3$ (**a**) and $CsPbBr_3$ (**d**) NCs capped with PEA ligands. **b,c,e,f**, (100) lattice spacing of $FAPbBr_3$ (**b**) and $CsPbBr_3$ (**e**) NCs and the corresponding FFTs (**c,f**).

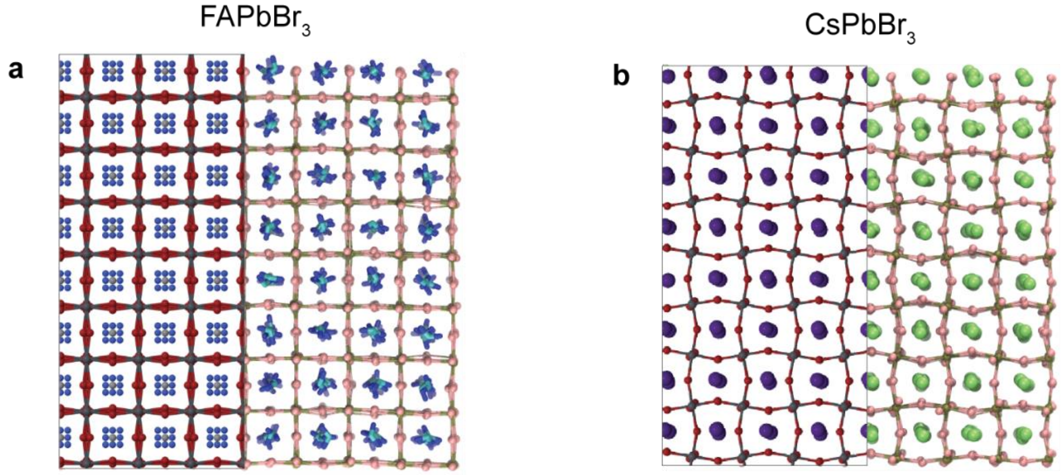

Lattice parameter	Simulation	Experiment[1]	Difference
a, Å	6.045	5.992	0.9%
b, Å	6.045	5.992	0.9%
c, Å	6.045	5.992	0.9%

Lattice parameter	Simulation	Experiment[2]	Difference
a, Å	8.248	8.207	0.5%
b, Å	8.342	8.255	1.0%
c, Å	11.740	11.759	- 0.2%

Supplementary Fig. 6 | Validation of the perovskite model. a, Comparison between the experimentally derived structural model (left) and MD simulation snapshot (right) of $FAPbBr_3$ at 300K. In agreement with the experiment, cubic perovskite structure with disordered FA orientations is observed. **b**, Comparison between the experimentally derived structural model (left) and MD simulation snapshot (right) of $CsPbBr_3$ at 300K. In line with the experiment, orthorhombic perovskite structure is observed. Predicted lattice parameters agree well with the experimental data.[1,2]

Supplementary Table 1. Geometry of BM3 for PC and PEA ligands on the $FAPbBr_3$ surface.

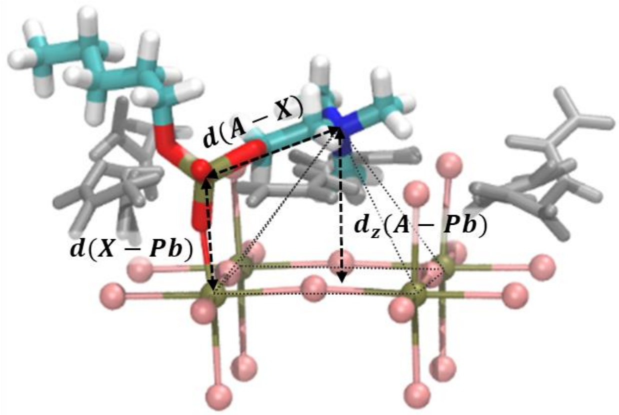

Distances*	PC ligand	PEA ligand	According to crystallographic positions
$\langle d_z(\mathrm{A}-\mathrm{Pb})\rangle$, Å	4.6	2.7	3.0
$\langle d(\mathrm{X}-\mathrm{Pb})\rangle$, Å	3.9	3.9	3.0
$\langle d(\mathrm{A}-\mathrm{X})\rangle$, Å	5.1	4.1	4.2

* “A” denotes the center of a cationic group – nitrogen atom of the PC ligand, center of mass of the $-CH_2NH_3^+$

group in PEA, or crystallographic position of the surface A site, correspondingly. Similarly, “X” denotes the center of an anionic group – phosphorus atom of the PC or PEA ligand, or crystallographic position of the surface X site. Angle brackets indicate trajectory averages, and d_z indicates a projection along the surface normal.

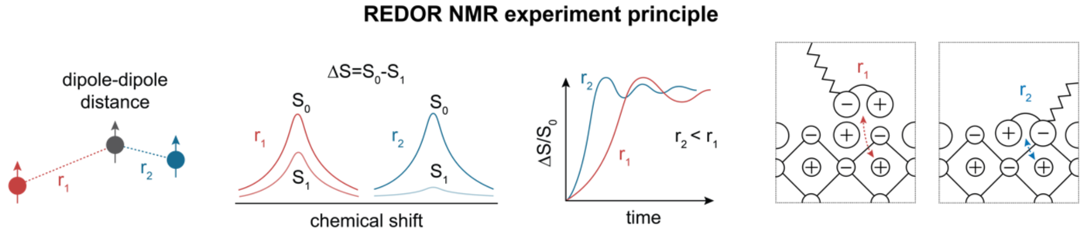

Supplementary Fig. 7 | Details of the REDOR experiment. The dipole-dipole interaction is at the foundation of a REDOR NMR. A shorter dipole-dipole distance induces a faster dipole decay, resulting in a steeper $\Delta S/S_0$ REDOR curve.

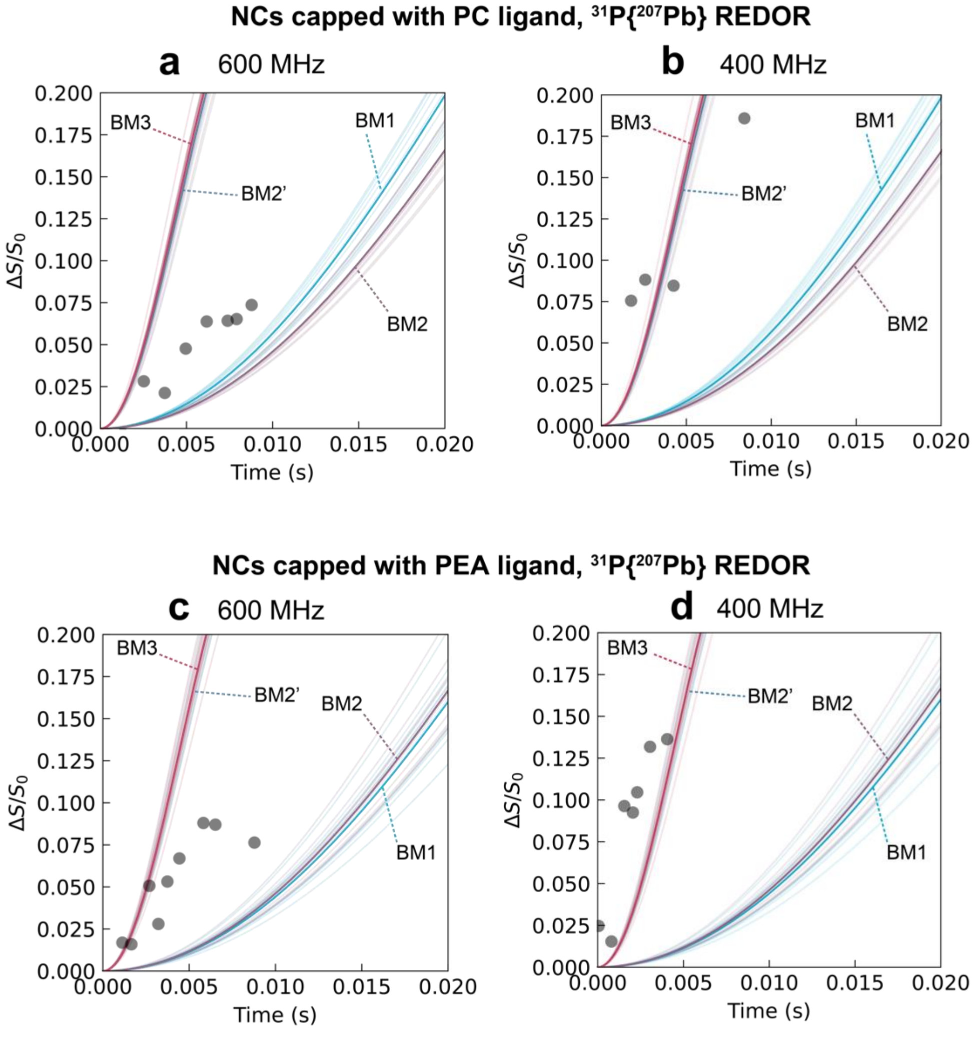

Supplementary Fig. 8 | $^{31}P^{207}Pb$ REDOR NMR results for NCs capped with oleyl PC and PEA ligands

at different magnetic fields. a-b, PC-capped NCs at 600 MHz (a) and 400 MHz (b). **c-d**, PEA-capped NCs at 600 MHz (c) and 400 MHz (d).

Supplementary Note 3. Assessing ligand binding modes on NC surfaces via FTIR spectroscopy and ab-initio molecular dynamics simulations at the DFT level

Ab-initio molecular dynamics simulations at the density-functional level of theory were performed using the CP2K computational package (version 8.2),[3] employing a PBE exchange-correlation functional. Core electrons were described using effective core potentials and outer electrons with a double-ζ basis set augmented with polarization functions (DZVP). Starting from a charge-neutral $FAPbBr_3$ NC with a size of 3x3x3 unit cells and the same surface termination as in our classical molecular dynamics simulation, we add either six PEA or six PC ligands with a density of one ligand per facet (placed in the center of the facet). After an initial geometry optimization and subsequent equilibration of the total energy at 300 K (NVT ensemble with a 1 fs time step and a CSVR thermostat, *i.e.,* canonical sampling via velocity rescaling),[4] we obtain a nuclear trajectory at 300 K of about 3 ps within the NVE ensemble utilizing a 1 fs time step. The latter trajectory is provided to the TRAVIS (Trajectory Analyzer and Visualizer) computational package, which computes the phonon density of states (DOS; power spectrum) via Fourier transformation of the mass-weighted autocorrelation of the nuclear positions along the trajectory. The spectrum of certain vibrations of interest, *e.g.,* those involving the PO_4^- and NH_3^+ ligand functional group, can be extracted via suitable sub-selection of atom groups within the entire structure. While the thus obtained phonon DOS still lacks information on the expected IR intensities, the good agreement of such AIMD-derived mode frequencies with the experimental IR frequencies (typically within ~10 cm^{-1}) increases the accuracy of our vibrational mode assignments, especially in case of spectrally congested regions and/or lacking or widely varying literature reports.

Supplementary Fig. 9a-b discusses major changes in bonding expected when PC and PEA ligands are coordinating to the perovskite surface in BM3. The difference between the two molecules is the ability of PEA headgroup to form close PO…HN contacts through H-bonding due to its strongly donating NH_3^+ group, while the $N(CH_3)_3^+$ headgroup of PC is screened from these interaction.[5] Once the headgroup coordinates to the NC surface, a significant change in H-bonding of the NH_3^+ group is expected.

We analyze and compare experimental FTIR spectra of neat PC and PEA ligands with corresponding spectra of $CsPbBr_3$ and $FAPbBr_3$ NCs capped with these ligands (**Supplementary Fig. 9c-d**). The main region to reveal differences between the neat ligand and NC-surface-bound ligand is between 700 and 1800 cm^{-1}. A prominent change occurs in the P-O stretching region,[6] from about 1050 cm^{-1} to about 1300 cm^{-1}. The neat ligand for both PC (**Supplementary Fig. 9c**) and PEA (**Supplementary Fig. 9d**) features an FTIR absorption peak around 1240 cm^{-1} which can be assigned unambiguously to $\nu_{as}(PO_2)$, *i.e.,* the asymmetric PO_2^- double bond stretches.[6-9] In the case of the PEA ligand (**Supplementary Fig. 9d**), a clear split of $\nu_{as}(PO_2)$ indicates strong hydrogen bonding PO_2^-…H-NH_2^+. The symmetric PO_2^- stretch occurs at 1085-1100 cm^{-1}, but is harder to interpret due to overlap with, amongst others, the C-N stretching (940-

1100 cm^{-1}). The asymmetric PO_2^- stretch is also present in the FTIR spectra of the PC- and PEA-capped $CsPbBr_3$ and $FAPbBr_3$ NCs and it displays a significant bathochromic shift to around 1180 cm^{-1} for PC and 1200 cm^{-1} for PEA. The region towards 1100 cm^{-1} is called 'ionic phosphate vibration' in the literature.[10] A shift towards this region is characteristic of a weakening of P-O bonding due to an interaction with cationic species. The electronegativity of the cationic species is also reflected in the magnitude of the shift: *e.g.,* in silver salt of di-n-butyl phosphate, $\nu_{as}(PO_2)$ vibration occurs at 1152 cm^{-1}, while in the corresponding lead salt, the same peak occurs at 1184 cm^{-1}.[10] Examples even closer to PC and PEA are calcium salts of phosphorylcholine and aminoethylphosphate with $\nu_{as}(PO_2)$ at 1140-1150 cm^{-1}.[11,12] The $\nu_{as}(PO_2)$ shift in PC and PEA capped NCs agrees with literature and can therefore be unambiguously interpreted as dissolution of PO_2^-…H-NH_2^+ hydrogen bonding and phosphate binding to cationic species with subsequent P-O bond weakening and elongation.

To further identify the binding mode and distinguish between the modes BM1, BM2, BM2' and BM3 discussed in the main text, we have performed ab-initio molecular dynamics (AIMD) calculations of the phonon spectra for each of these four cases. **Supplementary Fig. 10** represents the results for PEA ligands placed on a $FAPbBr_3$ NC. There are two major possibilities for the phosphate group to engage in binding on the perovskite NC surface: bound to Pb (in BM3 or BM2', **Supplementary Fig. 10a**) or unbound (BM1 or BM2). In the unbound case, if the positive zwitterionic group (NH_3^+ or $N(CH_3)_3^+$) substitutes the perovskite A-site, the negative phosphate group must be neutralized, likely with an A^+ (FA^+ or Cs^+) cation (BM2, **Supplementary Fig. 10b**). To distinguish phosphate from being either bound to Pb (BM3 or BM2') or unbound (BM2 or BM1), we analyze the PO_4 contribution to the total calculated phonon power spectrum (**Supplementary Fig. 10c**). In both cases of Pb-bound phosphate (BM2' and BM3), we find the contribution from $\nu_{as}(PO_2)$ at around 1180 cm^{-1}, whereas for a FA^+ neutralized unbound phosphate (BM2), this vibrational transition is observed at around 1140 cm^{-1} (and with decreased intensity). The closer agreement of the $\nu_{as}(PO_2)$ peak location for BM3 and BM2' with the corresponding frequencies observed in the experimental FTIR spectrum of PEA-capped $FAPbBr_3$ suggests that PC and PEA engage in either BM2' or BM3, the latter being consistent with the prediction from the replica-exchange MD simulations presented in the main text.

To further distinguish between BM3 and BM2, we further investigate vibrations involving the cationic species of the zwitterion headgroup in PEA and PC, *i.e.,* NH_3^+ and $N(CH_3)_3^+$, respectively. In neat PC, the peaks at 921, 953, and 968 cm^{-1} (**Supplementary Fig. 10c**) can be assigned to vibrations of the -NMe_3 group (920 cm^{-1} and a doublet at around 960 cm^{-1}).[6-9] For PC-capped $CsPbBr_3$ and $FAPbBr_3$ NCs, these peaks show a significant intensity decrease, broadening, and a very small low-frequency shift (**Supplementary Fig. 11a**). Additionally, there is a small bathochromic shift of the CH_3 of the trimethyl ammonium headgroup asymmetric bending modes (δ_{as} (N-CH_3)) in the C-H bend region around 1480 cm^{-1} (**Supplementary Fig. 11b**). The C-H bend region, however, is ambiguous due to contributions from other CH_3 and CH_2 entities of the ligand tail, rendering these changes ill-suited for interpretation.

For the PEA ligands (**Supplementary Fig. 9d**), the FTIR signals at 1560 and 1643 cm^{-1} can be attributed to the symmetric and asymmetric NH_3^+ bending vibrations, $\delta(NH_3)$.[6-9] Additional confirmation for the assignment of the NH_3^+ vibrations is drawn from the comparison to ND_3^+ in PEA-d3 (**Supplementary Fig. 12**). Splitting of the $\delta_{as}(NH_3)$ at 1643 cm^{-1} indicates hydrogen bonding between ammonium and phosphate groups. Upon binding to the NCs, both $\delta_s(NH_3)$ and $\delta_{as}(NH_3)$ show strong bathochromic shifts to around 1500 cm^{-1} and 1600 cm^{-1}, respectively, which is consistent with the major loss of hydrogen bonding that occurs between NH_3 and PO_4 groups in the neat ligand. To interpret these shifts, a useful comparison is the N-H bending from the methylammonium cation, $CH_3NH_3^+$, in bulk $CH_3NH_3PbBr_3$: $\delta_s(NH_3)$ and $\delta_{as}(NH_3)$ appear at 1477 cm^{-1} and 1585 cm^{-1}, respectively,[13] which is rather comparable with the shift found in PEA-capped perovskite NCs. AIMD simulations of the phonon spectra for all binding modes (**Supplementary Fig. 10c**) reveals $\delta_s(NH_3)$ and $\delta_{as}(NH_3)$ signals for BM2 and BM3 (each with the NH_3^+ group substituting the A cation) at the same location as in the experimental spectrum, around 1500 and 1580-1600 cm^{-1}, as expected for BM3.

In summary, the combined FTIR findings on phosphate and ammonium headgroups, corroborated by AIMD simulations and comparison to prior literature, conclude that only BM3 is holds for PEA-capped $FAPbBr_3$ and $CsPbBr_3$ NCs. BM3 is a prevailing binding motif of the PEA-ligand binding also in our computational prediction of the ligand binding (replica-exchange MD simulations, **Fig. 2**) and REDOR NMR data. For the PC ligand, while the phosphate FTIR shifts (and REDOR NMR data) attest the binding of the phosphate to the Pb atoms, we refrain from strong assertions as to the $N(CH_3)_3^+$ group insertion into the A-site owing to spectral overlaps and lack of hydrogen-bonding capability (so instrumental for PEA).

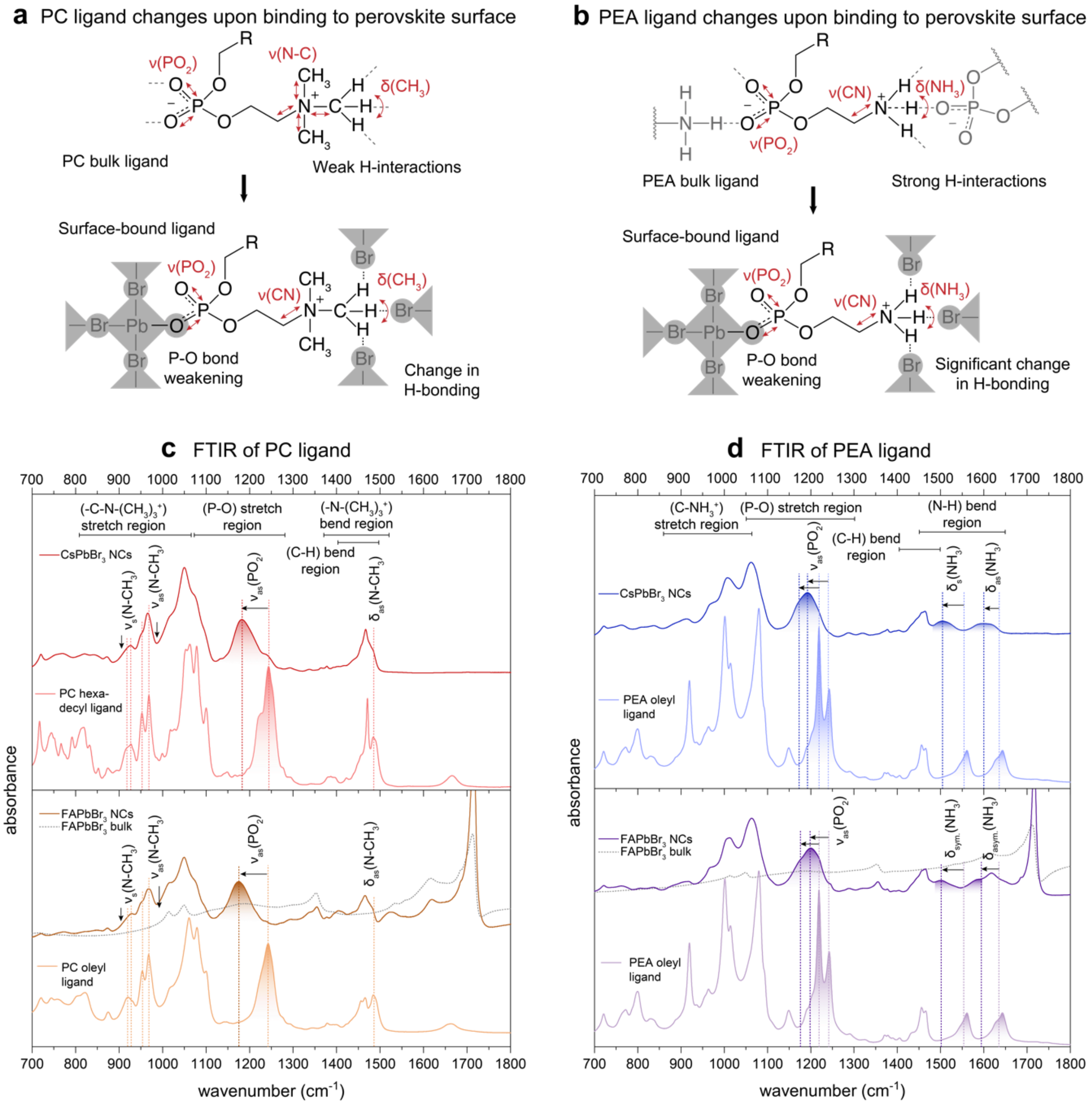

Supplementary Fig. 9 | Infrared spectroscopy analysis of the bound and free ligand vibrations. a-b, Differences between neat PC (a) and PEA (b) ligands and ligands bound to a perovskite surface in BM3. A distinct difference between PC and PEA molecules is a much stronger intermolecular hydrogen bonding in the latter. Upon binding to perovskite, the main change is expected for the PO_4^- group (bound to Pb in BM3) and the NH_3^+ group of PEA (dissolution of a strong NH_3^+-PO_4^- H-bonding). **c-d**, Experimental FTIR spectra of the PC (c) and PEA (d) ligands in the free (neat) form, as well as after binding to $FAPbBr_3$ and $CsPbBr_3$ NCs. Major bathochromic shifts are observed for the PO_4^- group of both PC and PEA (PO_2 symmetric and asymmetric stretch, 1150-1300 cm^{-1}) and the NH_3^+ group of PEA (NH_3 symmetric and asymmetric bend, 1500-1650 cm^{-1}). A minor bathochromic shift is observed for the CH_3 asymmetric bend from N-methyl groups in PC (1475 cm^{-1}). General differences between bulk and NCs surface bound ligands also include peak broadening.

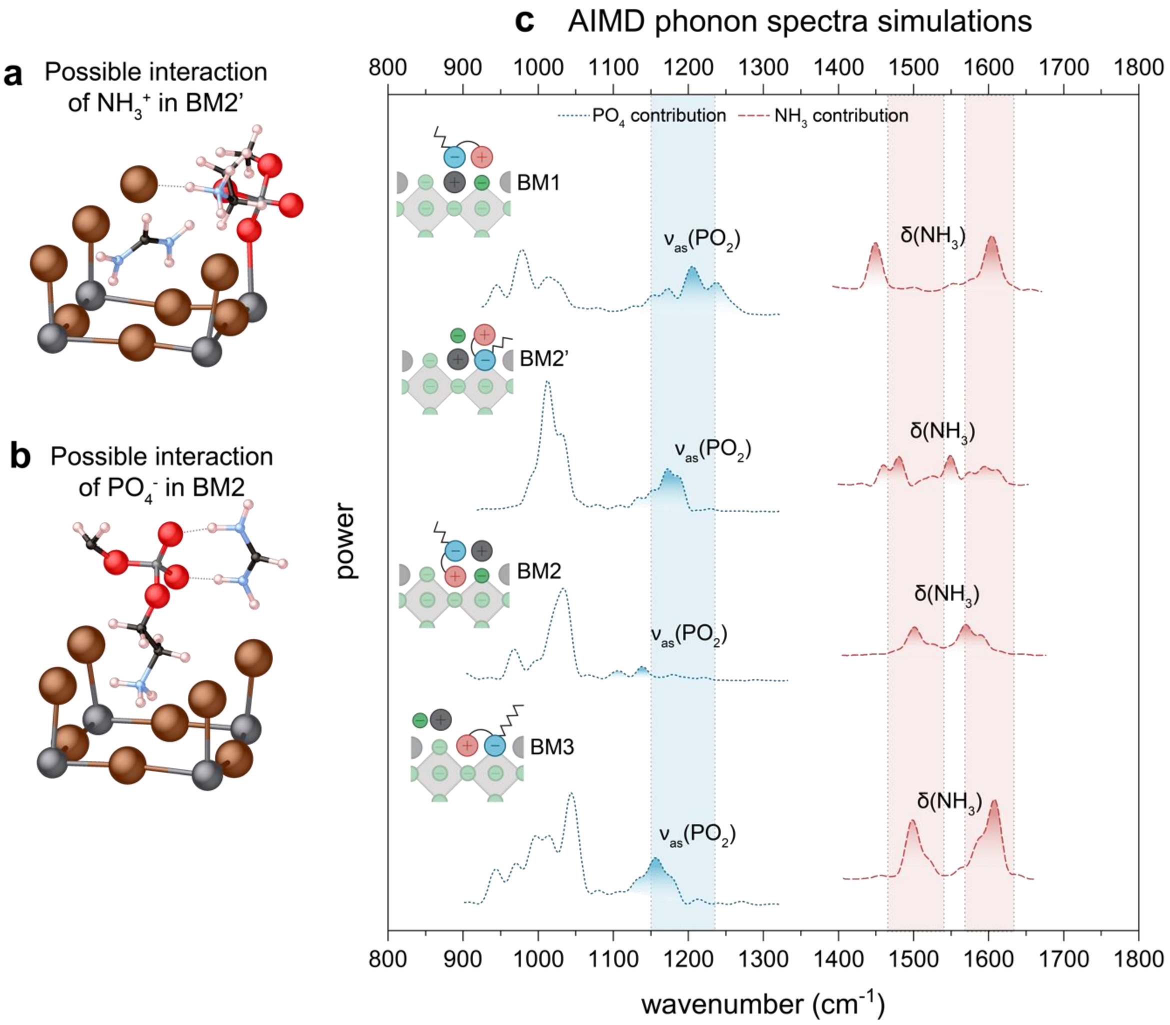

Supplementary Fig. 10 | Head-group-resolved phonon spectra from ab-initio molecular dynamics (AIMD) simulation of ligand-capped $FAPbBr_3$ NCs. a,b, In BM2' and BM2, where one of the head groups of the zwitterion is not bound to the perovskite surface, the positive or negative charge is neutralized by bromide (a) or formamidinium (b) ions, respectively. These interactions cause further bathochromic shift of $\delta(NH_3)$ (BM2') and $\nu_{as}(PO_2)$ (BM2) compared to BM3. **c**, Head-group-resolved phonon spectra obtained from AIMD simulations for various binding modes of a PEA ligand at the $FAPbBr_3$ surface. The range of NH_3^+ bend vibrations ($\delta(NH_3)$, 1450-1650 cm^{-1}) and PO_2 stretch vibrations ($\nu_{as}(PO_2)$, 1140-1200 cm^{-1}) agrees with experimental data in favor of BM3. Shaded area corresponds to experimental peaks from PEA capped $CsPbBr_3$ and $FAPbBr_3$ NCs (Supplementary Fig. 9d, Supplementary Table 2).

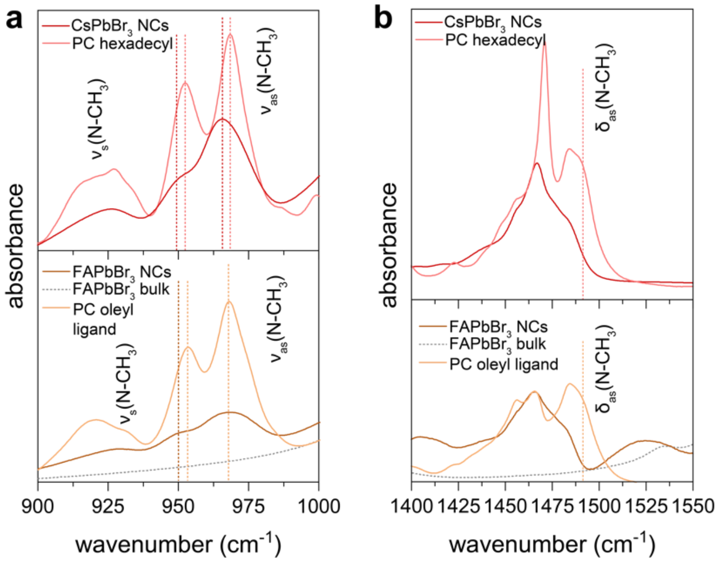

Supplementary Fig. 11 | Infrared spectroscopy on PC and PC-capped perovskite NCs can be used to analyze the bound and free ligand vibrations, N-C and C-H regions. a-b, Close-up of the FTIR regions where $N(CH_3)_3^+$-related vibrations occur.

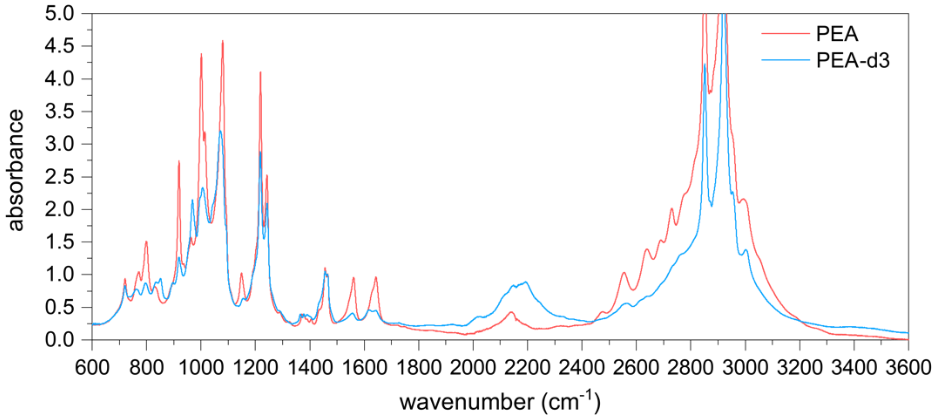

Supplementary Fig. 12 | FTIR spectra of PEA and PEA-d3, aiding in assignment of vibrations related to NH_3^+ headgroup.

Supplementary Table 2. Summary of the relevant FTIR peaks for the PEA ligand.

Vibration mode	AIMD, BM1	AIMD, BM2	AIMD BM2'	AIMD BM3	Experimental, bulk ligand	Experimental, NCs	Other references
$\delta_s(NH_3)$<br>$\delta_{as}(NH_3)$	1450<br>1600	1500<br>1580	1460-1600, multiple peaks due to interaction with Br^-	1500<br>1600	1560<br>1643	$CsPbBr_3$ NCs<br>1505<br>1600<br>$FAPbBr_3$ NCs<br>1499<br>1588	NH_3^+ in $CH_3NH_3PbBr_3$[13]<br>1477<br>1585
$\nu_{as}(PO_2)$	1240	1138	1185	1178	1242	$CsPbBr_3$<br>1191	Ca^{2+} salt of 2-aminoethylphosphate[40]

right peak		$FAPbBr_3$ 1199	1150

Supplementary Table 3. Summary of the relevant FTIR peaks for the PC ligand.

Vibration mode	Experimental, bulk ligand	Experimental, NCs	Other references
$\nu_{as}(PO_2)$ right peak	1244	$CsPbBr_3$ 1181<br>$FAPbBr_3$ 1174	Ca^{2+} salt of phosphorylcholine[39] 1140

Materials

All chemicals for the organic synthesis were used as supplied without further purification. Dry solvents were purified with SPS. *Reagents for ligand synthesis:* phosphorus(V) oxychloride (99%, Sigma-Aldrich); triethylamine (99%, Sigma-Aldrich); 2-aminoethan-1-ol (>=99.0%, Sigma-Aldrich); acetic acid (>99.8%, Sigma-Aldrich); N,N-Bis(2-aminoethyl)ethane-1,2-diamine (97%, ABCR); 1,3-Diaminopropane (98%, ABCR); 4-Diaminobutane (>98.0% (GC)(T), TCI); 1,6-Diaminohexane (puriss., >=99.0% GC/T, Fluka); 2-Chloro-2-oxo-1,3,2-dioxaphospholane (>95.0% (GC)(T), TCI); 1,2-Hexadecanediol (>98.0% (GC), TCI). *Alcohol substrates for ligands synthesis*: 1-Octadecanol (purum, >=95.0% GC, Fluka); 1-Hexadecanol (99%, Aldrich); 1-Tetradecanol (99+%, Acros); 1-Dodecanol (purum, >=95.0% GC, Fluka); cis-9-Octadecen-1-ol (for synthesis, wvr); 2-Octyl-1-dodecanol (97%, Sigma-Aldrich); 10-Phenyl-1-decanol (96%, ABCR); 4-n-Nonylphenol (98+%, Alfa-Aesar); 12-Bromo-dodecan-1-ol (Fluorochem); ω-Hydroxy-terminated polystyrenes (M_n=900 P4465-SOH, M_n=1200 P11121- SOH, M_n=5000 P18731-SOH, Polymer Source, Inc.); Poly(ethylene glycol) monomethyl ethers (M_r=350 ABCR, M_r=750 ABCR, M_r=1000 Aldrich); Poly(propylene glycol) (M_n=725 Sigma-Aldrich); Solutol® HS15 (Poly(ethylene) glycol M_r=660 12-hydoxystearate (BASF); Brij® 58 (Poly(ethylene) glycol M_r=900 monocetyl ether, Fluka); Sorbitan trioleate (Span® 85, Fluka). *Reagents for NC synthesis:* Lead(II) bromide ($PbBr_2$, 99.999%), zinc chloride ($ZnCl_2$, 98%), zinc bromide ($ZnBr_2$, 98%), zinc iodide (ZnI_2, 98%), antimony iodide (SbI_3, 99%), cesium carbonate (Cs_2CO_3, 99.9%), formamidinium acetate (99%), hexane (≥99%), diisooctylphosphinic acid (DOPA, 90%), oleic acid (OA, 90%), trioctylphosphine (TOP, 90%), octadecene (ODE, tech.) and acetone (≥99.5%) were purchased from Sigma Aldrich. n-Octane (min. 99%) was purchased from Carl Roth; trioctylphosphine oxide (TOPO) min. 90% and 99% were purchased from Strem Chemicals; Bromine (Br_2, 99% extra pure) and

mesitylene (99% extra pure) were purchased from Acros. Silver bromide (AgBr, 99%) was purchased from Fluka. Methylamine (MA, 2M in THF), bismuth bromide ($BiBr_3$, 99.999%), bismuth iodide (BiI_3, 99.999%), antimony chloride ($SbCl_3$, 99%) and antimony bromide ($SbBr_3$, 99%) were purchased from ABCR. Commercially purchased ligands: Lecithin for biochemistry (>=97%, Roth AG); 1,2-dioleoyl-sn-glycero-3-phosphocholine (Avanti Lipids); 1,2-dioleoyl-sn-glycero-3-phosphoethanolamine (Avanti Lipids); miltefosine (Christof Senn AG).

Supplementary Note 4. Ligand synthesis details

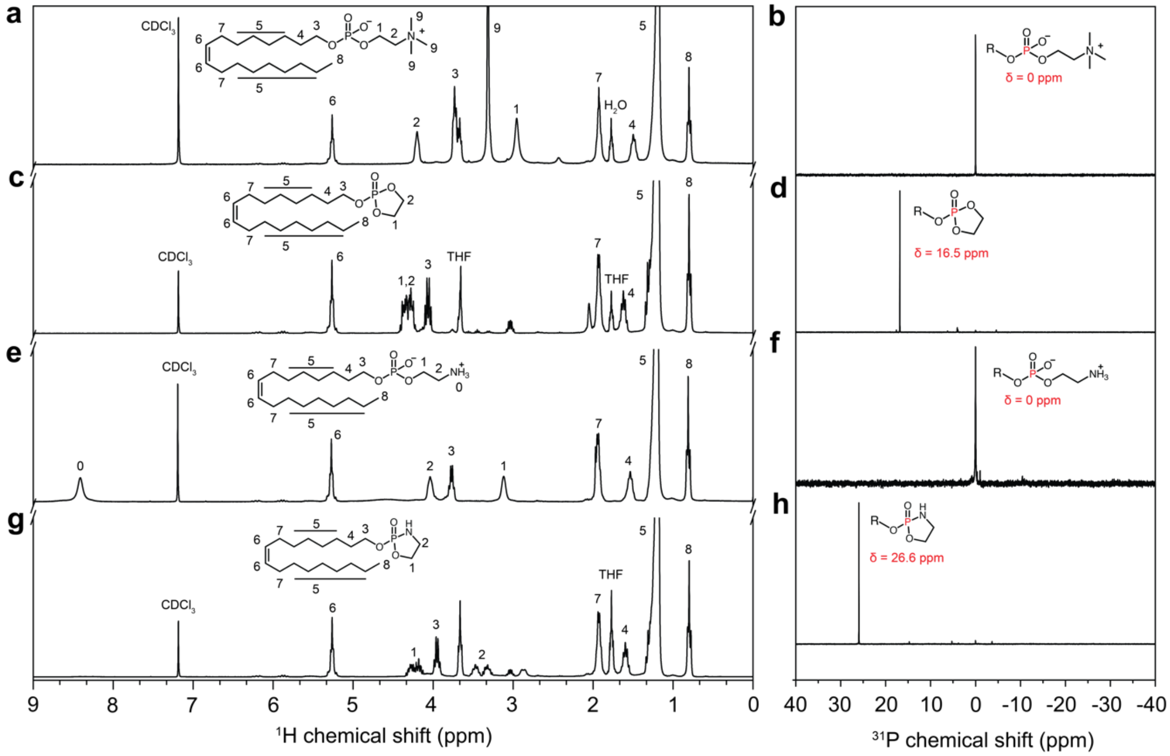

Supplementary Fig. 13 | Selected ^{1}H and ^{31}P NMR spectra of the intermediates and final products in the ligand synthesis.

To isolate ligands soluble in acetone (poly(ethylene) and poly(propylene) glycol-based), after final hydrolysis of oxazaphospholane in water with acetic acid, reaction mixture was diluted with acetone and poured into hexane. Reaction flask was placed in a fridge overnight, during which a product in a form of viscous liquid phase or a solid separated. The product was collected via decantation or centrifugation. For more polar tails, an additional work up sometimes was required: if leftover triethylammonium hydrochloride

signal was present in ^{1}H NMR (t, 3H, 1.3 ppm and q, 2H, 3.2 ppm), final product was dissolved in minimum amount of toluene, leaving triethylammonium hydrochloride as insoluble precipitate. The precipitate was separated through filtration (PTFE syringe filter, 0.22 μm pore size) and the product recovered by removal of toluene in vacuo. This work up was repeated up to 3 times to efficiently remove triethylammonium hydrochloride.

2-Ammonioethyl octadecyl phosphate ***PEA-R1, PEA-C18***. Appearance: white powder, soluble in $CHCl_3$. ^{1}H NMR ($CDCl_3$, ppm) 0.77 (t, 3H, -CH_2-CH_3), 1.24 (m, 26H, [-CH_2-]$_{13}$), 1.51 (t, 2H, -OCH_2-CH_2-), 3.13 (t, 2H, -OCH_2-CH_2-NH_3), 3.78 (t, 2H, -OCH_2-CH_2-), 4.0 (t, 2H, -OCH_2-CH_2-NH_3), 8.2 (s, 3H, -NH_3). ^{31}P NMR ($CDCl_3$, ppm) 0.0 ppm (s, 1P, -PO_4-).

2-Ammonioethyl oleyl phosphate ***PEA-R2, PEA-oleyl***. Appearance: white to yellowish, slightly viscous, hygroscopic, soluble in $CHCl_3$. ^{1}H NMR ($CDCl_3$, ppm) 0.75 (t, 3H, -CH_2-CH_3), 1.2 (m, 20H, [-CH_2-]$_{10}$), 1.50 (t, 2H, -OCH_2-CH_2-), 1.98 (m, 4H, -CH_2-CH=CH-CH_2-), 3.1 (t, 2H, -OCH_2-CH_2-NH_3), 3.8 (t, 2H, -OCH_2-CH_2-), 4.0 (t, 2H, -OCH_2-CH_2-NH_3), 5.26 (m, 2H, -CH=CH-), 8.2 (s, 3H, -NH_3). ^{31}P NMR ($CDCl_3$, ppm) 0.0 ppm (s, 1P, -PO_4-).

2-Ammonioethyl hexadecyl phosphate ***PEA-R3, PEA-C16***. Appearance: white powder, soluble in $CHCl_3$. ^{1}H NMR ($CDCl_3$, ppm) 0.75 (t, 3H, -CH_2-CH_3), 1.25 (m, 22H, [-CH_2-]$_{11}$), 1.50 (t, 2H, -OCH_2-CH_2-), 3.1 (t, 2H, -OCH_2-CH_2-NH_3), 3.77 (t, 2H, -OCH_2-CH_2-), 4.0 (t, 2H, -OCH_2-CH_2-NH_3), 8.3 (s, 3H, -NH_3). ^{31}P NMR ($CDCl_3$, ppm) 0.0 ppm (s, 1P, -PO_4-).

2-Ammonioethyl tetradecyl phosphate ***PEA-R4, PEA-C14***. Appearance: white powder, soluble in $CHCl_3$. ^{1}H NMR ($CDCl_3$, ppm) 0.76 (t, 3H, -CH_2-CH_3), 1.25 (m, 20H, [-CH_2-]$_{10}$), 1.50 (t, 2H, -OCH_2-CH_2-), 3.1 (t, 2H, -OCH_2-CH_2-NH_3), 3.77 (t, 2H, -OCH_2-CH_2-), 4.0 (t, 2H, -OCH_2-CH_2-NH_3), 8.25 (s, 3H, -NH_3). ^{31}P NMR ($CDCl_3$, ppm) 0.0 ppm (s, 1P, -PO_4-).

2-Ammonioethyl dodecyl phosphate ***PEA-R5, PEA-C12***. Appearance: white viscous powder, soluble in $CHCl_3$. ^{1}H NMR ($CDCl_3$, ppm) 0.76 (t, 3H, -CH_2-CH_3), 1.25 (m, 18H, [-CH_2-]$_9$), 1.50 (t, 2H, -OCH_2-CH_2-), 3.1 (t, 2H, -OCH_2-CH_2-NH_3), 3.77 (t, 2H, -OCH_2-CH_2-), 4.0 (t, 2H, -OCH_2-CH_2-NH_3), 8.25 (s, 3H, -NH_3). ^{31}P NMR ($CDCl_3$, ppm) 0.0 ppm (s, 1P, -PO_4-).

2-Ammonioethyl 2-octyl-1-dodecyl phosphate ***PEA-R6, PEA-C8C12***. Appearance: white wax, soluble in $CHCl_3$, toluene, mesitylene. ^{1}H NMR ($CDCl_3$, ppm) 0.79 (t, 6H, -CH_2-CH_3), 1.20 (m, 32H, [-CH_2-]$_9$), 1.49 (m,

1H, -OCH$_2$-*CH*-), 3.16 (m, 2H, -O*CH$_2$*-CH$_2$-NH$_3$), 3.69 (t, 2H, -O*CH$_2$*-CH-), 4.0 (t, 2H, -OCH$_2$-*CH$_2$*-NH$_3$), 8.29 (s, 3H, -*NH$_3$*). ^{31}P NMR (CDCl$_3$, ppm) 0.0 ppm (s, 1P, -PO$_4$-).

2-Ammonioethyl (10-phenyldecyl) phosphate ***PEA-R7, PEA-C10-Ph***. Appearance: white powder, soluble in toluene, CHCl$_3$. ^{1}H NMR (CDCl$_3$, ppm) 0.79 (t, 6H, -CH$_2$-*CH$_3$*), 1.20 (m, 10H, *[-CH$_2$-]$_5$*), 1.51 (t, 2H, -OCH$_2$-*CH*-), 2.5 (t, 2H, -CH$_2$-*Ph*), 3.29 (m, 2H, -O*CH$_2$*-CH$_2$-NH$_3$), 3.89 (t, 2H, -O*CH$_2$*-CH-), 4.2 (t, 2H, -OCH$_2$-*CH$_2$*-NH$_3$), 7.08-7.2 (m, 4H, Ph) 8.0 (s, 3H, -*NH$_3$*). ^{31}P NMR (CDCl$_3$, ppm) 0.0 ppm (s, 1P, -PO$_4$-).

2-Ammonioethyl (4-nonylphenyl) phosphate ***PEA-R8, PEA-Ph-C9***. Appearance: white powder, soluble in toluene, CHCl$_3$. ^{1}H NMR (CDCl$_3$, ppm) 0.79 (t, 3H, -CH$_2$-*CH$_3$*), 1.20 (m, 12H, *[-CH$_2$-]$_6$*), 1.51 (t, 2H, -OPh-CH$_2$-*CH$_2$*-), 2.54 (t, 2H, -Oph-*CH$_2$*-CH$_2$-), 3.29 (m, 2H, -O*CH$_2$*-CH$_2$-NH$_3$), 4.2 (t, 2H, -OCH$_2$-*CH$_2$*-NH$_3$), 7.2 (m, 4H, *Ph*) 8.1 (s, 3H, -*NH$_3$*). ^{31}P NMR (CDCl$_3$, ppm) 0.0 ppm (s, 1P, -PO$_4$-).

2-Ammonioethyl (12-bromododecyl) phosphate ***PEA-R9, PEA-C12-Br***. Appearance: white powder, soluble in CHCl$_3$. ^{1}H NMR (CDCl$_3$, ppm) 1.20 (m, 14H, *[-CH$_2$-]$_7$*), 1.35 (t, 2H, Br-CH$_2$-CH$_2$-*CH$_2$*-), 1.56 (t, 2H, -OCH$_2$-*CH$_2$*-), 1.78 (m, 2H, Br-CH$_2$-*CH$_2$*-CH$_2$-), 3.22 (t, 2H, -O*CH$_2$*-CH$_2$-NH$_3$), 3.35 (t, 2H, Br-*CH$_2$*-CH$_2$-CH$_2$-), 3.83 (t, 2H, -O*CH$_2$*-CH$_2$-), 4.14 (t, 2H, -OCH$_2$-*CH$_2$*-NH$_3$), 8.17 (s, 3H, -*NH$_3$*). ^{31}P NMR (CDCl$_3$, ppm) 0.0 ppm (s, 1P, -PO$_4$-).

2-Ammonioethyl Ω-poly(styrene)-yl phosphate, M_n=900 ***PEA-R10, PEA-PS900.*** Appearance: white viscous, hygroscopic, soluble in CHCl$_3$, toluene. ^{1}H NMR (CDCl$_3$, ppm) 0.5 (m, 9H, *CH$_3$*-CH$_2$-, *CH$_3$*-CH-) 1.37-1.77 (m, nH, *[-CH$_2$-]*), 3.4 (t, 2H, -O*CH$_2$*-CH$_2$-NH$_3$), 3.68 (t, nH, -OCH$_2$-*CH$_2$*-NH$_3$), 6.5-7 (m, nH, Ph) 7.75 (s, 3H, -*NH$_3$*). ^{31}P NMR (CDCl$_3$, ppm) 0.0 ppm (s, 1P, -PO$_4$-).

2-Ammonioethyl Ω-poly(styrene)-yl phosphate, M_n=1200 ***PEA-R11, PEA-PS2300***. Appearance: white viscous, hygroscopic, soluble in CHCl$_3$, toluene. ^{1}H NMR (CDCl$_3$, ppm) 1.37-1.77 (m, nH, *[-CH$_2$-]*), 3.22 (t, 2H, -O*CH$_2$*-CH$_2$-NH$_3$), 3.65 (t, nH, -OCH$_2$-*CH$_2$*-NH$_3$), 6.5-7 (m, nH, Ph) 7.8 (s, 3H, -*NH$_3$*). ^{31}P NMR (CDCl$_3$, ppm) 0.0 ppm (s, 1P, -PO$_4$-).

2-Ammonioethyl Ω-poly(styrene)-yl phosphate, M_n=5000 ***PEA-R12, PEA-PS5000***. Appearance: white viscous, hygroscopic, soluble in CHCl$_3$, toluene. ^{1}H NMR (CDCl$_3$, ppm) 1.37-1.77 (m, nH, *[-CH$_2$-]*), 3.22 (t, 2H, -O*CH$_2$*-CH$_2$-NH$_3$), 3.65 (t, nH, -OCH$_2$-*CH$_2$*-NH$_3$), 6.5-7 (m, nH, Ph) 7.8 (s, 3H, -*NH$_3$*). ^{31}P NMR (CDCl$_3$, ppm) 0.0 ppm (s, 1P, -PO$_4$-).

2-Ammonioethyl (methoxypolyethyleneglycolyl, M_n=350) phosphate ***PEA-R13, PEA-PEG350-OMe***.

Appearance: beige wax, soluble in $CHCl_3$, acetone, insoluble in n-hexane. ^{1}H NMR ($CDCl_3$, ppm) 3.3 (s, 3H, -*OCH$_3$*), 3.6-3.8 (m, [4n+2]H, [-O*CH$_2$*-*CH$_2$*O-]$_n$, -O*CH$_2$*-CH$_2$-), 4.14 (t, 2H, -O*CH$_2$*-CH$_2$-NH$_3$), 4.34 (t, 2H, -OCH$_2$-*CH$_2$*-NH$_3$), 7.77 (s, 3H, -*NH$_3$*). ^{31}P NMR ($CDCl_3$, ppm) 0.0 ppm (s, 1P, -PO$_4$-).

2-Ammonioethyl (methoxypolyethyleneglycolyl, M_n=750) phosphate ***PEA-R14, PEA-PEG750-OMe****.* Appearance: beige wax, soluble in $CHCl_3$, acetone, insoluble in n-hexane. ^{1}H NMR ($CDCl_3$, ppm) 3.3 (s, 3H, -*OCH$_3$*), 3.6-3.8 (m, [4n+2]H, [-O*CH$_2$*-*CH$_2$*O-]$_n$, -O*CH$_2$*-CH$_2$-), 4.14 (t, 2H, -O*CH$_2$*-CH$_2$-NH$_3$), 4.34 (t, 2H, -OCH$_2$-*CH$_2$*-NH$_3$), 7.77 (s, 3H, -*NH$_3$*). ^{31}P NMR ($CDCl_3$, ppm) 0.0 ppm (s, 1P, -PO$_4$-).

2-Ammonioethyl (methoxypolyethyleneglycolyl, M_n=1100) phosphate ***PEA-R15, PEA-PEG1100-OMe****.* Appearance: beige waxy powder, soluble in $CHCl_3$, acetone, insoluble in n-hexane. ^{1}H NMR ($CDCl_3$, ppm) 3.3 (s, 3H, -*OCH$_3$*), 3.6-3.8 (m, [4n+2]H, [-O*CH$_2$*-*CH$_2$*O-]$_n$, -O*CH$_2$*-CH$_2$-), 4.14 (t, 2H, -O*CH$_2$*-CH$_2$-NH$_3$), 4.34 (t, 2H, -OCH$_2$-*CH$_2$*-NH$_3$), 7.77 (s, 3H, -*NH$_3$*). ^{31}P NMR ($CDCl_3$, ppm) 0.0 ppm (s, 1P, -PO$_4$-).

2-Ammonioethyl Brij 58® phosphate ***PEA-R16, PEA-Brij****.* Appearance: white waxy liquid, soluble in $CHCl_3$, acetone. ^{1}H NMR ($CDCl_3$, ppm) 0.8 (t, 3H, -C*H$_3$*), 1.5 (m, 30H, -*OC$_{15}$H$_{30}$*-), 3.5-3.8 (m, [4n+2]H, [-O*CH$_2$*-*CH$_2$*O-]$_n$, -O*CH$_2$*-CH$_2$-), 4.15 (t, 2H, -O*CH$_2$*-CH$_2$-NH$_3$), 4.32 (t, 2H, -OCH$_2$-*CH$_2$*-NH$_3$), 7.75 (s, 3H, -*NH$_3$*). ^{31}P NMR ($CDCl_3$, ppm) 0.0 ppm (s, 1P, -PO$_4$-).

2-Ammonioethyl Triton-X® phosphate ***PEA-R17, PEA-Triton-X****.* Appearance: white honey-like liquid, soluble in $CHCl_3$, acetone, toluene, insoluble in n-hexane. ^{1}H NMR ($CDCl_3$, ppm) 0.6 (s, 15H, -C*H$_3$*), 3.5-3.7 (m, [4n+2]H, [-O*CH$_2$*-*CH$_2$*O-]$_n$, -O*CH$_2$*-CH$_2$-), 4.10 (t, 2H, -O*CH$_2$*-CH$_2$-NH$_3$), 4.28 (t, 2H, -OCH$_2$-*CH$_2$*-NH$_3$), 6.7 (m, 4H, -*Ph*-), 8.1 (s, 3H, -*NH$_3$*). ^{31}P NMR ($CDCl_3$, ppm) 0.0 ppm (s, 1P, -PO$_4$-).

2-Ammonioethyl Solutol® HS 15 phosphate ***PEA-R18, PEA-Solutol****.* Appearance: white honey-like liquid, soluble in $CHCl_3$, acetone, insoluble in n-hexane. ^{1}H NMR ($CDCl_3$, ppm) 0.8 (t, 3H, -C*H$_3$*), 1.5-2.0 (m, 30H, -*OC$_{16}$H$_{31}$*-), 3.5-3.7 (m, [4n+2]H, [-O*CH$_2$*-*CH$_2$*O-]$_n$, -O*CH$_2$*-CH$_2$-), 4.12 (t, 2H, -O*CH$_2$*-CH$_2$-NH$_3$), 4.30 (t, 2H, -OCH$_2$-*CH$_2$*-NH$_3$), 7.9 (s, 3H, -*NH$_3$*). ^{31}P NMR ($CDCl_3$, ppm) 0.0 ppm (s, 1P, -PO$_4$-).

2-Ammonioethyl (hydroxypolypropyleneglycolyl, M_n=725) phosphate ***PEA-R20, PEA-PPG-OH****.* Appearance: yellowish honey-like liquid, soluble in $CHCl_3$, acetone, insoluble in n-hexane. ^{1}H NMR ($CDCl_3$, ppm) 1.07-1.2 (d, 36H, -C*H*$_3$), 3.2-3.6 (m, 36H, -[C*H*$_2$-C*H*]$_{12}$-), 4.12 (t, 2H, -O*CH$_2$*-CH$_2$-NH$_3$), 4.32 (t, 2H, -OCH$_2$-*CH$_2$*-NH$_3$), 8.24 (s, 3H, -*NH$_3$*). ^{31}P NMR ($CDCl_3$, ppm) 0.0 ppm (s, 1P, -PO$_4$-).

2-Ammonioethyl sorbitan trioleate (Span 85®) phosphate ***PEA-R21, PEA-Sorbitan-trioleate****.* Appearance:

yellow honey-like liquid, soluble in $CHCl_3$, acetone, toluene, insoluble in n-hexane. ^{31}P NMR ($CDCl_3$, ppm) 0.0 ppm (s, 1P, -PO_4-).

3-Ammoniopropyl octadecyl phosphate ***PPA-R1, PPA-C18****.* Appearance: white powder, soluble in $CHCl_3$. ^{1}H NMR ($CDCl_3$, ppm) 0.77 (t, 3H, -CH_2-*CH_3*), 1.24 (m, 26H, *[-CH_2-]$_{13}$*), 1.51 (t, 2H, -OCH_2-*CH_2*-), 1.98 (m, 2H, -OCH_2-*CH_2*-CH_2-NH_3), 3.02 (t, 2H, -O*CH_2*-CH_2-CH_2-NH_3), 3.72 (t, 2H, -O*CH_2*-CH_2-), 4.0 (t, 2H, -OCH_2-CH_2-*CH_2*-NH_3), 8.15 (s, 3H, -*NH_3*). ^{31}P NMR ($CDCl_3$, ppm) 0.0 ppm (s, 1P, -PO_4-).

3-Ammoniopropyl oleyl phosphate ***PPA-R2, PPA-oleyl****.* Appearance: yellowish, slightly viscous hygroscopic, soluble $CHCl_3$. ^{1}H NMR ($CDCl_3$, ppm) 0.72 (t, 3H, -CH_2-*CH_3*), 1.24 (m, 20H, *[-CH_2-]$_{10}$*), 1.50 (t, 2H, -OCH_2-*CH_2*-), 1.96 (m, 2H, -OCH_2-*CH_2*-CH_2-NH_3), 1.98 (m, 4H, -*CH_2*-CH=CH-*CH_2*-), 3.02 (t, 2H, -O*CH_2*-CH_2-CH_2-NH_3), 3.74 (t, 2H, -O*CH_2*-CH_2-), 4.0 (t, 2H, -OCH_2-CH_2-*CH_2*-NH_3), 5.26 (m, 2H, -*CH=CH*-), 8.12 (s, 3H, -*NH_3*). ^{31}P NMR ($CDCl_3$, ppm) 0.0 ppm (s, 1P, -PO_4-).

3-Ammoniopropyl hexadecyl phosphate ***PPA-R3, PPA-C16****.* Appearance: white powder, soluble in $CHCl_3$. ^{1}H NMR ($CDCl_3$, ppm) 0.77 (t, 3H, -CH_2-*CH_3*), 1.23 (m, 22H, *[-CH_2-]$_{11}$*), 1.50 (t, 2H, -OCH_2-*CH_2*-), 1.96 (m, 2H, -OCH_2-*CH_2*-CH_2-NH_3), 3.0 (t, 2H, -O*CH_2*-CH_2-CH_2-NH_3), 3.75 (t, 2H, -O*CH_2*-CH_2-), 4.0 (t, 2H, -OCH_2-CH_2-*CH_2*-NH_3), 8.1 (s, 3H, -*NH_3*). ^{31}P NMR ($CDCl_3$, ppm) 0.0 ppm (s, 1P, -PO_4-).

3-Ammoniopropyl (2-octyl-1-dodecyl) phosphate ***PPA-R6, PPA-C8C12****.* Appearance: white wax, slightly soluble in $CHCl_3$, toluene, mesitylene. ^{1}H NMR ($CDCl_3$, ppm) 0.81 (t, 6H, -CH_2-*CH_3*), 1.20 (m, 32H, *[-CH_2-]$_9$*), 1.51 (m, 2H, -OCH_2-*CH*-), 1.96 (m, 2H, -OCH_2-*CH_2*-CH_2-NH_3), 3.06 (m, 2H, -O*CH_2*-CH_2-CH_2-NH_3), 3.70 (t, 2H, -O*CH_2*-CH-), 4.0 (t, 2H, -OCH_2-CH_2-*CH_2*-NH_3), 8.0 (s, 3H, -*NH_3*). ^{31}P NMR ($CDCl_3$, ppm) 0.0 ppm (s, 1P, -PO_4-).

3-Ammoniopropyl (10-phenyldecyl) phosphate ***PPA-R7, PPA-C10-Ph****.* Appearance: white powder, soluble in toluene, $CHCl_3$. ^{1}H NMR ($CDCl_3$, ppm) 0.79 (t, 6H, -CH_2-*CH_3*), 1.20 (m, 10H, *[-CH_2-]$_5$*), 1.51 (t, 2H, -OCH_2-*CH*-), 1.96 (m, 2H, -OCH_2-*CH_2*-CH_2-NH_3), 2.5 (t, 2H, -CH_2-*Ph*), 3.29 (m, 12, -O*CH_2*-CH_2-CH_2-NH_3), 3.89 (t, 2H, -O*CH_2*-CH-), 4.2 (t, 2H, -OCH_2-CH_2-*CH_2*-NH_3), 7.08-7.2 (m, 4H, Ph), 8.0 (s, 3H, -*NH_3*). ^{31}P NMR ($CDCl_3$, ppm) 0.0 ppm (s, 1P, -PO_4-).

4-Ammoniobutyl (2-octyl-1-dodecyl) phosphate ***PBA-R6, PBA-C8C12****.* Appearance: white wax, soluble in $CHCl_3$, toluene, mesitylene. ^{1}H NMR ($CDCl_3$, ppm) 0.79 (t, 6H, -CH_2-*CH_3*), 1.18 (m, 32H, *[-CH_2-]$_9$*), 1.48 (m, 2H, -OCH_2-*CH*-), 1.73 (m, 2H, -OCH_2-*CH_2*-CH_2-CH_2-NH_3), 1.86 (m, 2H, -OCH_2-CH_2-*CH_2*-CH_2-NH_3) 3.0 (m,

2H, -OCH_2-CH_2-CH_2-CH_2-NH_3), 3.74 (t, 2H, -OCH_2-CH-), 3.94 (t, 2H, -OCH_2-CH_2-CH_2-CH_2-NH_3), 8.22 (s, 3H, -NH_3). ^{31}P NMR ($CDCl_3$, ppm) 0.5 ppm (s, 1P, -PO_4-).

*(2-(Trimethylammonio)ethyl) octadecyl phosphate **PC-R1, PC-C18**.* Appearance: white powder, soluble in $CHCl_3$. ^{1}H NMR ($CDCl_3$, ppm) 0.75 (t, 3H, -CH_2-CH_3), 1.22 (m, 26H, [-CH_2-]$_{13}$), 1.51 (t, 2H, -OCH_2-CH_2-), 3.33 (s, 9H, -N$(CH_3)_3$), 3.7 (t, 2H, -OCH_2-CH_2-N$(CH_3)_3$), 3.8 (t, 2H, -OCH_2-CH_2-), 4.25 (t, 2H, -OCH_2-CH_2-N$(CH_3)_3$). ^{31}P NMR ($CDCl_3$, ppm) 0.0 ppm (s, 1P, -PO_4-).

*(2-(Trimethylammonio)ethyl) oleyl phosphate **PC-R2, PC-oleyl**.* Appearance: white to yellowish, slightly viscous, very hygroscopic, soluble $CHCl_3$. ^{1}H NMR ($CDCl_3$, ppm) 0.79 (t, 3H, -CH_2-CH_3), 1.21 (m, 20H, [-CH_2-]$_{10}$), 1.52 (t, 2H, -OCH_2-CH_2-), 1.91 (m, 4H, -CH_2-CH=CH-CH_2-), 3.31 (s, 9H, -N$(CH_3)_3$), 3.75 (t, 2H, -OCH_2-CH_2-N$(CH_3)_3$), 3.77 (t, 2H, -OCH_2-CH_2-), 4.25 (t, 2H, -OCH_2-CH_2-N$(CH_3)_3$), 5.27 (m, 2H, -*CH*=*CH*-). ^{31}P NMR ($CDCl_3$, ppm) 0.0 ppm (s, 1P, -PO_4-).

*(2-(Trimethylammonio)ethyl) 2-octyl-1-dodecyl **PC-R6, PC-C8C12**.* Appearance: white wax, soluble in $CHCl_3$, toluene, mesitylene, very hygroscopic. ^{1}H NMR ($CDCl_3$, ppm) 0.8 (t, 6H, -CH_2-CH_3), 1.18 (m, 32H, [-CH_2-]$_9$), 1.45 (m, 2H, -OCH_2-*CH*-), 3.33 (s, 9H, -N$(CH_3)_3$), 3.62 (m, 1H, -OCH_2-CH_2-N$(CH_3)_3$), 3.75 (t, 2H, -OCH_2-CH-), 4.23 (t, 2H, -OCH_2-CH_2-N$(CH_3)_3$). ^{31}P NMR ($CDCl_3$, ppm) 0.0 ppm (s, 1P, -PO_4-).

*(Propane-1,3-diylbis(ammoniumdiyl))bis(ethane-2,1-diyl) bis(2-octyldodecyl) bis(phosphate) **R6-PEA-propane-PEA-R6, C8C12-PEA-propane-PEA-C8C12**.* Appearance: yellowish viscous liquid, soluble in $CHCl_3$, toluene, mesitylene. ^{1}H NMR ($CDCl_3$, ppm) 0.72 (t, 12H, -CH_2-CH_3), 1.12 (m, 64H, [-CH_2-]$_9$), 1.39 (m, 2H, -OCH_2-*CH*-), 2.0 (m, 2H, -CH_2-NH_2-CH_2-CH_2-) 3.00 (m, 8H, -OCH_2-CH_2-NH_2-CH_2-) 3.62 (m, 4H, -OCH_2-CH_2-NH_2-), 3.75 (t, 4H, -OCH_2-CH-). ^{31}P NMR ($CDCl_3$, ppm) 0.0 ppm (s, 2P, -PO_4-).

*(Butane-1,4-diylbis(ammoniumdiyl))bis(ethane-2,1-diyl) bis(2-octyldodecyl) bis(phosphate) **R6-PEA-butane-PEA-R6, C8C12-PEA-butane-PEA-C8C12**.* Appearance: yellowish viscous liquid, soluble in $CHCl_3$, toluene, mesitylene. ^{1}H NMR ($CDCl_3$, ppm) 0.79 (t, 12H, -CH_2-CH_3), 1.17 (m, 64H, [-CH_2-]$_9$), 1.47 (m, 2H, -OCH_2-*CH*-), 1.81 (m, 4H, -NH_2-CH_2-CH_2-CH_2-CH_2-NH_2-) 3.00 (m, 8H, -OCH_2-CH_2-NH_2-CH_2-) 3.65 (m, 4H, -OCH_2-CH_2-NH_2-), 3.9-4.1 (t, 4H, -OCH_2-CH-). ^{31}P NMR ($CDCl_3$, ppm) 0.0 ppm (s, 2P, -PO_4-).

*(Hexane-1,6-diylbis(ammoniumdiyl))bis(ethane-2,1-diyl) bis(2-octyldodecyl) bis(phosphate) **R6-PEA-hexane-PEA-R6, C8C12-PEA-hexane-PEA-C8C12**.* Appearance: yellowish viscous liquid, soluble in $CHCl_3$, toluene, mesitylene. ^{1}H NMR ($CDCl_3$, ppm) 0.79 (t, 12H, -CH_2-CH_3), 1.17 (m, 64H, [-CH_2-]$_9$), 1.35

(m, 4H, -NH_2-CH_2-CH_2-*CH_2*-*CH_2*-CH_2-CH_2-NH_2-) 1.47 (m, 2H, -OCH_2-*CH*-), 1.64 (m, 4H, -NH_2-CH_2-*CH_2*-CH_2-CH_2-*CH_2*-CH_2-NH_2-) 2.7-2.9 (m, 8H, -OCH_2-*CH_2*-NH_2-*CH_2*-) 3.63 (m, 4H, -O*CH_2*-CH_2-NH_2-), 4.0 (t, 4H, -O*CH_2*-CH-). ^{31}P NMR ($CDCl_3$, ppm) 0.0 ppm (s, 2P, -PO_4-).

Synthetic procedures towards polyzwitterions

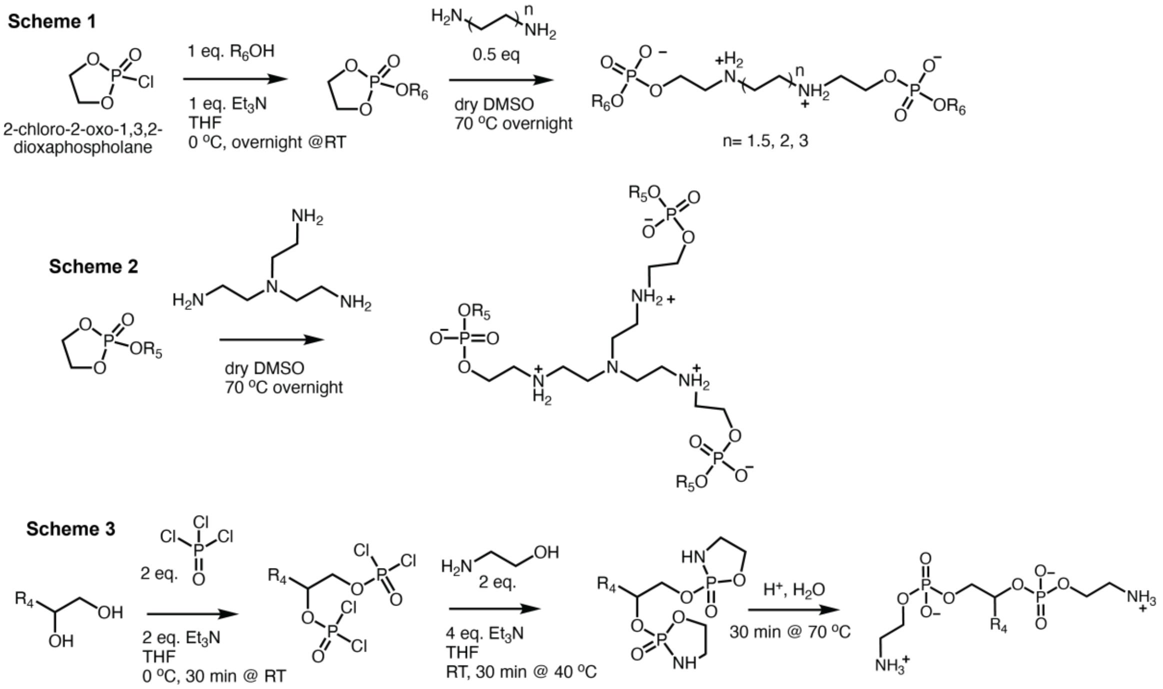

Supplementary Fig. 14 | Synthesis of polyzwitterions. Multiple zwitterions can potentially improve binding to the NC surface (**Supplementary fig. 19**). N-substituted polyzwitterions can be obtained through ring opening of 2-oxoalkyl-2-oxo-1,3,2-dioxaphospholane with various amines (**Scheme 1** and **2**). Large library of such molecules can be found in Ref.[14]. Another method is using diols as substrates in reaction with phosphorous(V) oxychloride (**Scheme 3**).

(Tris(2-aminoethyl)-2,2',2''-triyltris(ammoniumdiyl)) trisdodecyl tris(phosphate) ***(R5-PEA-ethane-)$_3$N, (C12-PEA-ethane-)$_3$N***. Appearance: yellow viscous liquid, soluble in $CHCl_3$. ^{1}H NMR ($CDCl_3$, ppm) 0.8 (t, 9H, -CH_2-*CH_3*), 1.2 (m, 48H, *[-CH_2-]*), 1.53 (m, 6H, -OCH_2-*CH_2*-), 2.7 (m, 8H, -OCH_2-*CH_2*-NH_2-*CH_2*-) 3.57 (t, 6H, -NH_2-CH_2-*CH_2*-N-), 3.65 (t, 6H, -NH_2-*CH_2*-CH_2-N-), 3.78 (m, 6H, -O*CH_2*-CH_2-NH_2-CH_2-), 3.87-4.0 (m, 6H, -O*CH_2*-CH-), 7.8 (s, 6H, -*NH_3*). ^{31}P NMR ($CDCl_3$, ppm) 0.01 ppm (s, 3P, -PO_4-).

Bis(2-ammonioethyl) hexadecane-1,2-diyl bis(phosphate) ***R4-ethane(PEA)$_2$, C14-ethane(PEA)$_2$.*** Appearance: yellowish powder, soluble in $CHCl_3$. ^{1}H NMR ($CDCl_3$, ppm) 0.79 (t, 3H, -CH_2-*CH_3*), 1.20 (m, 20H, *[-CH_2-]*), 1.48 (m, 2H, -OCH_2-*CH_2*-), 3.3 (m, 4H, -O*CH_2*-CH_2-NH_3), 3.75 (m, 2H, -O*CH_2*-CH_2-), 4.26 (t,

4H, -OCH_2-CH_2-NH_3), 8.42 (s, 6H, -NH_3). ^{31}P NMR ($CDCl_3$, ppm) 0.0 ppm (s, 2P, -PO_4-).

Supplementary Note 5. Nanocrystals synthesis details

TOPO-DOPA-based synthesis (see **Methods**), at room temperature, allows for the separation of nucleation and growth, as well as results in NCs capped with poor ligands (TOPO and DOPA; NCs cannot be purified and isolated without adding a good ligand afterwards), which can be efficiently replaced with the phospholipid of choice.

Supplementary Table 4. $FAPbBr_3$ NCs synthesis details

Pb precursor [uL]	FA precursor [uL]	n-hexane	Reaction time	Ligand	NC size
260	100	3 ml	30 s	12 umol	10 nm
260	100	15 ml	15 s	12 umol	7 nm
260	100	30 ml	15 s	12 umol	5 nm

Supplementary Table 5. $MAPbBr_3$ NCs synthesis details

Pb precursor [uL]	MA precursor [uL]	n-hexane	Reaction time	Ligand	NC size
260	86	3 ml	<10 s	12 umol	10 nm
260	86	30 ml	<10 s	12 umol	8 nm

Supplementary Table 6. $CsPbBr_3$ NCs synthesis details

Pb precursor [uL]	Cs precursor [uL]	n-hexane	Reaction time	Ligand	NC size
260	250	500 uL	90 s	6 umol	8 nm
260	250	1000 uL	60 s	6 umol	6 nm

Mixed halide perovskite NCs. TOPO-DOPA synthesis can be modified to produce Cl-Br and Br-I mixed halide $CsPbX_3$, $FAPbX_3$ and $MAPbX_3$ NCs. For the iodides, AOA precursor instead of ADOPA is used. *CsOA stock solution* was prepared by dissolving Cs_2CO_3 (78.2 mg, 0.24 mmol) and OA (4 ml, 12.67 mmol) in 20 ml of n-hexane at RT in air. *FAOA stock solution* was prepared by dissolving formamidinium acetate (51.3 mg) with OA (4 ml) in n-hexane (20 ml) in air at RT. Analogously, MAOA stock solution was prepared by mixing 2M MA in THF (120 uL) with OA (1 ml) in n-hexane (5 ml) at RT. Zinc halide TOPO complexes were used as halide sources in addition to $PbBr_2$-TOPO. For the mixed halide NCs, 99% TOPO instead of

90% was used to prepare metal halide precursor. For $CsPb(Br/I)_3$, in addition to ZnI_2, also $ZnBr_2$-TOPO was used to keep the same TOPO-halide ratio throughout the synthesis. *ZnX_2 stock solutions* were prepared by dissolving ZnX_2 (2.04 mmol) and TOPO (4296 mg, 11.11 mmol) in 25 ml n-octane at 120 ºC on a hotplate in air.

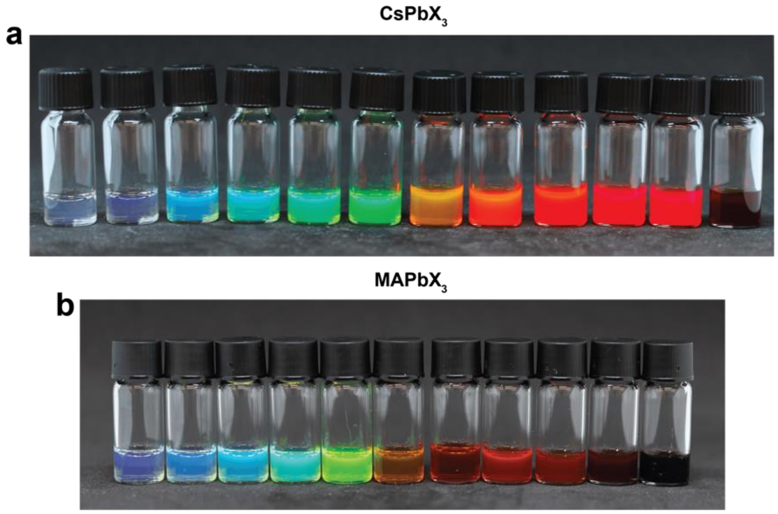

Supplementary Fig. 15 **| Optical photographs of phospholipid-capped $CsPbX_3$ NCs (a) and $MAPbX_3$ NCs (b).**

Supplementary Table 7. $CsPbX_3$ NCs synthesis details

Pb precursor [uL]	Halide precursor [uL]	Cs precursor [uL]	n-hexane [ml]	Reaction time	Ligand used, mass (mg)	PL [nm]	FWHM [nm]	PLQY [%]
$PbBr_2$ 260	$ZnCl_2$ 400	CsDOPA 300	1	300 s	C8C12PEA 2 mg	432	11	54
$PbBr_2$ 260	$ZnCl_2$ 200	CsDOPA 300	1	300 s	C8C12PEA 2 mg	450	17	63
$PbBr_2$ 260	$ZnCl_2$ 100	CsDOPA 300	1	300 s	C8C12PEA 2 mg	467	18	64
$PbBr_2$ 260	$ZnCl_2$ 60	CsDOPA 300	1	300 s	C8C12PEA 2 mg	478	17	74

$PbBr_2$ 260	$ZnCl_2$ 20	CsDOPA 300	1	300 s	C8C12PEA 2 mg	493	19	85
$PbBr_2$ 100	ZnI_2 100 $ZnBr_2$ 150	CsOA 300	4	60 s	C8C12PEA 2 mg	522	23	92
$PbBr_2$ 100	ZnI_2 150 $ZnBr_2$ 100	CsOA 300	4	40 s	C8C12PPA 3.5 mg	561	28	74
$PbBr_2$ 100	ZnI_2 175 $ZnBr_2$ 75	CsOA 300	4	40 s	C8C12PPA 3.5 mg	578	30	70
$PbBr_2$ 100	ZnI_2 200 $ZnBr_2$ 50	CsOA 300	4	40 s	C8C12PPA 3.5 mg	600	34	80
$PbBr_2$ 100	ZnI_2 225 $ZnBr_2$ 25	CsOA 300	4	40 s	C8C12PPA 3.5 mg	630	36	82
$PbBr_2$ 100	ZnI_2 250	CsOA 300	4	40 s	C8C12PPA 3.5 mg	650	33	63

Supplementary Table 8. $FAPbX_3$ NCs synthesis details

Pb precursor [uL]	Halide precursor [uL]	FA precursor [uL]	n-hexane [ml]	Reaction time	PL [nm]	PLQY [%]
$PbBr_2$ 200	$ZnCl_2$ 85.7	600	5	2 s	488	100
$PbBr_2$ 200	$ZnCl_2$ 200	600	5	2 s	470	87
$PbBr_2$ 200	$ZnCl_2$ 466	600	5	2 s	449	27
$PbBr_2$ 200	ZnI_2 200	600	0.9	2 s	651	24
$PbBr_2$ 200	ZnI_2 250	600	0.9	2 s	673	26
$PbBr_2$ 200	ZnI_2 300	600	0.9	2 s	712	28

Supplementary Table 9. $MAPbX_3$ NCs synthesis details

Pb precursor [uL]	Halide precursor [uL]	MA precursor [uL]	n-hexane [ml]	Reaction time	PL [nm]	PLQY [%]
$PbBr_2$ 145	$ZnCl_2$ 16.11	700	5	2 s	487	98
$PbBr_2$ 145	$ZnCl_2$ 62.1	700	5	2 s	475	91
$PbBr_2$ 145	$ZnCl_2$ 145	700	5	2 s	456	27

Lead-free metal halides. $Cs_2AgBiBr_6$ NCs were prepared according to procedure reported elsewhere.[15] Afterwards, PEA-C8C12 (5 mg) in chloroform was added to the dispersion of NCs in n-hexane. NCs were subsequently purified up to 5 times with a mixture of 2:1 ethyl acetate:acetonitrile to remove OAm. Cs_3BiBr_6, $Cs_3Bi_2Br_9$, $Cs_3Bi_2I_9$, $Cs_3Sb_2Cl_9$, $Cs_3Sb_2Br_9$ and $Cs_3Sb_2I_9$ NCs were prepared by dissolving the corresponding metal halide and TOPO in n-octane in the same ratio and concentrations as PbX_2 above (0.067M). CsOA (0.02M) was used as a source of Cs. The reaction wash quenched with 2 mg of PEA-C8C12 and NCs were purified up to 3 times.

Supplementary Table 10. Sb- and Bi-halide NC synthesis

M precursor [uL]	Cs precursor [uL]	n-hexane [ml]	Reaction time	Product
$BiBr_3$ 100	200	0.25	2 s	$Cs_3Bi_2Br_9$
$BiBr_3$ 50	200	0.25	2 s	Cs_3BiBr_6
BiI3 100	200	0.25	2 s	$Cs_3Bi_2I_9$
$SbCl_3$ 100	200	0.25	2 s	$Cs_3Sb_2Cl_9$
$SbBr_3$ 100	200	0.25	2 s	$Cs_3Sb_2Br_9$
SbI_3 100	200	0.25	2 s	$Cs_3Sb_2I_9$

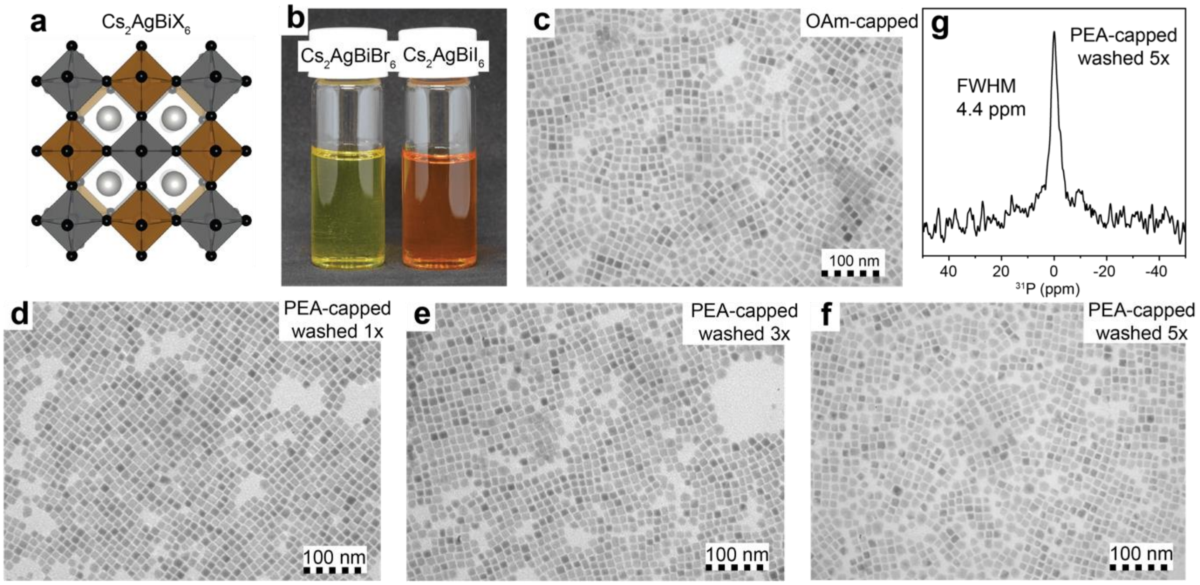

Supplementary Fig. 16 | Ligand exchange experiment for double perovskite Cs_2AgBiX_6 NCs. (**a**) NCs. **b**, $Cs_2AgBiBr_6$ and Cs_2AgBiI_6 colloids synthesized according to ref. 3 stabilized with PEA-C8C12 and PPA-C8C12 ligands, respectively, and purified. **c**, Initial $Cs_2AgBiBr_6$ NCs capped with OAm. **d**,**e**,**f**, $Cs_2AgBiBr_6$ NCs after ligand exchange with PEA-C8C12 and one (**d**), three (**e**), and five (**f**) steps of antisolvent purification. **g**, Liquid ^{31}P NMR of the $Cs_2AgBiBr_6$ NCs colloid in toluene-d8 demonstrating signal from phosphate PEA group bound to NCs surface.

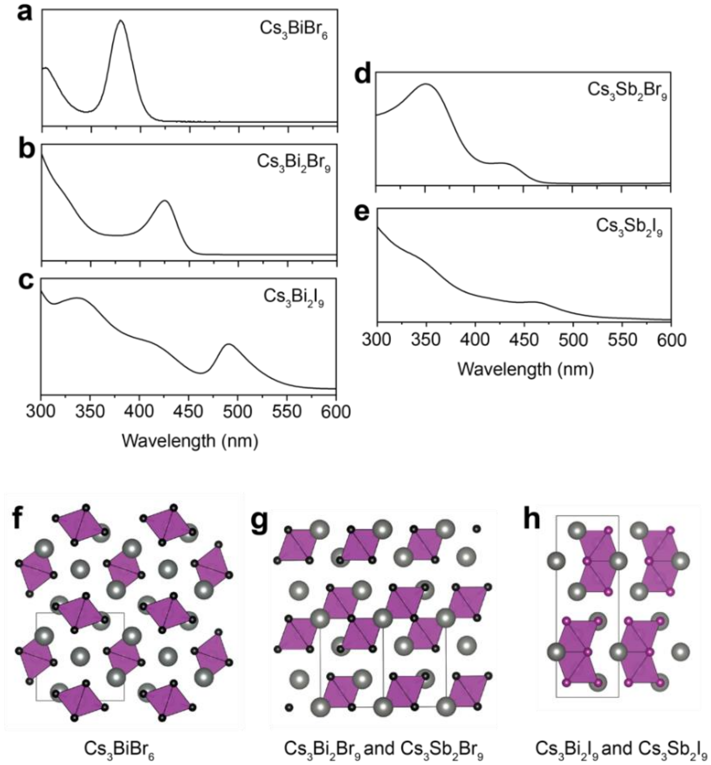

Supplementary Fig. 17 | Cs-Bi-X and Cs-Sb-X NCs stabilized by PEA ligands. a-e, Absorption spectra of various colloids from the Extended Data fig. 11. **f-h**, Crystal structures of the compounds from Cs-Bi-X and Cs-Sb-X families.

Supplementary Note 6. Purification of NCs

Purification of the NCs consists of precipitation with antisolvent and redispersion with a good solvent (single washing cycle, **Supplementary Fig. 11**). The maximum number of cycles depends on the binding strength of the ligand: for PEA ligands, a typical number of washing cycles is at least 10. The list of selected solvents and antisolvents is summarized in the **Supplementary Table 9**.

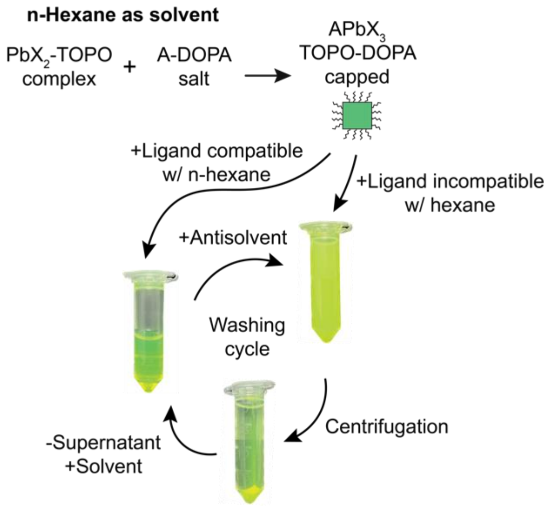

Supplementary Fig. 18 | A typical purification procedure following TOPO-DOPA synthesis. For ligands compatible with hexane, ligand-capped NCs are precipitated with antisolvent, centrifugated and redispersed in a suited solvent. NCs capped with ligands incompatible with hexane precipitate after ligand addition and can be collected by centrifugation, and further redispersed in a suitable solvent.

Supplementary Table 11. List of single solvents (not solvent mixtures) and antisolvents, in which NCs capped with PEA ligands with specific tail groups can be dispersed.

Tail group	Solvents	Antisolvents	Tail group	Solvents	Antisolvents
R1 $C_{18}H_{37}$	Chloroform	Acetone, Acetonitrile	R13 (7)	Chloroform, Acetone	Hexane
R2 $C_{18}H_{35}$	Hexanes, Toluene, Chloroform	Acetone, Acetonitrile	R14 (16)	Acetone, Acetylacetone	Hexane
R3 $C_{16}H_{33}$	Chloroform	Acetone, Acetonitrile	R15 (44)	Acetone, Acetylacetone	Hexane
R4 $C_{14}H_{29}$	Chloroform	Acetone, Acetonitrile	R16 (15, 5)	Acetone, Toluene	No suitable antisolvent
R5 $C_{12}H_{25}$	Chloroform	Acetone, Acetonitrile	R17 (9)	Acetone, Toluene	Acetonitrile
R6 $C_{20}H_{41}$	Hexanes	Ehylacetate, Acetone, Acetonitrile	R18 (OH, 13, 5, 2.5)	Ethanol, Acetone, Chloroform	Hexane
R7 $C_{16}H_{25}$	Toluene, Chloroform	Acetone, Acetonitrile	R19 (OH, 12)	Ethanol, Acetone, Chloroform	Hexane
R8 $C_{15}H_{23}$	Toluene, Chloroform	Acetone, Acetonitrile	R20 (OH, 11)	Propanol, Acetone, Toluene	Hexane
R9 $C_{12}H_{24}Br$	Chloroform, Tetrabromo-ethane	Acetone, Acetonitrile	R10 (7)	Toluene, Chloroform	Hexane, Acetone, Isopropanol
R21	Hexanes, Toluene, Chloroform	Acetone, Acetonitrile	R11 (22)	Toluene, Chloroform	Hexane, Isopropanol
			R12 (H, 47)	Toluene, Chloroform	Hexane, Isopropanol

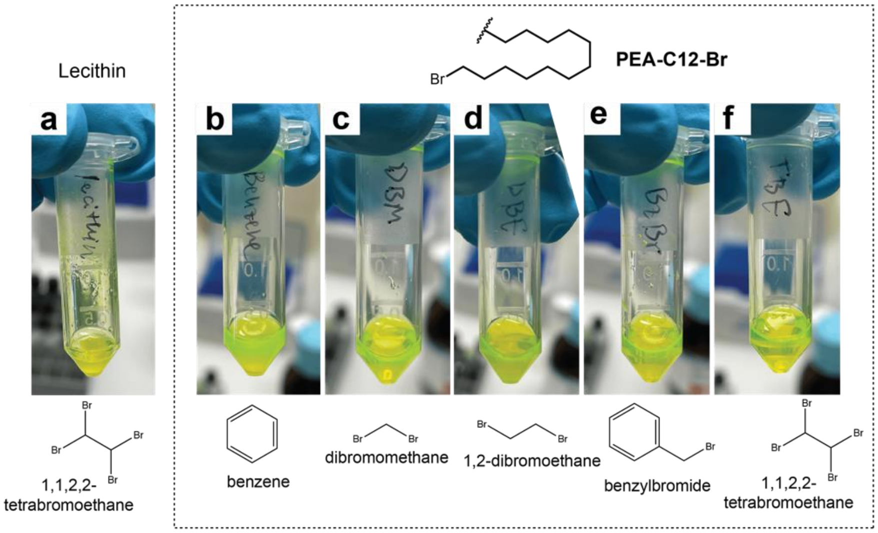

Supplementary Fig. 19 | Br-C12-PEA-capped $FAPbBr_3$ NCs. a-f, NCs redispersed in various brominated solvents (**b-f**). Lecithin-capped NCs in a control sample (**a**) do not redisperse in tetrabromoethane.

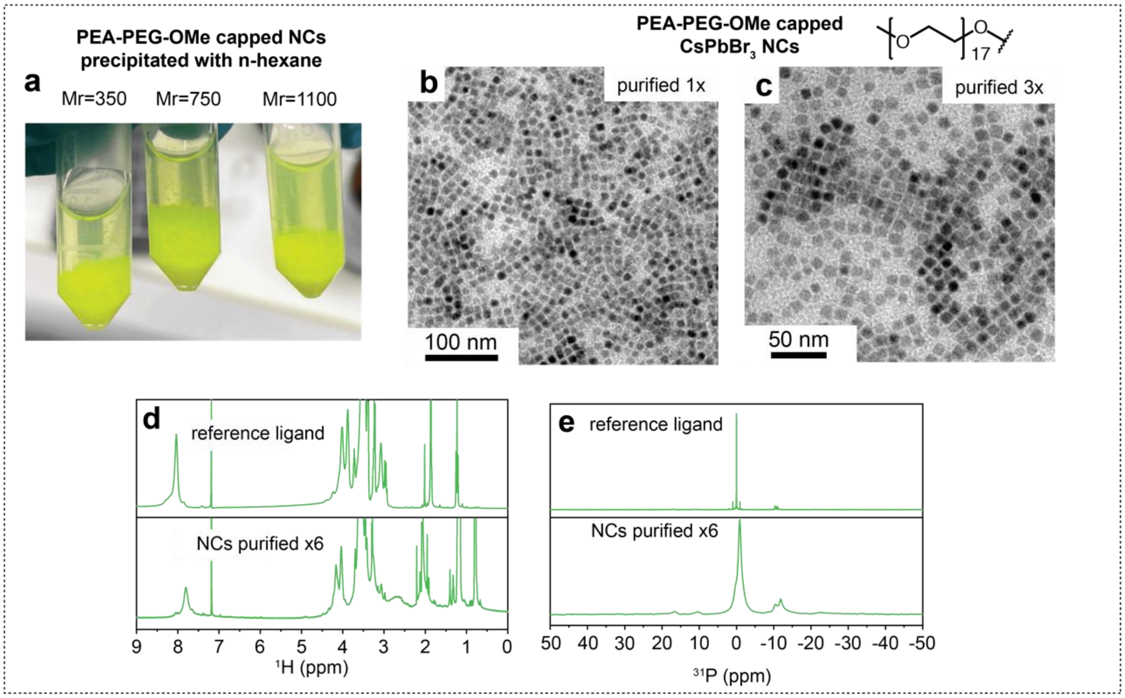

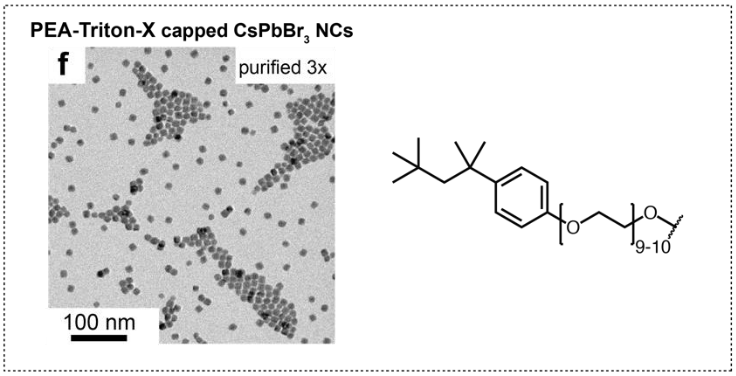

Supplementary Fig. 20 | Additional details for the PEG-PEA-capped $CsPbBr_3$ NCs. a, Strongly polar PEG-PEA-capped $CsPbBr_3$ NCs can be precipitated from acetone with n-hexane. **b-e,** TEM images of the $CsPbBr_3$ NCs capped with PEA-PEG-OMe washed once (**b**) or thrice (**c**) and drop-cast on the substrate from acetone. The key observation is the effect of the terminal group on the effective isolation of the impurity ligand. In the case of methoxy-termination, excess unbound ligand persists in the NMR spectra (**d,e**) and is also evident in TEM images as amorphous material. **f,** With a PEG ligand with a less polar Triton-X tail, NCs are readily purified (see **Supplementary Table 10** for details).

Supplementary Table 12. NCs capped with functionalized PEG tails and their compatibility with various solvents

Ligand / Solvent	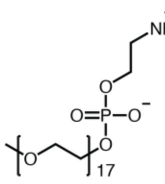 **PEA-PEG-OMe**	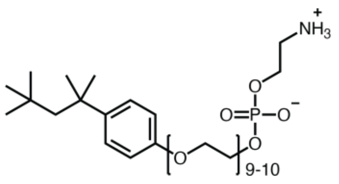 **PEA-Triton-X**	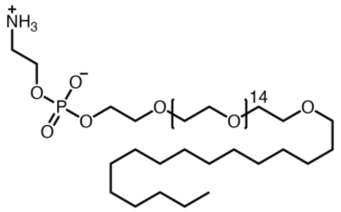 **PEA-Brij**
Hexane	Unstable, NCs precipitate immediately (used as anti-solvent)	Unstable, but NCs don't precipitate when added	Unstable, but NCs don't precipitate when added
Toluene	Start to precipitate	Stable	Stable
Acetone	Stable	Stable	Stable
Acetonitrile	Unstable, but NCs don't precipitate when added	Unstable, NCs precipitate immediately (used as anti-solvent)	Unstable, but NCs don't precipitate when added

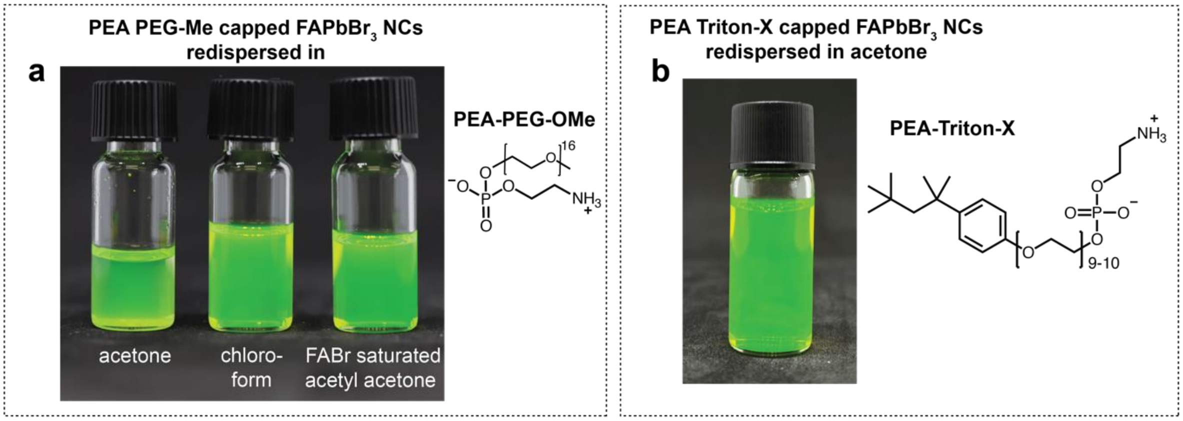

Supplementary Fig. 21 | PEG-PEA-capped $FAPbBr_3$ NCs. a, FABr is more soluble in acetone or acetylacetone compared to CsBr. $FAPbBr_3$ NCs capped with PEA PEG-Me are stable only in solvents where FABr solubility is low, *e.g.,* chloroform. To stabilize NCs in more polar solvents, the latter are first saturated with FABr to shift the chemical equilibrium. **b**, PEA-Triton-X ligand results in $FAPbBr_3$ NCs being stable in acetone without saturation with FABr.

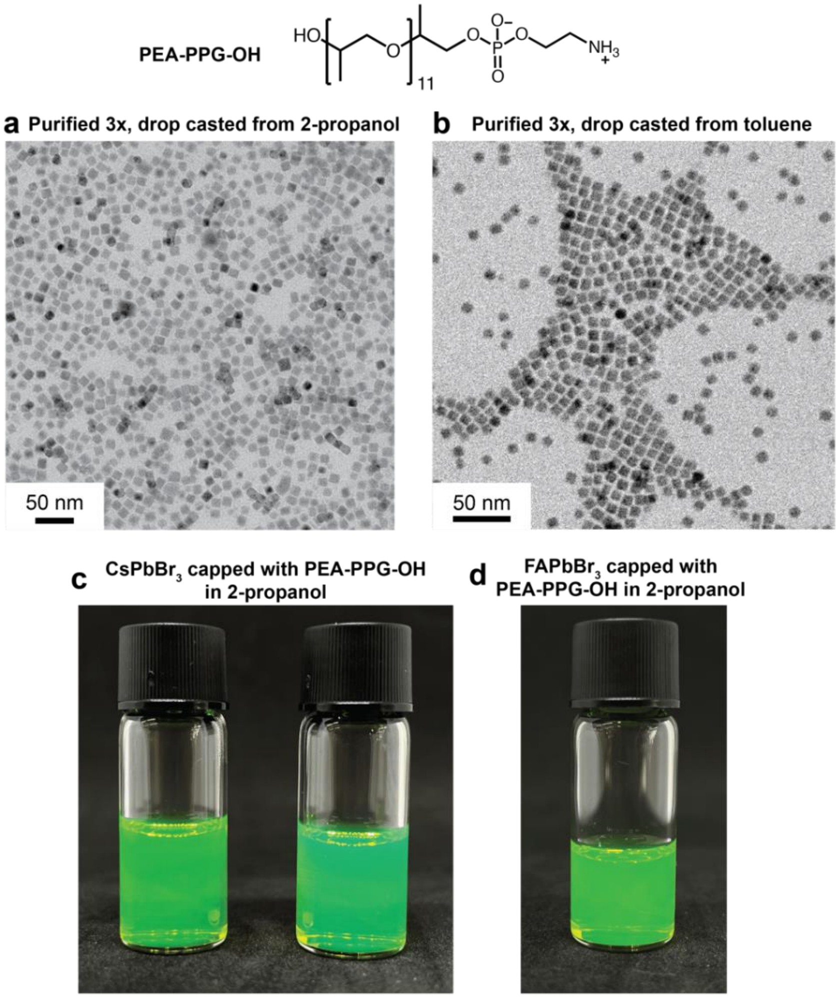

Supplementary Fig. 22 | PPG-PEA-capped NCs. a,b, TEM of the $CsPbBr_3$ NCs capped with PEA-PPG-OH, drop-cast from 2-propanol (**a**) and toluene (**b**). **c,d,** Photographs of $CsPbBr_3$ (**c**) and $FAPbBr_3$ (**d**) colloids capped stabilized with PEA-PPG-OH in 2-propanol.

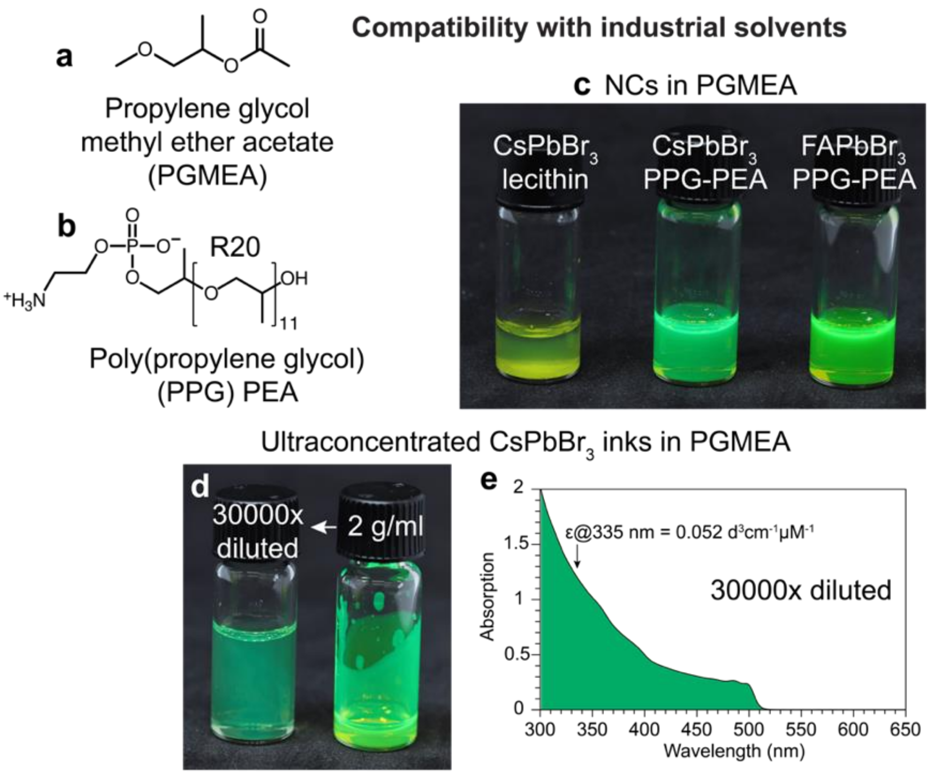

Supplementary Fig. 23 | Demonstration of poly(propylene glycol) PEA-capped perovskite NCs dispersibility in propylene glycol methyl ether acetate (PGMEA), a common solvent in semiconductor industry. a-b, Chemical formulas of PGMEA (a) and PEA-PPG ligand (b). **c**, Perovskite NCs in PGMEA. **d**, Diluted and ultraconcentrated PGMEA colloids of $CsPbBr_3$ NCs capped with PEA-PPG. **e**, Concentration estimation of the colloids.

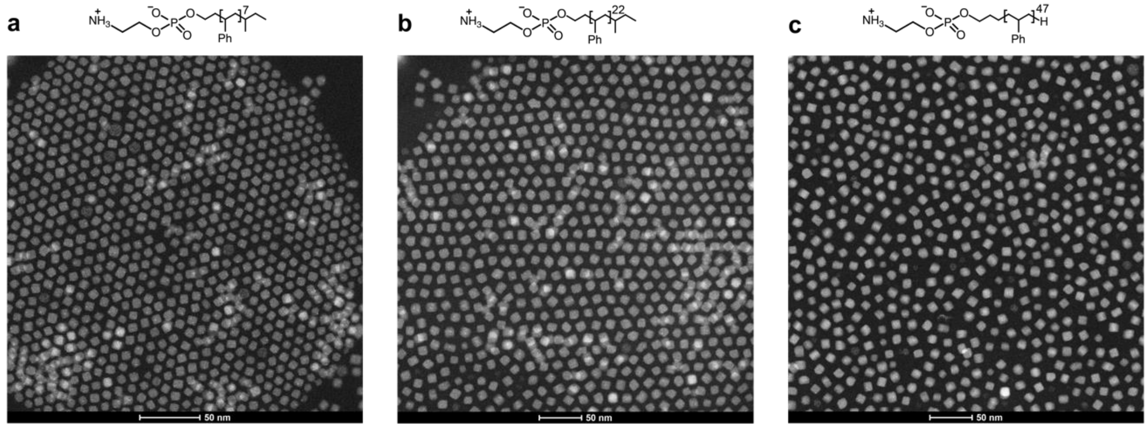

Supplementary Fig. 24 | Interparticle distance tuning by ligands. a-c, HAADF-STEM images of $CsPbBr_3$ NCs capped with PS-PEA ligands with various polystyrene length.

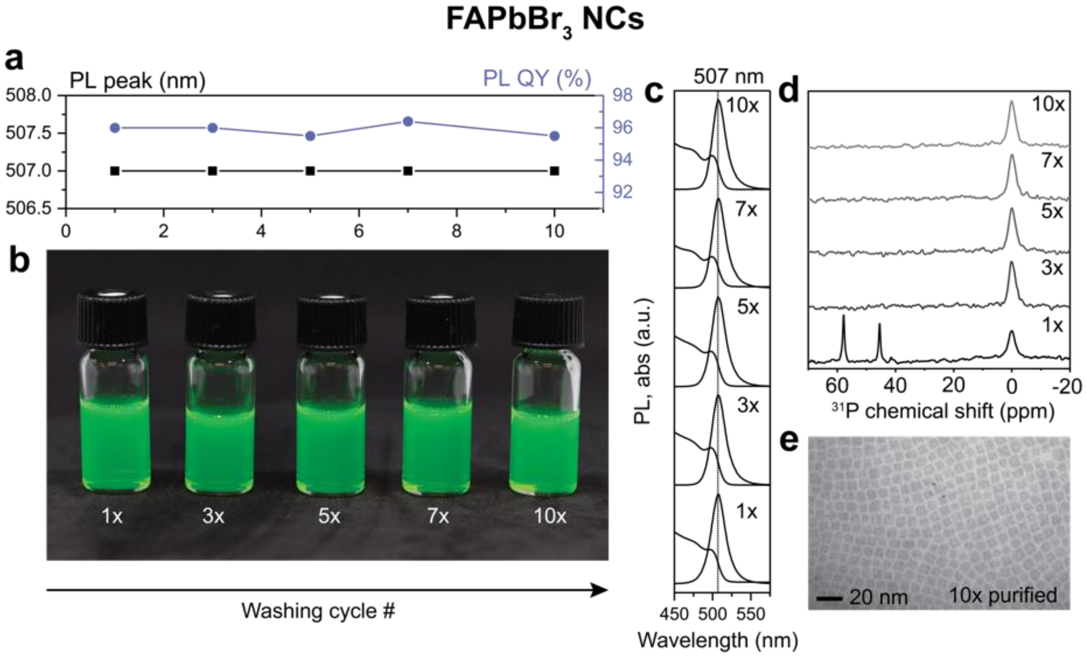

Supplementary Fig. 25 | Stability of PEA-capped $FAPbBr_3$ NCs after multiple purification cycles. a-e, $FAPbBr_3$ NCs capped with C8C12-PEA ligand used for stability testing. Characterization of $FAPbBr_3$ NCs purified using different number of washing cycles, including PL peak position and PLQY (a), visual appearance (b), absorption and PL spectra (c), 31P NMR spectra (d), and TEM of 10x purified NCs (e).

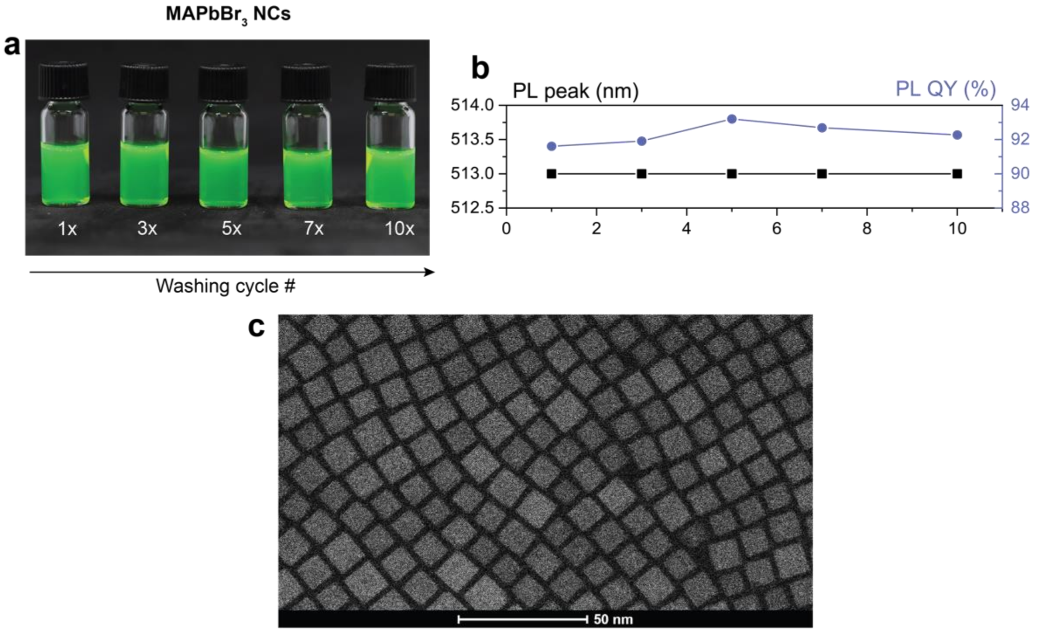

Supplementary Fig. 26 | Stability of $MAPbBr_3$ NCs. C8C12-PEA- capped $MAPbBr_3$ NCs in n-hexane (**a**) retain optical properties (**b**) over at least 10 consecutive washings with antisolvent (1 eq. of acetone). **c**,

High resolution STEM of $MAPbBr_3$ NCs washed 4x times with antisolvent.

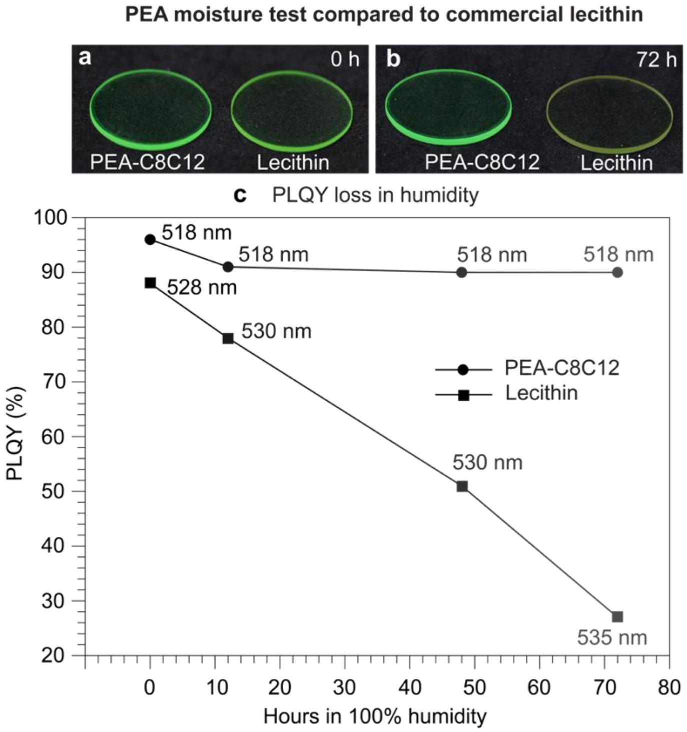

Supplementary Fig. 27 | Humidity test performed on $FAPbBr_3$ NCs films capped with PEA-C8C12 and commercial lecithin ligand. a, Initial films of $FAPbBr_3$ NCs. **b**, NCs films after 72 hours in 100% humidity chamber. **c**, PLQY dependence for NC films on the time spent in a humidity chamber.

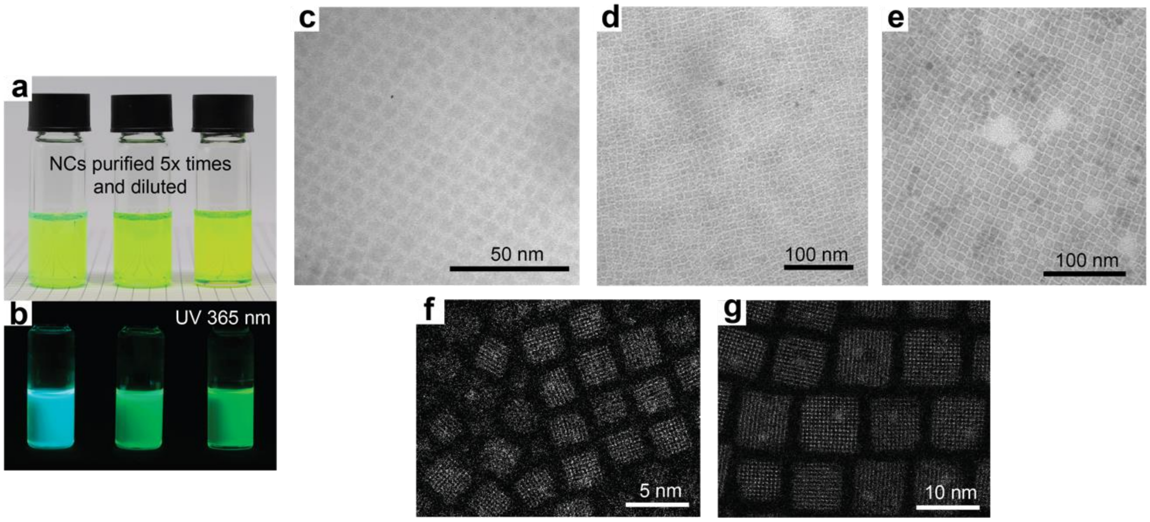

Supplementary Fig. 28 | Size-tunability of PEA-capped $FAPbBr_3$ NCs. a,b, Selected $FAPbBr_3$ NCs of various sizes (5.6 nm, 6.9 nm and 9.8 nm) stabilized by PEA-C8C12 ligand and purified 5 times with anti-solvent. **c,d,e,** TEM images of the corresponding samples. **f,g,** High-resolution HAADF-STEM images of 4.5 and 9.6 nm $FAPbBr_3$ NCs.

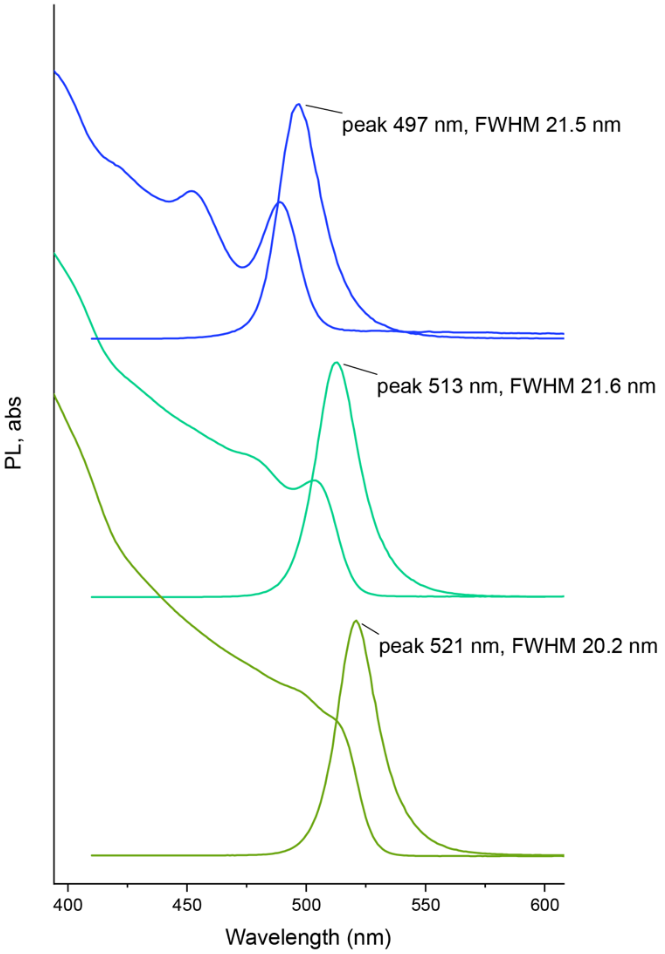

Supplementary Fig. 29 | Optical properties of $FAPbBr_3$ NCs. A series of C8C12-PEA-ligated NCs (see Supplementary Fig. 28), purified 5 times with anti-solvent.

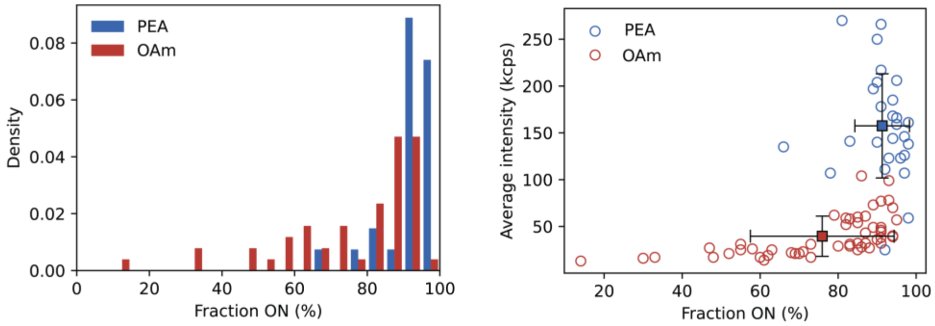

Supplementary Fig. 30 | Statistics of ON fraction across 78 NCs. Similar to Figure 5e,f the ON fraction was determined for 78 single NCs blinking traces of PEA- and OAm-capped $FAPbBr_3$ NCs. The mean ON fractions are 91 ± 3% for PEA and 76 ± 5% for OAm, where there errors denote 95% confidence intervals.

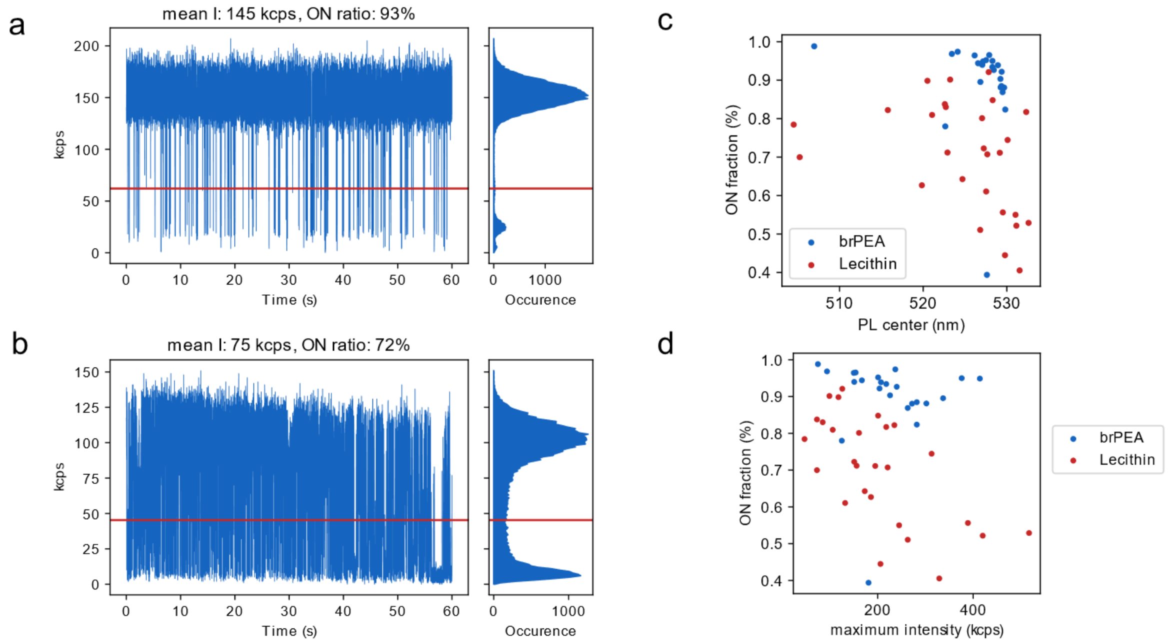

Supplementary Fig. 31 | Blinking of single $FAPbBr_3$ NCs capped with C8C12-PEA and lecithin, encapsulated in SEBS and measured under ambient conditions. a-b, The blinking trace of a representative PEA-capped QD (a) displays significantly suppressed blinking (in line with the main results reported in the main text) and (b) enhanced average intensity compared to the trace of a lecithin-capped QD. **c**, Dependence of the ON fraction on the central wavelength as an alias for the particle size and absorption cross-section. **d**, ON fraction as a function of maximum intensity observed in the blinking trace as an indicator of the number of excitations per unit of time. PEA-capped QDs systematically spend more time in their bright state independent of absorption cross section (size) and excitation level.

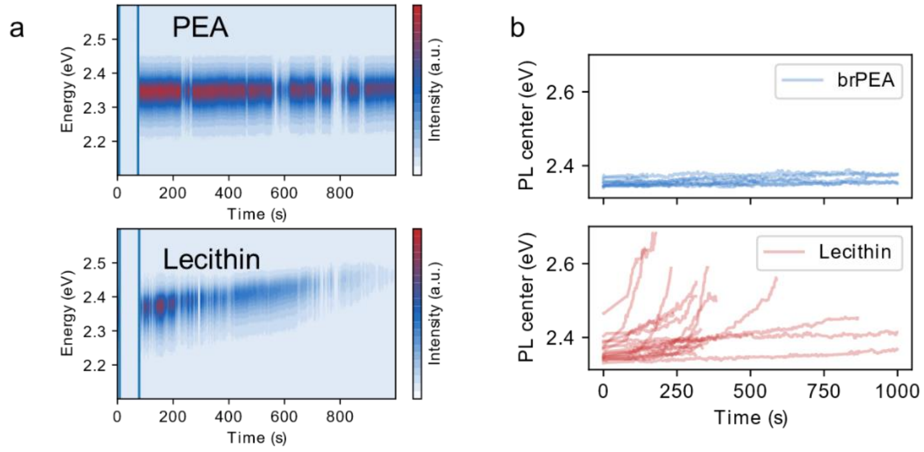

Supplementary Fig. 32 | Dynamical PL blueshift of single $FAPbBr_3$ NCs capped with C8C12-PEA and lecithin ligands, encapsulated in SEBS and measured under ambient conditions and similar excitation density. a, PL spectra series of representative PEA- (top) and lecithin-capped QDs (bottom). **b**, Evolution of PL central emission peaks for various single QDs from the two samples.

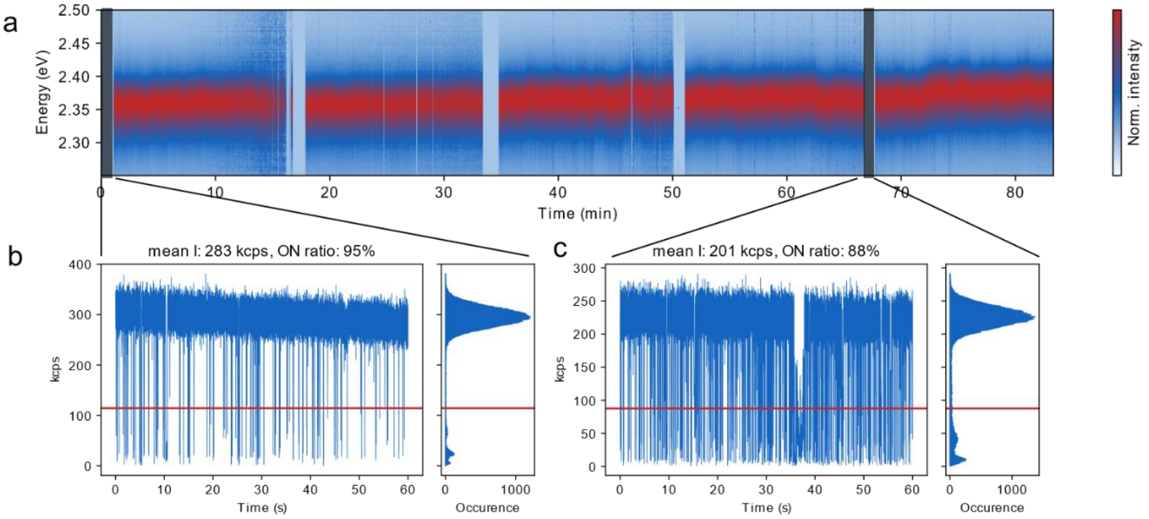

Supplementary Fig. 33 | Spectral stability of a single PEA-capped $FAPbBr_3$ NC in SEBS film measured under nitrogen atmosphere. a, Normalized PL spectra series recorded over 83 minutes. Low intensity regions correspond to the time interval, during which blinking traces are acquired. **b-c,** Blinking traces of the QD at the beginning of the measurement (b) and after ca. 65 minutes of continuous laser irradiation (c).

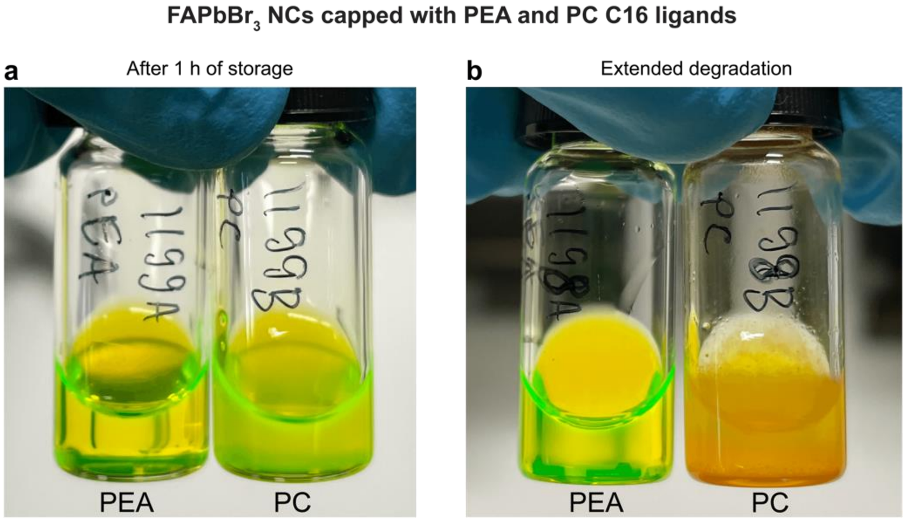

Supplementary Fig. 34 | Stability of C16-PC and C16-PEA-capped NCs. after 1 h (a) and12 h (b) from preparation. The same samples as used in the Main text Fig. 3.

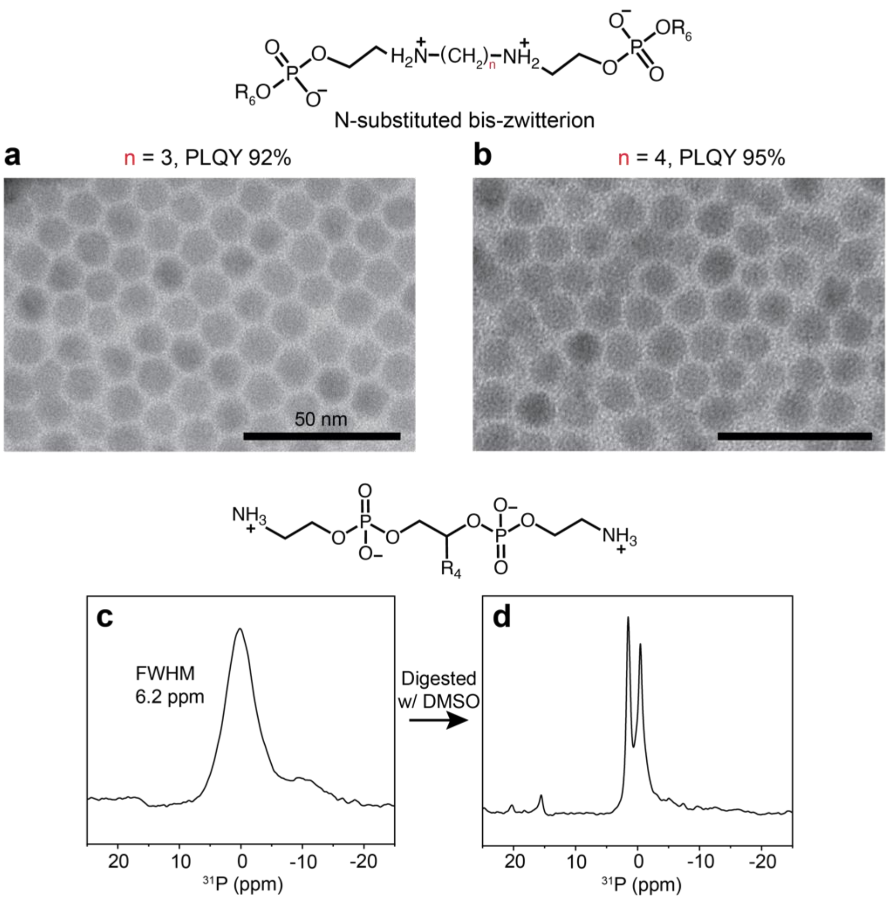

Supplementary Fig. 35 | $CsPbBr_3$ and $FAPbBr_3$ NCs capped with a bis-zwitterion. A pair of zwitterions might further improve ligand binding to the NCs surface. **a,b,** TEM of the $CsPbBr_3$ NCs capped with N-substituted bis-zwitterion with three (**a**) or four (**b**) $-CH_2-$ groups in the linker bridge. **c,d**, As found for $FAPbBr_3$, N-substituted bis-zwitterion does not provide satisfactory long-term colloidal stability, so a PEA bis-zwitterion was then devised. ^{31}P NMR of the surface-bound ligand is broadened (**c**), as expected from slower molecular tumbling. The ligand can be liberated by digesting NC core in DMSO, revealing two phosphate groups (**d**).

Supplementary Note 7. Direct synthesis of $FAPbBr_3$ NCs in the presence of a zwitterionic ligand.

NCs were synthesized according to general procedure that combines FA, Pb and halide precursors (**Supplementary Fig. 36**a).[16] Briefly, lead and FA precursors were solubilized in a high-boiling point apolar solvents (mesitylene, ODE) with the help of long alkyl chain acids (OA or DOPA) and a halide precursor (TOP-halogen complex) was injected.

Lead(II) oleate precursor, $Pb(OA)_2$. Lead(II)acetate trihydrate (4.6066 g, 12 mmol, 1 eq) and oleic acid (7.6 mL,24 mmol, 2 eq) were evacuated in a three-neck flask along with 16.4 mL of ODE at room temperature until the gas evolution stops and then further evacuated at 120 °C for 1 hour. This yields a 0.5M stock

solution of Pb-oleate in ODE. The solution turns solid when cooled to room temperature and was stored under argon and heated before use.

Formamidinium oleate, FAOA. Formamidinium acetate (0.521 g, 5 mmol) and oleic acid (2.5 ml) were combined in ODE (20 ml), heated to 100°C under Ar for 1 h and dried under vacuum at 50°C for additional 30 minutes. This yields a 0.2M stock solution of FAOA.

TOP-Br_2 precursor solution was prepared by mixing TOP (6 mL, 13 mmol, 1 eq) with Br_2 (0.6 mL, 11.5 mmol, 0.88 eq). The reaction between the two components is exothermic and requires vigorous stirring due to the product being highly viscous (white, almost solid). Mesitylene (18.7 mL) as added to the reaction, yielding a 0.46M light-yellow stock solution. The reaction was carried out in a Schlenk flask under Argon.

In a typical $FAPbBr_3$ NCs synthesis, 1 eq. of FAOA (1 ml, 0.2M) and 1.6 eq. of $Pb(OA)_2$ (0.5 ml 0.4M) precursors were combined with 5 ml of mesitylene along with 0.25 eq. of PC ligand or 0.25 eq. of PEA ligand of choice (added as dry solids). The reaction vessel was heated to required temperature (typically 70°C for PC and 90°C for PEA) and TOP-Br_2 (0.5 ml) was injected. The reaction was cooled with ice bath immediately, yielding a bright green colloid (**Supplementary Fig. 36b**). NCs were purified by adding 1-2 eq. of acetonitrile, centrifugated and redispersed in a solvent of choice (*e.g.,* hexane for C8C12-PEA). The purification procedure was repeated up to 5 times to remove precursors and unbound ligands, resulting in NCs colloids capped with PC and PEA ligands (**Supplementary Fig. 36c-d**). PEA-capped $FAPbBr_3$ NCs retain colloidal stability upon extended storage, while PC-capped NCs precipitate and sinter over couple days.

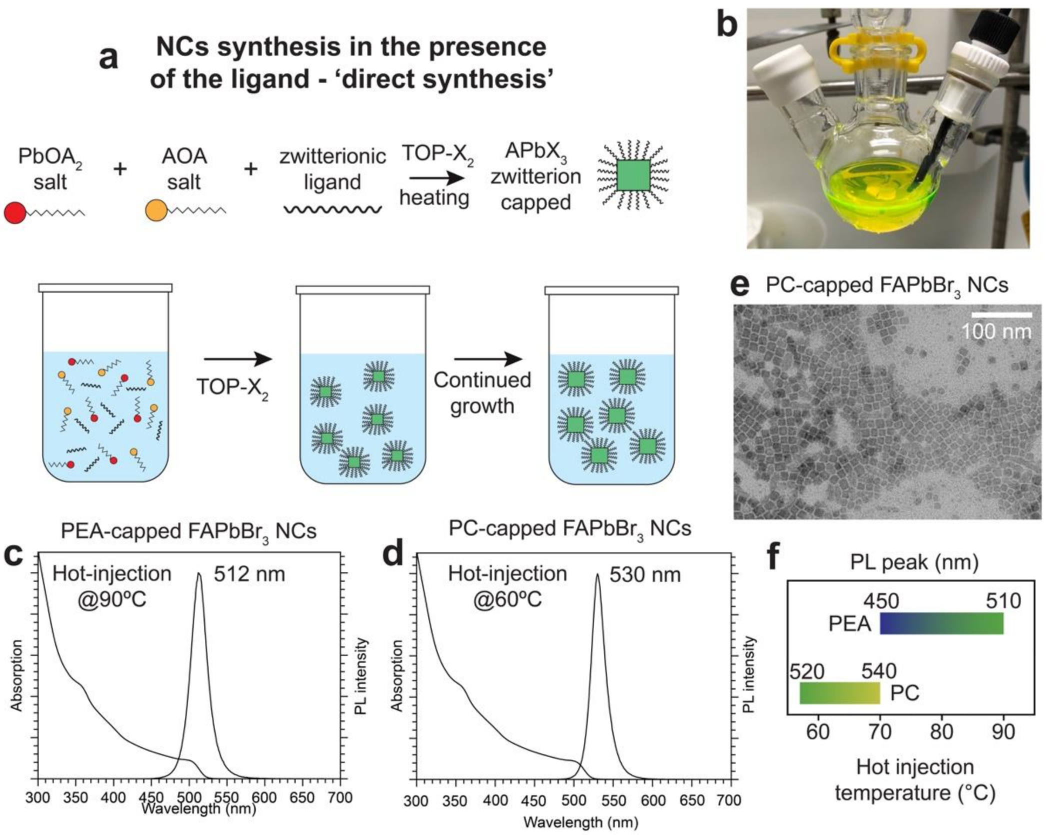

Supplementary Fig. 36 | 'Direct' hot-injection synthesis of perovskite NCs. **a,** Synthetic scheme of the hot-injection synthesis of $APbX_3$ NCs in the presence of phospholipid zwitterionic ligand using TOP-X_2 precursor, as adapted from our earlier report[16]. **b**, $FAPbBr_3$ NCs synthesized in the presence of C16-PC ligand. **c,d**, Optical properties of $FAPbBr_3$ NCs synthesized in the presence of C8C12-PEA (**c**) and C16-PC (**d**). **e**, TEM image of $FAPbBr_3$ NCs synthesized in the presence of C16-PC. **f**, A summary showing the range of PL peak energies (reflecting NCs size) achievable for direct synthesis with C8C12-PEA and C16-PC ligands by varying the reaction temperature. Stronger binding PEA-ligands afford smaller NC sizes at the same synthesis temperature.

Supplementary Table 13. Long-term colloidal stability in different solvents

NCs composition	Size range tested	Ligand	Solvent	Colloidal stability[#]
$FAPbBr_3$	5-10 nm	C8C12-PEA	Hexane, octane	>12 months
$CsPbBr_3$	5-10 nm	C8C12-PEA	Hexane, octane	>12 months
$MAPbBr_3$	8-10 nm	C8C12-PEA	Hexane, octane	>12 months
$FAPbBr_3$	8 nm	PEG-PEA, PPG-PEA	Acetone, n-butanol, iso-propanol	3-4 months
$CsPbBr_3$	8 nm	PEG-PEA, PPG-PEA	Acetone, n-butanol	4-6 months
			Ethanol	1 months

			Methanol	1 months
$FAPbX_3$, X=Cl-Br	9 nm	C8C12-PEA	Hexane	>12 months
$CsPbX_3$, X=Cl-Br	8-10 nm	C8C12-PEA	Hexane	>12 months
$MAPbX_3$, X=Cl-Br	8-10 nm	C8C12-PEA	Hexane	>12 months
$FAPbX_3$, X=Br-I	7-9 nm	C8C12-PPA	Hexane	3-4 months
$CsPbX_3$, X=Br-I	7-10 nm	C8C12-PEA	Hexane	1 month before yellow phase appears
$MAPbX_3$, X=Br-I	9 nm	C8C12-PEA	Hexane	>12 months

[#]Colloidal stability reported for concentration range 8-10 mg/ml, stored under ambient conditions. Times of the first degradations signs (precipitation) indicated.

References

1 Rodová, M., Brožek, J., Knížek, K. & Nitsch, K. Phase transitions in ternary caesium lead bromide. *J. Therm. Anal. Cal.* **71**, 667-673, (2003).

2 Hanusch, F. C. *et al.* Efficient planar heterojunction perovskite solar cells based on formamidinium lead bromide. *J. Phys. Chem. Lett.* **5**, 2791-2795, (2014).

3 Kühne, T. D. *et al.* CP2K: An electronic structure and molecular dynamics software package - Quickstep: Efficient and accurate electronic structure calculations. *J. Chem. Phys.* **152**, (2020).

4 Bussi, G., Donadio, D. & Parrinello, M. Canonical sampling through velocity rescaling. *J. Chem. Phys.* **126**, (2007).

5 Nibali, V. C., Branca, C., Wanderlingh, U. & D'Angelo, G. Intermolecular hydrogen-bond interactions in DPPE and DMPC phospholipid membranes revealed by far-infrared spectroscopy. *Appl. Sci.-Basel* **11**, (2021).

6 Fringeli, U. P. & Günthard, H. H. Infrared membrane spectroscopy. In *Membrane Spectroscopy* (ed Ernst Grell) 270-332 (Springer Berlin Heidelberg, 1981).

7 Dreissig, I., Machill, S., Salzer, R. & Krafft, C. Quantification of brain lipids by FTIR spectroscopy and partial least squares regression. *Spectrochim. Acta A* **71**, 2069-2075, (2009).

8 Cernescu, A. *et al.* Label-free infrared spectroscopy and imaging of single phospholipid bilayers with nanoscale resolution. *Anal. Chem.* **90**, 10179-10186, (2018).

9 Derenne, A., Claessens, T., Conus, C. & Goormaghtigh, E. Infrared spectroscopy of membrane lipids. In *Encyclopedia of Biophysics* (ed Gordon C. K. Roberts) 1074-1081 (Springer Berlin Heidelberg, 2013).

10 Bellamy, L. J. & Beecher, L. The infra-red spectra of organo-phosphorus compounds. Part III. Aliphatic acids and compounds related to natural products. *J. Chem. Soc.*, 728-732, (1953).

11 Harmon, K. M. & Akin, A. C. Hydrogen bonding Part 38. IR and thermodynamic study of phosphorylcholine chloride calcium salt tetrahydrate and monohydrate. *J. Mol. Struct.* **249**, 173-179, (1991).

12 Chane-Ching, J. Y., Lebugle, A., Rousselot, I., Pourpoint, A. & Pellé, F. Colloidal synthesis and characterization of monocrystalline apatite nanophosphors. *J. Mater. Chem.* **17**, 2904-2913, (2007).

13 Glaser, T. *et al.* Infrared spectroscopic study of vibrational modes in methylammonium lead halide perovskites. *J. Phys. Chem. Lett.* **6**, 2913-2918, (2015).

14 Liu, S. *et al.* Membrane-destabilizing ionizable phospholipids for organ-selective mRNA delivery and CRISPR–Cas gene editing. *Nat. Mater.* **20**, 701–710, (2021).

15 Zhang, Y. N., Shah, T., Deepak, F. L. & Korgel, B. A. Surface science and colloidal stability of double-perovskite $Cs_2AgBiBr_6$ nanocrystals and their superlattices. *Chem. Mater.* **31**, 7962-7969, (2019).

16 Krieg, F. *et al.* Colloidal $CsPbX_3$ (X = Cl, Br, I) nanocrystals 2.0: zwitterionic capping ligands for improved durability and stability. *ACS. Energy Lett.* **3**, 641-646, (2018).
17 Krieg, F. *et al.* Stable ultraconcentrated and ultradilute colloids of $CsPbX_3$ (X = Cl, Br) nanocrystals using natural lecithin as a capping ligand. *J. Am. Chem . Soc.* **141**, 19839-19849, (2019).
